# Therapeutic efficacy and mechanisms of Xuebijing in *Acinetobacter baumannii* infection based on meta-analysis and integrated pharmacology approaches

**DOI:** 10.3389/fphar.2025.1598359

**Published:** 2025-05-23

**Authors:** Jiaqi Li, Yihan Sun, Hao Wen, Boying Xiao, Jianming Wang, Ziqi Li, Lei Wang, Meiyu Yu

**Affiliations:** ^1^ Department of Trauma Center, The First Hospital of China Medical University, Shenyang, Liaoning, China; ^2^ Department of General Surgery, The First Hospital of China Medical University, Shenyang, Liaoning, China

**Keywords:** Xuebijing, *Acinetobacter baumannii*, meta-analysis, network pharmacology, molecular docking, molecular dynamics simulations

## Abstract

**Background:**

Xuebijing (XBJ) is a traditional Chinese medicine widely used in China for managing sepsis, systemic inflammatory response syndrome, and multiple organ dysfunction secondary to severe infections. This study aimed to assess the therapeutic efficacy and safety of XBJ as an adjuvant treatment for *Acinetobacter baumannii* (*A. baumannii*) infections through meta-analysis, and to explore its potential mechanisms via integrated pharmacological approaches.

**Materials and methods:**

A systematic search was conducted across multiple databases for randomized controlled trials (RCTs) evaluating XBJ in *A. baumannii* infections up to 9 February 2025. Meta-analyses were conducted to synthesize clinical outcomes, and evidence certainty was assessed using the GRADE framework. Network pharmacology, molecular docking, and molecular dynamics simulations were used to evaluate the interactions between active ingredients of XBJ and protein targets in *A. baumannii* infections.

**Results:**

A total of 11 RCTs involving 1,035 patients met the inclusion criteria. Meta-analysis demonstrated that XBJ significantly improved clinical outcomes, with a higher overall effective rate (RR = 1.20, 95% CI: 1.13–1.27, *P* < 0.01) and enhanced bacterial clearance (RR = 1.44, 95% CI: 1.23–1.68, *P* < 0.01) compared to conventional treatment alone. Additionally, XBJ treatment was associated with marked reductions in inflammatory markers, including C-reactive protein (CRP) (SMD = −2.12), procalcitonin (PCT) (SMD = −2.28), and white blood cell (WBC) count (SMD = −1.00) (all *P* < 0.01). Notably, no serious adverse drug events were reported. Mechanistic investigations identified three active ingredients of XBJ including scutellarin, salvianolic acid C, and isosalvianolic acid C, as potential modulators of MMP9 and TLR4, suggesting the role of XBJ in attenuating inflammatory responses and improving infection outcomes.

**Conclusion:**

XBJ appears effective and safe as an adjuvant therapy for *A. baumannii* infections, but further high-quality RCTs are warranted to validate these findings.

**Systematic Review Registration:**

https://www.crd.york.ac.uk/prospero/, identifier CRD42023486389.

## 1 Introduction

*Acinetobacter baumannii* (*A. baumannii*) has emerged as a critical global healthcare challenge, primarily due to its extraordinary capacity for developing resistance to multiple antimicrobial agents, rendering infections increasingly refractory to treatment (Marino et al., 2024). As a major nosocomial pathogen, it is a leading cause of ventilator-associated pneumonia, bloodstream infections, and urinary tract infections, particularly in critically ill patients (Cavallo et al., 2023; Diao et al., 2024). These infections often progress rapidly to sepsis and septic shock (Niu et al., 2023), driven by an initial phase of immune evasion that triggers an excessive inflammatory response, largely mediated through lipopolysaccharide (LPS)-Toll-like receptor 4 (TLR4) signaling (Sabatini et al., 2024). However, the growing threat of antimicrobial resistance continues to narrow the available therapeutic options. Carbapenems, once the mainstay of therapy, now frequently fail due to widespread resistance. Polymyxins, though still active against some strains, are limited by their nephrotoxicity, neurotoxicity, and the emergence of resistance (Novovic and Jovcic, 2023). Tigecycline, initially considered a promising alternative, is undermined by poor plasma concentrations, inadequate tissue penetration, and inconsistent clinical efficacy (Sun et al., 2023). Moreover, resistance also compromises sulbactam (Principe et al., 2022), β-lactam/β-lactamase inhibitors, and aminoglycosides (Kyriakidis et al., 2021), further narrowing the therapeutic arsenal. Given these challenges, there is an urgent need for alternative or adjunctive therapies that not only improve antimicrobial efficacy but also mitigate the excessive inflammatory responses (Cirino et al., 2023).

Xuebijing (XBJ), a novel Chinese patent medicine developed based on the “bacteria-toxin-inflammation” theory, is formulated from equal proportions of five medicinal plants—*Paeonia lactiflora* Pall. (Chishao), *Ligusticum striatum* DC. (Chuanxiong), *Salvia miltiorrhiza* Bunge (Danshen), *Carthamus tinctorius* L. (Honghua), and *Angelica sinensis* (Oliv.) Diels (Danggui)—all verified in the “World Flora Online” on 13 January 2025 (Ma et al., 2009). XBJ was reported to exert detoxifying, antioxidative and immune-modulating effects by inhibiting inflammatory mediators and attenuating endotoxins, and has been approved as a State Category II New Drug by the China Food and Drug Administration for clinical use in sepsis (Song et al., 2019; Yin and Li, 2014). Recent clinical investigations suggested that XBJ may also serve as an effective and safe adjuvant therapy for *A. baumannii* infections (Bai et al., 2017; Zhang et al., 2017; Ma et al., 2018; Dong, 2019; He et al., 2019; Liu et al., 2019; Liu, 2020; Ma et al., 2020; Pang et al., 2021; Niu and Zhu, 2022; Ma et al., 2024). However, current evidence is limited by heterogeneity and lacks comprehensive synthesis.

The present study aims to systematically review and meta-analyze the existing clinical evidence on XBJ as an adjuvant therapy for *A. baumannii* infections, and to further investigate its underlying mechanisms using an integrated pharmacological approach. These findings are expected to provide a more robust scientific foundation for the clinical application of XBJ, with the potential to improve treatment outcomes and alleviate the growing healthcare burden associated with *A. baumannii*.

## 2 Materials and methods

### 2.1 Protocols and registration

This systematic review and meta-analysis followed the methodological guidelines of the Preferred Reporting Items for Systematic Reviews and Meta-Analyses (PRISMA) (Page et al., 2021) to ensure methodological rigor. Moreover, the research protocol was prospectively registered with the International Prospective Register of Systematic Reviews (PROSPERO) under registration number CRD42023486389.

### 2.2 Search strategy

A comprehensive search was systematically conducted across both English and Chinese databases, covering the period from their inception to February 2025. The English databases included PubMed, Embase, Cochrane Library, EBSCO, and Web of Science, while key Chinese databases—China National Knowledge Infrastructure (CNKI), Chinese Biomedicine Literature Database (CBM), Chinese Science and Technology Journal Database (VIP), and Wanfang Database—were also thoroughly examined. The search strategy combined medical subject headings (MeSH) with free-text terms, ensuring a detailed analysis of titles, abstracts, and keywords. Specifically, the terms (“*Acinetobacter baumannii*” OR “*A. baumannii*”) AND (“Xuebijing” OR “XBJ”) were applied, with modifications tailored to the indexing systems of individual databases. To further enhance completeness, reference lists of included studies and relevant systematic reviews/meta-analyses were manually screened. Detailed search strategies are provided in Supplementary Material 1.

### 2.3 Inclusion and exclusion criteria

Inclusion criteria: Clinical trials assessing the therapeutic efficacy of XBJ in combination with other standard treatments for *A. baumannii* infection were eligible for inclusion, provided that the effects of XBJ could be clearly differentiated from those of other interventions. Eligible studies were required to report at least one of the following outcomes: overall effective rate, bacterial clearance rate, levels of inflammatory markers (including white blood cell (WBC) count, C-reactive protein (CRP) level, and procalcitonin (PCT)), or adverse drug events. Only randomized controlled trials (RCTs) published in English or Chinese were considered for inclusion.

Exclusion Criteria: Studies that were non-clinical, reviews, case reports, or conference abstracts were excluded. Non-randomized studies of interventions (NRSIs) were also excluded. Furthermore, studies with insufficient outcome reporting, significant methodological flaws, duplicate publications, or irrelevant data were excluded from this analysis.

### 2.4 Literature screening and data extraction

Two evaluators (YHS and JQL) independently screened the literature, extracted data, and cross-verified their findings. Any discrepancies were resolved through discussion or, when necessary, consultation with a third reviewer (HW) to reach a consensus. The screening process followed a stepwise approach, beginning with an initial assessment of titles and abstracts to exclude clearly irrelevant studies, followed by a full-text review based on predefined inclusion criteria. When key data were missing or unclear, the original authors were contacted via telephone or email for clarification or additional information. The extracted data encompassed study characteristics (author, publication year, country, study design, sample size, participant demographics, and specific disease conditions), intervention details (single dose, frequency, daily dosage, treatment duration, and total dosage of XBJ), and outcome measures (overall effective rate, bacterial clearance rate, and inflammatory markers such as CRP, PCT, and WBC), along with adverse drug events. Additionally, information relevant to the risk of bias assessment, including methods of randomization, allocation concealment, and other methodological aspects, was systematically collected.

### 2.5 Quality assessment

The risk of bias for all included studies was independently assessed following the guidelines of the *Cochrane Handbook* (version 6.4) (JPT et al., 2023). The methodological quality of RCTs was evaluated using the revised tool for risk of bias in randomized trials (RoB 2) tool, a revised framework for assessing bias in randomized trials (Sterne et al., 2019). Two researchers (HW and JMW) independently conducted the assessments, classifying studies as having low, some concerns, or high risk of bias. Any discrepancies were resolved through discussion or, if necessary, by consulting the supervisor (MYY) for consensus. The results were visualized using the R package “robvis”.

### 2.6 Data analysis

Meta-analysis was conducted using the “meta” (Balduzzi et al., 2019), “metafor” (Viechtbauer, 2010), and “dmetar” (Harrer et al., 2019) packages in R software (version 4.3.2). Risk ratios (RR) was employed for categorical variables, while standardized mean differences (SMD) was used for continuous variables, with a 95% confidence interval (95% CI) as the measure of effect size. Heterogeneity was assessed using the Cochrane Q and I^2^ statistics, with a *P*-value >0.1 and I^2^ < 50% indicating low heterogeneity, warranting the use of a fixed-effects model. Conversely, if *P* ≤ 0.1 and I^2^ ≥ 50%, significant heterogeneity was assumed, necessitating the application of a random-effects model. Sensitivity analysis was performed to evaluate the influence of individual studies on overall heterogeneity. To further explore sources of heterogeneity and assess the impact of key factors on the overall effect, subgroup analysis was conducted when at least six studies were available. Publication bias was assessed using Egger’s test, funnel plots, and the trim-and-fill method.

Trial sequential analysis (TSA) was performed to assess potential errors arising from limited sample sizes (Wang et al., 2023). Graphical representations depicted sample size on the x-axis and cumulative Z-scores on the y-axis, with parallel lines representing conventional significance thresholds. The required information size (RIS) was automatically estimated with a pre-specified type I error rate of 5%. Robustness was determined by the intersection of the cumulative Z-curve with both conventional and TSA boundaries, indicating whether further studies were necessary.

### 2.7 Evidence quality assessment

In this study, the quality and relevance of evidence were systematically evaluated using the GRADE (Grading of Recommendations Assessment, Development, and Evaluation) framework. The GRADEpro software was employed to assess the certainty of each outcome in accordance with established guidelines. This assessment incorporated key methodological domains, including study design, risk of bias, inconsistency across findings, indirectness of evidence, and imprecision of estimates. Based on these criteria, evidence certainty was classified into four levels: high, moderate, low, or very low. To further refine the evaluation, outcome importance was rated on a nine-point scale, stratified into three significance categories: non-critical, important, and critical. This structured approach ensured a rigorous appraisal of both the strength and applicability of the evidence, providing a robust foundation for interpreting the findings within a broader scientific and clinical context.

### 2.8 Network pharmacology

The bioactive ingredients of XBJ were identified through previous published mass spectrometry-based studies (Huang et al., 2011; Jiang et al., 2013; Sun et al., 2017; Zuo et al., 2017a; Zuo et al., 2017b; Zuo et al., 2019; Yu et al., 2022; Li et al., 2023), and subsequently analyzed using SwissTargetPrediction (Daina et al., 2019) to predict associated protein targets of XBJ ingredients. *A. baumannii*-related genes were retrieved from the GEO dataset GSE69528 (Pankla et al., 2009) and reanalyzed with the limma package in R to identify differentially expressed genes (DEGs). The intersection of predicted protein targets of XBJ ingredients and A. baumannii-related genes was visualized using a Venn diagram. To explore molecular interactions, the Search Tool for the Retrieval of Interacting Genes/Proteins (STRING) database (https://cn.string-db.org/) and Cytoscape (version 3.10.1) were employed to construct the protein-protein interaction (PPI) network. The “analyze network” function in Cytoscape was applied to score the network, with the two nodes exhibiting the highest degree selected as key targets.

### 2.9 Molecular docking

Molecular docking was performed using AutoDock Vina (version 1.5.7) (Trott and Olson, 2010) to predict ligand-target interactions. Crystal structures of target proteins were retrieved from the Protein Data Bank (PDB) (https://www.rcsb.org/), while 3D ligand structures were batch downloaded from the PubChem database (https://pubchem.ncbi.nlm.nih.gov/) using PubChemPy in Python (version 3.11.7). Docking results were visualized and analyzed using PyMOL (version 2.4.0) and Discovery Studio (version 2019).

### 2.10 Molecular dynamics simulation

Molecular dynamics simulations were conducted using GROMACS (version 2023.3) to evaluate the stability of protein-ligand complexes. Protein and ligand structures were separately processed, with topology files and simulation boxes generated using the pdb2gmx and gmx editconf commands, respectively. The system underwent initial energy minimization via the steepest descent method, followed by 100,000 steps of isothermal-isovolumetric (NVT) and isothermal-isobaric (NPT) equilibration, with coupling constants set at 0.1 ps and durations of 100 ps. Subsequently, a free dynamics simulation was performed with a 2 fs time step, extending up to 100 ns using the gmx grompp and gmx mdrun commands. Conformational data were continuously recorded to monitor the stability of the protein-ligand complexes.

## 3 Results

### 3.1 Literature search and study selection

**FIGURE 1:**
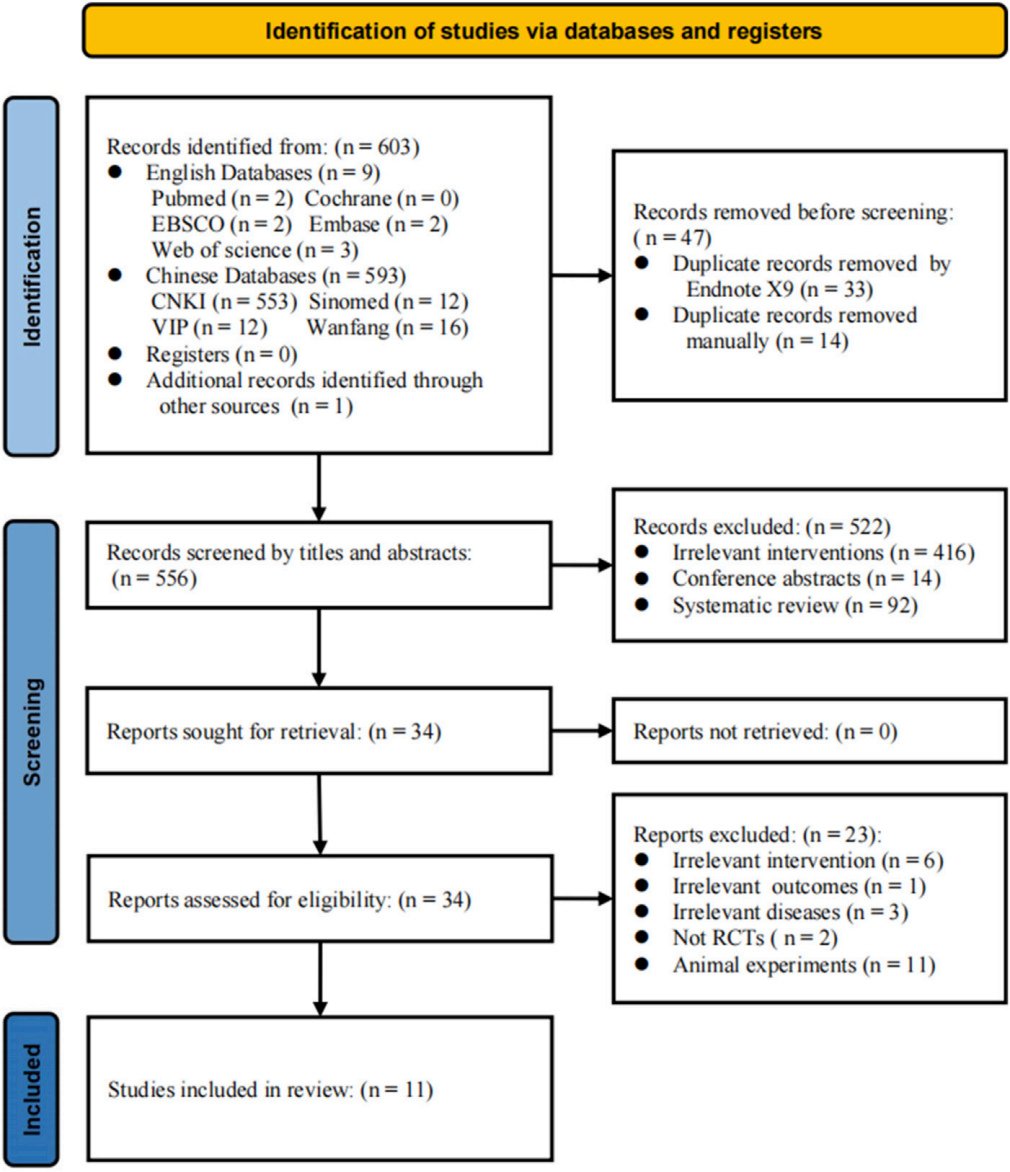
PRISMA flow diagram of the study selection process. The progression of the review process from the initial literature search to the ultimate meta-analysis. Each phase meticulously outlines the number of studies involved along with the rationale for study inclusion and exclusion.

A total of 603 relevant articles were imported into EndNote X9. Using the “find duplicates” feature, 33 duplicate records were automatically removed, with an additional 14 eliminated through manual screening. Among the remaining 556 articles, a review of titles and abstracts resulted in the exclusion of 522 studies, including 416 that did not meet the experimental requirements, interventions, or methods, 92 review articles, and 14 conference abstracts. A full-text review of the remaining 34 articles led to further exclusions: 6 for non-compliance with control principles, 1 for not meeting outcome criteria, 3 for non-compliance with diagnostic standards, 2 for not being RCTs, and 11 for being animal studies (Figure 1). Ultimately, 11 studies were included in the meta-analysis (Bai et al., 2017; Zhang et al., 2017; Ma et al., 2018; Dong, 2019; He et al., 2019; Liu et al., 2019; Liu, 2020; Ma et al., 2020; Pang et al., 2021; Niu and Zhu, 2022; Ma et al., 2024). Detailed flowchart of literature search and study selection is shown in Figure 1.

### 3.2 Characteristics of included studies

This analysis included 11 RCTs conducted in Mainland China between 2017 and 2024, enrolling a total of 1,035 participants, of whom 521 received XBJ as adjuvant treatment and 514 received conventional symptomatic therapy, with individual study sample sizes ranging from 66 to 140. The mean participant age varied from 47.67 to 71.03* *years, with a generally balanced gender distribution. All studies had evaluated XBJ as an adjuvant therapy alongside conventional symptomatic treatment, either alone or in combination with antibiotics, for *A. baumannii* infections. XBJ was administered at doses ranging from 50 to 100 mL, with a frequency of one to three times daily, over treatment durations varying from 7 to 30* *days. Nine studies assessed the overall effective rate, five evaluated bacterial clearance rate, and seven reported changes in inflammatory markers such as CRP and PCT. Additionally, five studies analyzed white blood cell count, while only three examined adverse drug events. The general characteristics of the included studies were summarized in Table 1.

**TABLE 1:** Basic characteristics of the included studies.

Author (year)	Country	Design	Sample size		Gender, male		Age, years, mean ± SD		Treatment		Details of XBJ			Outcomes
			C	T	C	T	C	T	C	T	Single dose, *mL*	Frequence, times/day	Duration, days	
Bai et al. (2017)	China	RCT	66	74	39	43	51.50 ± 12.60	48.20 ± 14.20	CT	XBJ + CT	100	2	14	①⑤
Zhang et al. (2017)	China	RCT	50	50	/	/	47.80 ± 2.40	47.80 ± 2.40	CT + CRO	XBJ + CT + CRO	50	2	30	①③
Ma et al., 2018)	China	RCT	46	46	26	28	71.03 ± 4.80	70.12 ± 4.75	CT + TIG+ CFP-Sulb	XBJ + CT + TIG + CFP-Sulb	50	3	14	①②③④⑤⑥
He et al. (2019)	China	RCT	41	41	25	28	49.24 ± 6.52	48.75 ± 6.24	CT + TIG	XBJ + CT + TIG	100	2	7–15	①③④
Liu et al. (2019)	China	RCT	60	60	33	34	48.11 ± 1.39	47.67 ± 1.42	CT + CRO	XBJ + CT + CRO	50	2	30	①③
Dong (2019)	China	RCT	38	38	20	21	49.27 ± 9.72	48.11 ± 10.36	CT + CFP-Sulb	XBJ + CT + CFP-Sulb	50	2	7	②⑥
Liu (2020)	China	RCT	50	50	30	29	49.40 ± 4.20	49.20 ± 4.10	CT + TIG	XBJ + CT + TIG	100	2	14	①
Ma et al. (2020)	China	RCT	33	33	22	20	64.23 ± 8.28	64.30 ± 8.32	CT + TIG	XBJ + CT + TIG	50	2	14	①②③④⑤
Pang et al. (2021)	China	RCT	50	50	34	30	49.80 ± 5.20	51.30 ± 4.70	CT + TIG+ CFP-Sulb	XBJ + CT + TIG + CFP-Sulb	100	1	14	①②③
Niu and Zhu (2022)	China	RCT	46	45	27	25	69.23 ± 4.72	70.02 ± 4.81	CT + TIG+ CFP-Sulb	XBJ + CT + TIG + CFP-Sulb	50	3	14	②④⑤
Ma et al. (2024)	China	RCT	34	34	20	21	50.51 ± 6.35	50.60 ± 6.41	CT + CZA	XBJ + CT + CZA	50	2	7	①③④⑤⑥

RCT, randomized controlled trials; C, control group; T, treatment group; SD, standard deviation; XBJ, Xuebijing; CT, conventional symptomatic treatment; CRO, ceftriaxone; TIG, tigecycline; CFP-Sulb, cefoperazone sulbactam; CZA, ceftazidime avibactam sodium; ①, overall effective rate; ②, bacterial clearance rate; ③, C- reactive protein, CRP; ④, procalcitonin, PCT; ⑤, white blood cell counts, WBC; ⑥, adverse drug events;/: not mentioned.

### 3.3 Risk of bias assessment

**FIGURE 2:**
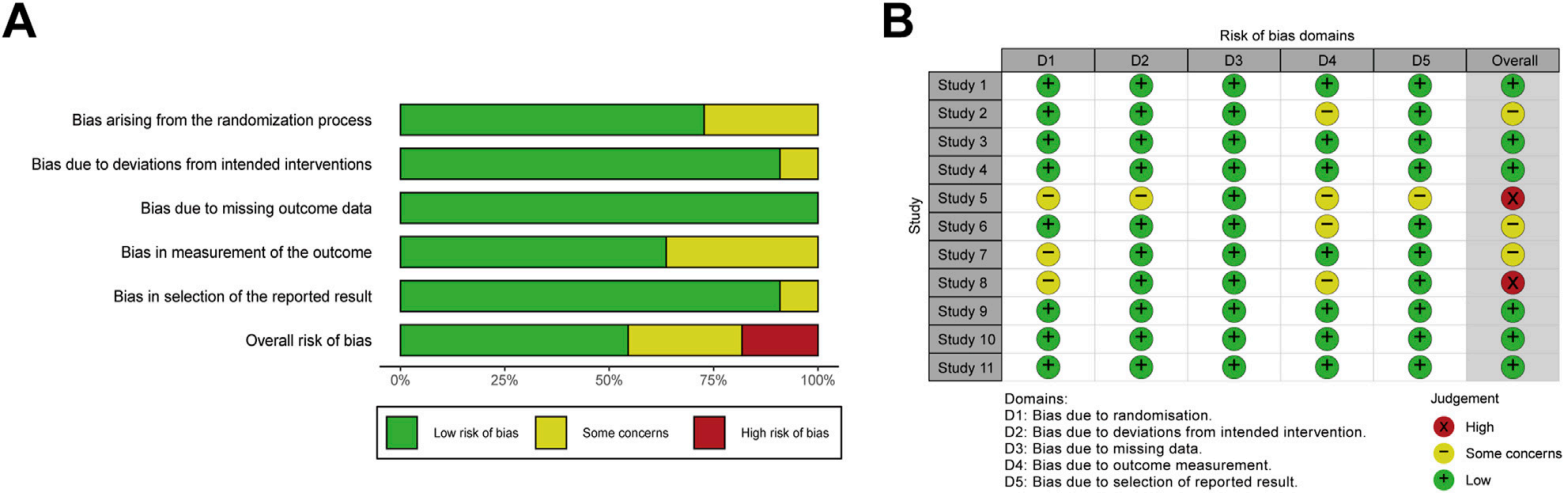
Risk of bias assessment. Weighted bar plots illustrates the distribution of risk-of-bias judgments within each bias domain for **(A)** RCTs, respectively. Traffic light plots indicates the domain-level judgments for each individual result of **(B)** RCTs, respectively. RoB 2 tool was used for RCTs.

Although all included studies reported randomization, only eight trials (Ma et al., 2018; Dong, 2019; He et al., 2019; Liu et al., 2019; Liu, 2020; Ma et al., 2020; Pang et al., 2021; Ma et al., 2024) explicitly described the randomization method. In contrast, the remaining studies lacked clarity in random sequence generation, which raised concerns regarding allocation concealment (Bai et al., 2017; Zhang et al., 2017; Niu and Zhu, 2022). Regarding deviations from intended interventions, one study (Zhang et al., 2017) was rated as having some concerns due to insufficient methodological detail. Overall, six studies were classified as low risk of bias (Ma et al., 2018; He et al., 2019; Liu et al., 2019; Ma et al., 2020; Pang et al., 2021; Ma et al., 2024), three as having some concerns (Bai et al., 2017; Dong, 2019; Liu, 2020), and two as high risk (Zhang et al., 2017; Niu and Zhu, 2022). A summary of the risk of bias assessment was provided in Figure 2.

### 3.4 Primary outcomes

#### 3.4.1 Overall effective rate

**FIGURE 3:**
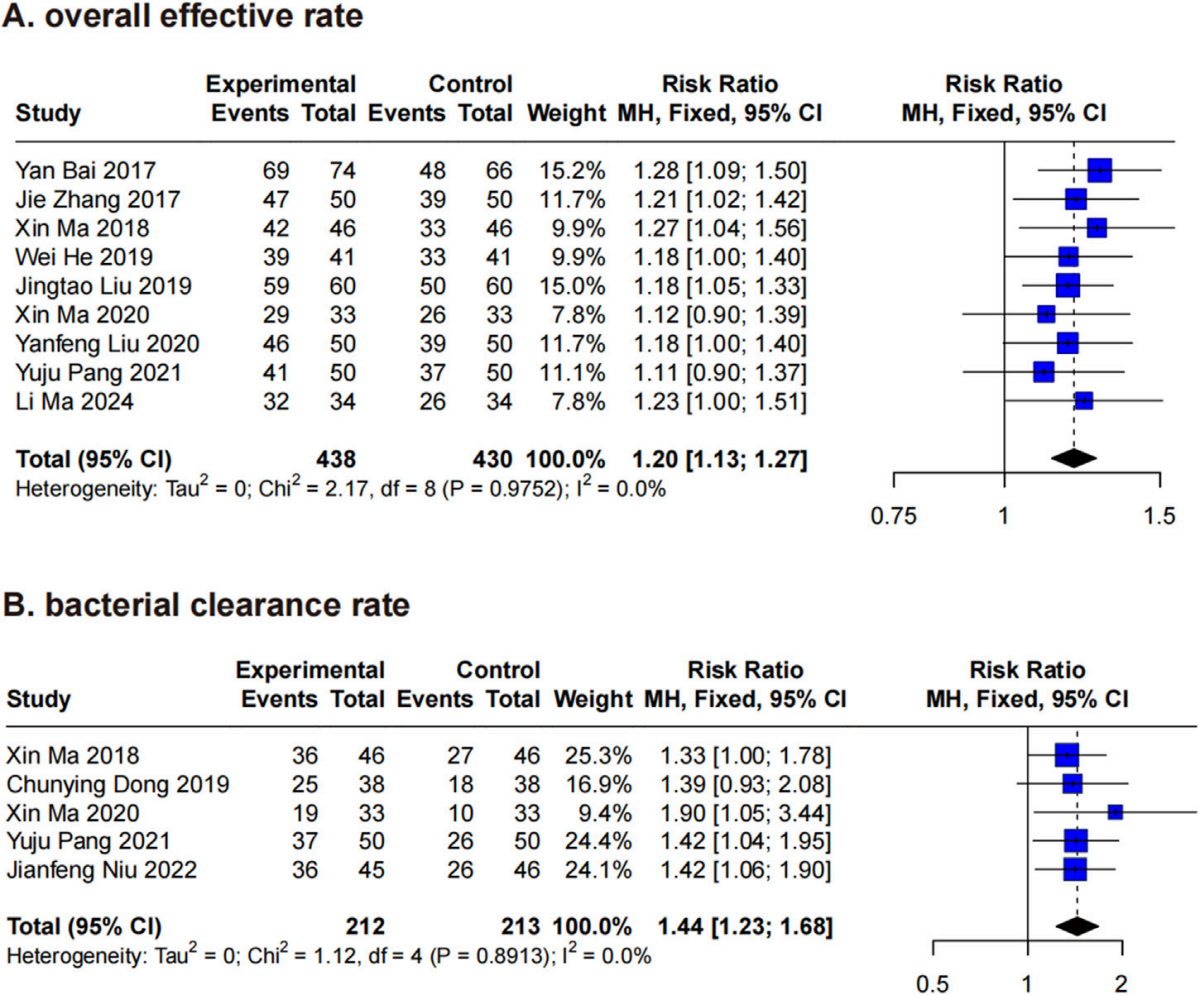
Forest plots for primary outcomes. Forest plots illustrates the effects of XBJ compared to the control group on **(A)** overall effective rate and **(B)** bacterial clearance rate in *A. baumannii* infection.

Nine RCTs (Bai et al., 2017; He et al., 2019; Liu et al., 2019; Liu, 2020; Ma et al., 2018; Ma et al., 2020; Pang et al., 2021; Zhang et al., 2017; Ma et al., 2024) evaluated the overall effective rate of XBJ in the treatment of *A. baumannii* infections. Using a fixed-effects model, the analysis yielded an RR of 1.20 (95% CI: 1.13–1.27), with no significant heterogeneity detected (*P* = 0.98, I^2^ = 0%) (Figure 3A). Consequently, leave-one-out sensitivity analysis was deemed unnecessary. To explore potential factors influencing the overall effective rate of XBJ, subgroup analyses were conducted based on disease type and key application parameters, including single dose, frequency, daily dosage, treatment duration, and total dosage. The results indicated that in *A. baumannii*-related pneumonia, the RR for the XBJ group compared to the control was 1.20 (95% CI: 1.12–1.29), while in *A. baumannii*-related pyelonephritis, the RR was 1.19 (95% CI: 1.08–1.31). No significant difference was observed between these disease types (Chi^2^ = 0.02, *P* = 0.89), and heterogeneity within each subgroup remained negligible (*P* > 0.05, I^2^ = 0% for both) (Supplementary Material 2). Additionally, no significant differences were detected across the various application characteristics of XBJ (*P* > 0.05 for all) (Supplementary Material 2).

#### 3.4.2 Bacterial clearance rate

Five RCTs (Ma et al., 2018; Dong, 2019; Ma et al., 2020; Pang et al., 2021; Niu and Zhu, 2022) assessed the bacterial clearance rate of XBJ in *A. baumannii* infections, with a particular focus on *A. baumannii*-related pneumonia. Using a fixed-effects model, XBJ demonstrated a significant RR of 1.44 (95% CI: 1.23–1.68) compared to controls, with no observed heterogeneity (*P* = 0.89, I^2^ = 0%) (Figure 3B). Given the absence of significant heterogeneity and the limited number of included studies (<6), sensitivity analysis and subgroup analysis were not conducted.

### 3.5 Secondary outcomes

#### 3.5.1 CRP

**FIGURE 4:**
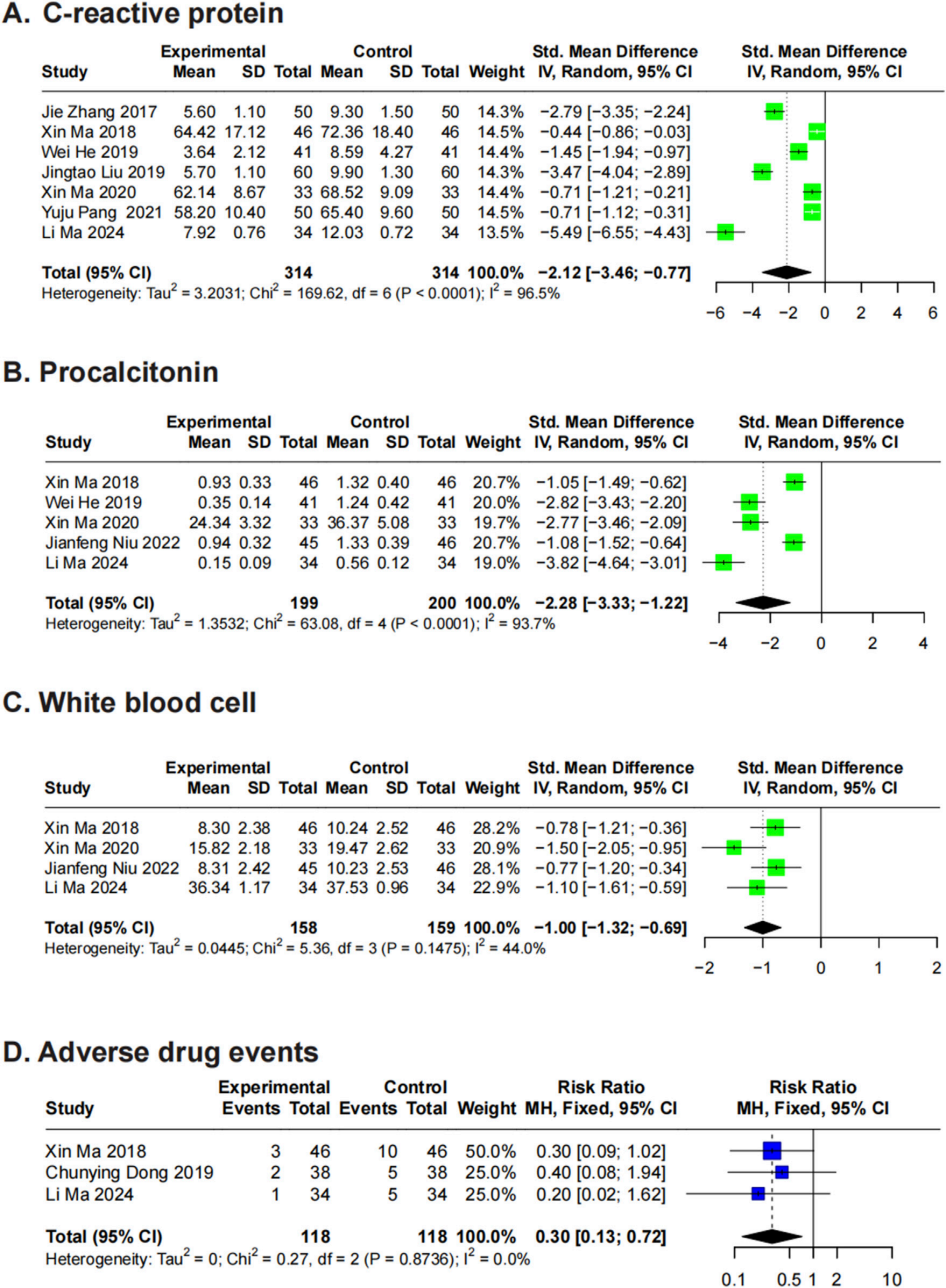
Forest plots for secondary outcomes. Forest plots illustrates the effects of XBJ compared to the control group on **(A)** C-reactive protein, **(B)** procalcitonin, **(C)** white blood cell and **(D)** adverse drug events in *A. baumannii* infection.

Seven RCTs (Zhang et al., 2017; Ma et al., 2018; He et al., 2019; Liu et al., 2019; Ma et al., 2020; Pang et al., 2021; Ma et al., 2024) evaluated CRP levels in patients with *A. baumannii* infections receiving XBJ treatment. Using a random-effects model, the meta-analysis yielded an SMD of −2.12 (95% CI: -3.46 to −0.77) compared to controls, indicating substantial heterogeneity (*P* < 0.01, I^2^ = 96.5%) (Figure 4A). Sensitivity analysis did not identify any single study as the primary source of heterogeneity. Further subgroup analyses showed significant findings for disease type, frequency, treatment duration, and total dosage, whereas no significant differences were observed for single dose and daily dosage (Supplementary Material 3).

#### 3.5.2 PCT

Five RCTs (Ma et al., 2018; He et al., 2019; Ma et al., 2020; Niu and Zhu, 2022; Ma et al., 2024) assessed PCT levels in patients with *A. baumannii* infections treated with XBJ. Using a random-effects model, the analysis yielded an SMD of −2.28 (95% CI: -3.33 to −1.22) compared to controls, indicating significant heterogeneity (*P* < 0.01, I^2^ = 93.7%) (Figure 4B). Sensitivity analysis did not identify any single study as the primary source of heterogeneity (Supplementary Material 4). Given the limited number of included studies, subgroup analysis was not conducted.

#### 3.5.3 WBC counts (×10^9^/L)

Five RCTs (Bai et al., 2017; Ma et al., 2018; Ma et al., 2020; Niu and Zhu, 2022; Ma et al., 2024) evaluated the impact of XBJ on WBC counts in patients with *A. baumannii*-related pneumonia. Meta-analysis using a random-effects model yielded an SMD of -1.13 (95% CI: −1.47 to −0.79) compared to controls, with substantial heterogeneity (*P* = 0.02, I^2^ = 65.6%) (Supplementary Material 5). Sensitivity analysis identified the study by Bai et al. (2017) as a major source of heterogeneity, likely due to its disproportionately large effect size. After excluding this study, the revised meta-analysis using a random-effects model produced an SMD of -1.00 (95% CI: −1.32 to −0.69), with reduced but persistent heterogeneity (*P* = 0.15, I^2^ = 44%). Given the limited number of studies, further subgroup analysis was not performed.

#### 3.5.4 Adverse drug events

Three RCTs (Ma et al., 2018; Dong, 2019; Ma et al., 2024) reported adverse drug events associated with XBJ treatment in patients with *A. baumannii* infections. Using a fixed-effects model, the meta-analysis yielded an RR of 0.30 (95% CI: 0.13–0.72) compared to controls, with no observed heterogeneity (*P* = 0.87, I^2^ = 0%) (Figure 4D). Among the included studies, one (Ma et al., 2018) reported 4 cases of nausea (8.70%), 2 cases each of vomiting (4.35%), rash (4.35%), and thrombocytopenia (4.35%) in the XBJ treatment group. Another (Dong, 2019) documented 1 case each of diarrhea (2.63%) and skin itching (2.63%). The third study (Ma et al., 2024) reported 5 cases (14.71%) of vomiting, diarrhea, or skin itching.

### 3.6 Publication bias

**FIGURE 5:**
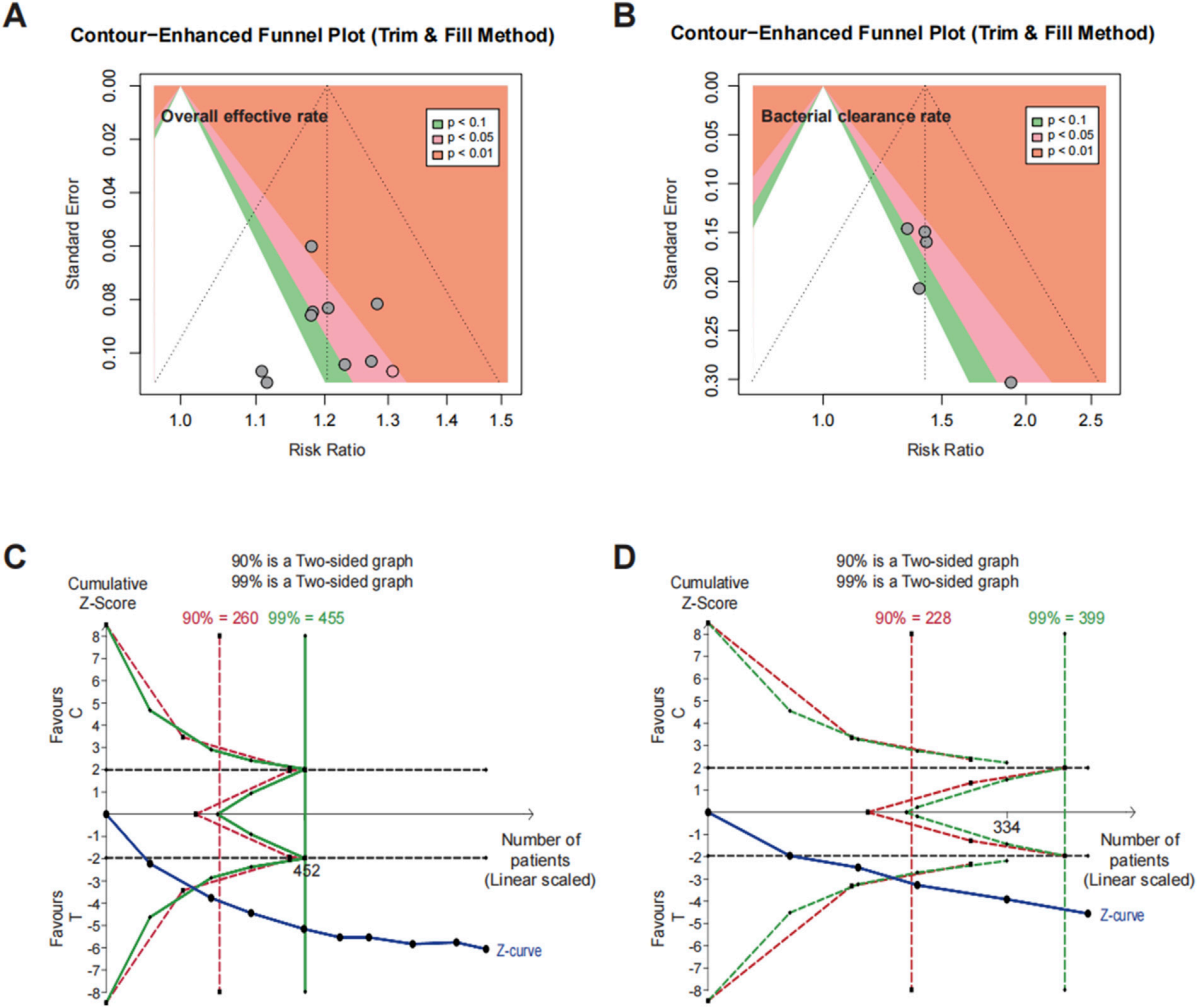
Publication bias and trial sequential analysis. Trim-and-fill funnel plots for **(A)** overall effective rate and **(B)** bacterial clearance rate. Trial sequential analysis for **(C)** overall effective rate and **(D)** bacterial clearance rate.

Egger’s test for funnel plot asymmetry and the trim-and-fill method were employed to assess publication bias for the primary outcomes, including overall effective rate and bacterial clearance rate. For overall effective rate, Egger’s test indicated no significant publication bias (t = −0.26, df = 7, P = 0.8048), with a bias estimate of −0.2458 (SE = 0.9575), suggesting minimal asymmetry. For bacterial clearance rate, some evidence of asymmetry was observed (t = 2.87, df = 3, P = 0.0643), with a bias estimate of 1.7239 (SE = 0.6016), indicating a potential bias toward positive results. However, the further trim-and-fill method did not identify any missing studies, suggesting no need for adjustments in the funnel plots for overall effective rate (Figure 5A) or bacterial clearance rate (Figure 5B).

### 3.7 Trial sequential analysis

In our analysis, TSA was conducted to evaluate cumulative evidence and assess whether the available data provided sufficient certainty to draw reliable conclusions regarding the intervention’s efficacy. In the TSA for overall effective rate (Figure 5C) and bacterial clearance rate (Figure 5D), the cumulative Z-score line crossed the benefit boundary and met the required sample size for both outcomes, as indicated in the 90% (red) and 99% (green) analyses.

### 3.8 GRADE evidence quality assessment

The GRADE evaluation revealed varying levels of certainty across key outcomes (Table 2). The overall effective rate (nine RCTs) was rated high certainty, indicating a significant improvement with XBJ compared to conventional treatment (RR 1.20, 95% CI: 1.13–1.27), despite concerns over potential bias. Similarly, the bacterial clearance rate (five RCTs) was supported by high-certainty evidence (RR 1.44, 95% CI: 1.23–1.68), reflecting a robust and consistent therapeutic benefit. In contrast, the certainty of evidence for inflammatory markers was considerably lower. CRP (seven RCTs) and WBC count (four RCTs) were rated low certainty, while PCT (five RCTs) was rated as very low-certainty evidence, primarily due to serious risk of bias, inconsistency, and suspected publication bias. For adverse drug events (three RCTs), XBJ significantly reduced incidence (RR 0.30, 95% CI: 0.13–0.72), though the certainty of evidence remained moderate to high, constrained by a small sample size.

**TABLE 2:** GRADE evidence quality assessment.

Certainty assessment							No of patients		Effect		Certainty	Importance
No of studies	Study design	Risk of bias	Inconsistency	Indirectness	Imprecision	Other considerations	Xuebijing injection	Normal treatment	Relative (95% CI)	Absolute (95% CI)		
Overall effective rate (follow-up: range 1 week to 4 weeks; assessed with: RR)												
9	randomised trials	serious^a^	not serious	not serious	not serious	dose response gradient	404/438 (92.2%)	331/430 (77.0%)	RR 1.20 (1.13–1.27)	154 more per 1,000 (from 100 more to 208 more)	⨁⨁⨁⨁ High^a^	CRITICAL
bacterial clearance rate (follow-up: range 1 week to 2 weeks; assessed with: RR)												
5	randomised trials	serious^a^	not serious	not serious	not serious	dose response gradient	153/212 (72.2%)	107/213 (50.2%)	RR 1.44 (1.23–1.68)	221 more per 1,000 (from 116 more to 342 more)	⨁⨁⨁⨁ High^a^	IMPORTANT
C reactive protein (follow-up: range 1 week to 4 weeks; assessed with: SMD)												
7	randomised trials	serious^a^	very serious^b^	not serious	not serious	dose response gradient	314	314	-	SMD 2.12 SD lower (3.46 lower to 0.77 lower)	⨁⨁◯◯ Low^a^ ^,^ ^b^	IMPORTANT
Procalcitonin (follow-up: range 1 week to 2 weeks; assessed with: SMD)												
5	randomised trials	serious^a^	very serious^b^	not serious	not serious	publication bias strongly suspected dose response gradient^c^	199	200	-	SMD 2.28 SD lower (3.33 lower to 1.22 lower)	⨁◯◯◯ Very low^a^ ^,^ ^b^ ^,^ ^c^	NOT IMPORTANT
White blood cell count (follow-up: range 1 week to 2 weeks; assessed with: SMD)												
4	randomised trials	very serious^a^	serious^b^	not serious	not serious	dose response gradient	158	159	-	SMD 1 SD lowe**r** (1.32 lower to 0.69 lower)	⨁⨁◯◯ Low^a^ ^,^ ^b^	IMPORTANT
Adverse drug events (follow-up: range 1 week to 2 weeks; assessed with: RR)												
3	randomised trials	serious^a^	not serious	not serious	serious^d^	strong association dose response gradient	6/118 (5.1%)	20/118 (16.9%)	RR 0.30 (0.13–0.72)	119 fewer per 1,000 (from 147 fewer to 47 fewer)	⨁⨁⨁⨁ High^a^ ^,^ ^d^	IMPORTANT

CI, confidence interval; RR, risk ratio; SMD, standardised mean difference.

Explanations.

a The absence of a study protocol poses a risk of selective reporting.

b CI, range is narrow or no overlap, and I2 value is large.

c High risk of publication bias.

d Insufficient sample size.

### 3.9 Network pharmacology

**FIGURE 6:**
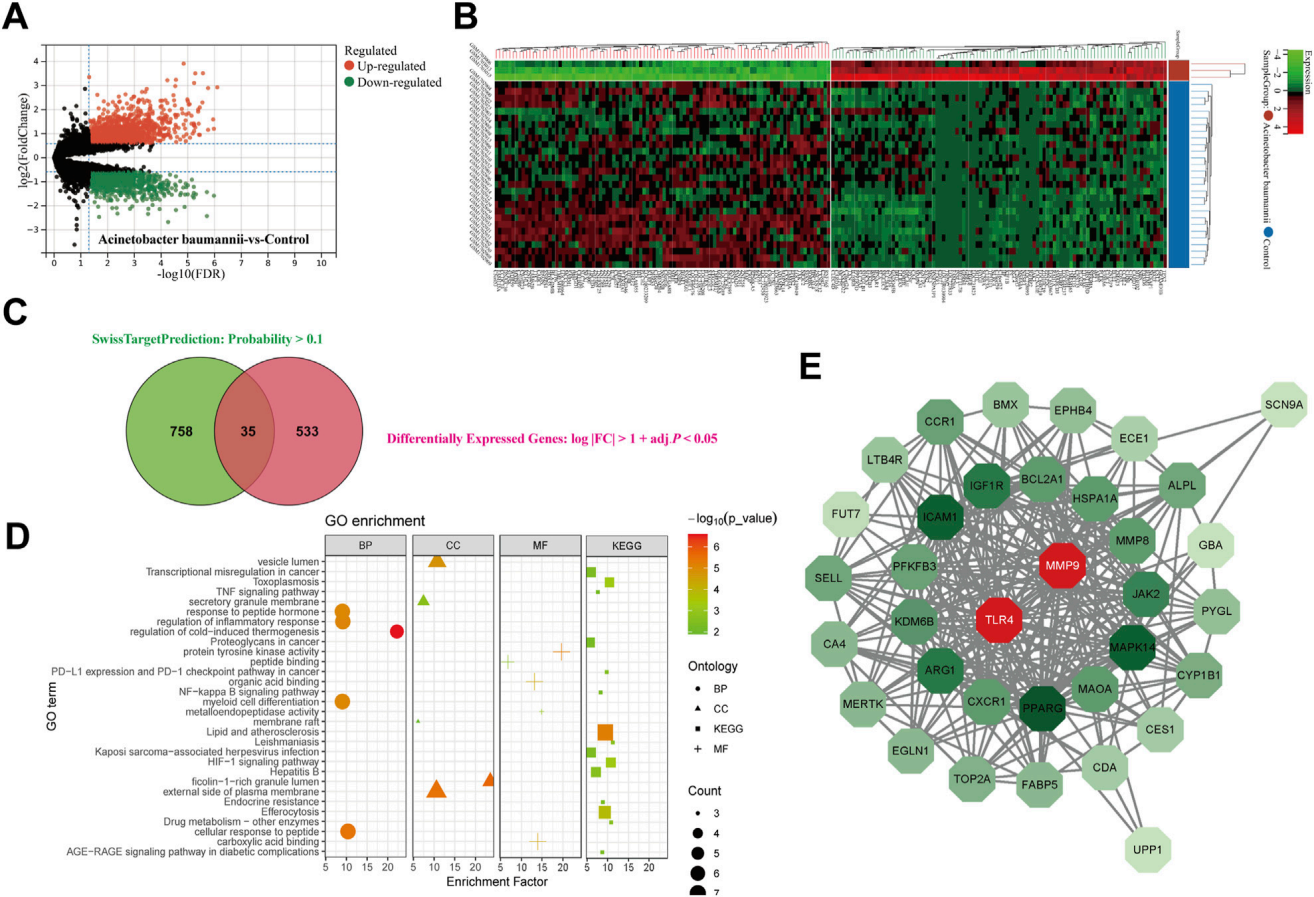
Network pharmacology. The **(A)** volcano plot and **(B)** heatmap for investigating differentially expressed genes in GSE69528. The **(C)** venn plot shows the consensus genes between SwissTargetPrediction and differentially expressed genes. **(D)** GO and KEGG enrichment analysis for consensus genes. **(E)** Protein-protein interaction network analysis of consensus genes revealed two key genes.

A comprehensive analysis was conducted on 145 active ingredients of XBJ, utilizing SwissTargetPrediction to predict their target proteins. A stringent filtering process, which applied a probability threshold of >0.1, identified 758 potential target proteins of XBJ ingredients. Differentially expressed gene (DEG) analysis of the GSE69528 dataset revealed 533 genes upregulated in the *A. baumannii* infection group compared to the control group (Figures 6A,B). The intersection of these datasets yielded 35 common target proteins (Figure 6C).

Functional enrichment analyses, including Gene Ontology (GO) and Kyoto Encyclopedia of Genes and Genomes (KEGG) pathway analyses, revealed associations with key inflammation-related pathways such as Tumor Necrosis Factor-alpha (TNF-α) signaling pathway, Nuclear Factor Kappa B (NF-κB) signaling pathway, and Hypoxia-Inducible Factor 1 (HIF-1) signaling pathway, as well as pathways involved in infectious diseases, including Leishmaniasis, Hepatitis B, and Kaposi sarcoma-associated herpesvirus infection (Figure 6D). To further explore molecular interactions, a protein-protein interaction (PPI) network was constructed using the STRING database and visualized in Cytoscape. Network analysis ranked Matrix Metalloproteinase 9 (MMP9) and TLR4 as the top two key targets based on degree centrality (Figure 6E).

### 3.10 Molecular docking

**FIGURE 7:**
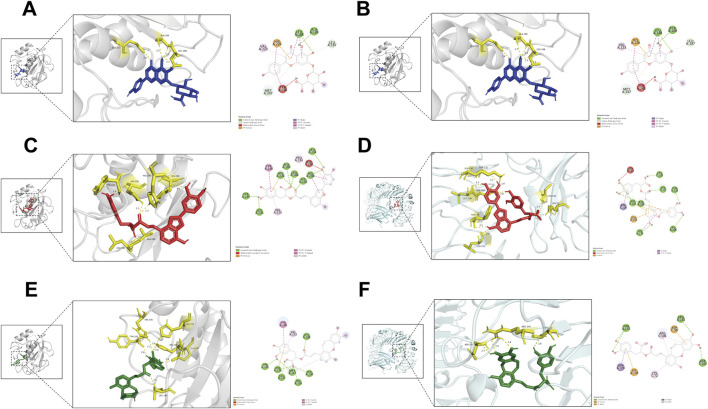
Molecular docking. 3D (left) and 2D (right) docking results for **(A)** scutellarin and MMP9, **(B)** scutellarin and TLR4, **(C)** salvianolic acid C and MMP9, **(D)** salvianolic acid C and TLR4, **(E)** isosalvianolic acid C and MMP9, **(F)** isosalvianolic acid C and TLR4.

AutoDock Vina was employed for batch molecular docking between the 145 active ingredients of XBJ and the active pocket regions of MMP9 and TLR4. The results identified three compounds—scutellarin (labeled in blue), salvianolic acid C (labeled in red), and isosalvianolic acid C (labeled in green)—as potential candidates for simultaneous binding to the active pockets of both MMP9 (labeled in gray) and TLR4 (labeled in cyan). Each of these compounds formed multiple stable hydrogen bonds with MMP9 and TLR4 (Figure 7, with hydrogen bonds shown in yellow in the 3D view and green in the 2D view). Notably, their binding affinities to both proteins were relatively low, suggesting that, despite their multitargeting properties, they may exhibit efficient interaction capabilities.

### 3.11 Molecular dynamics stimulation

**FIGURE 8:**
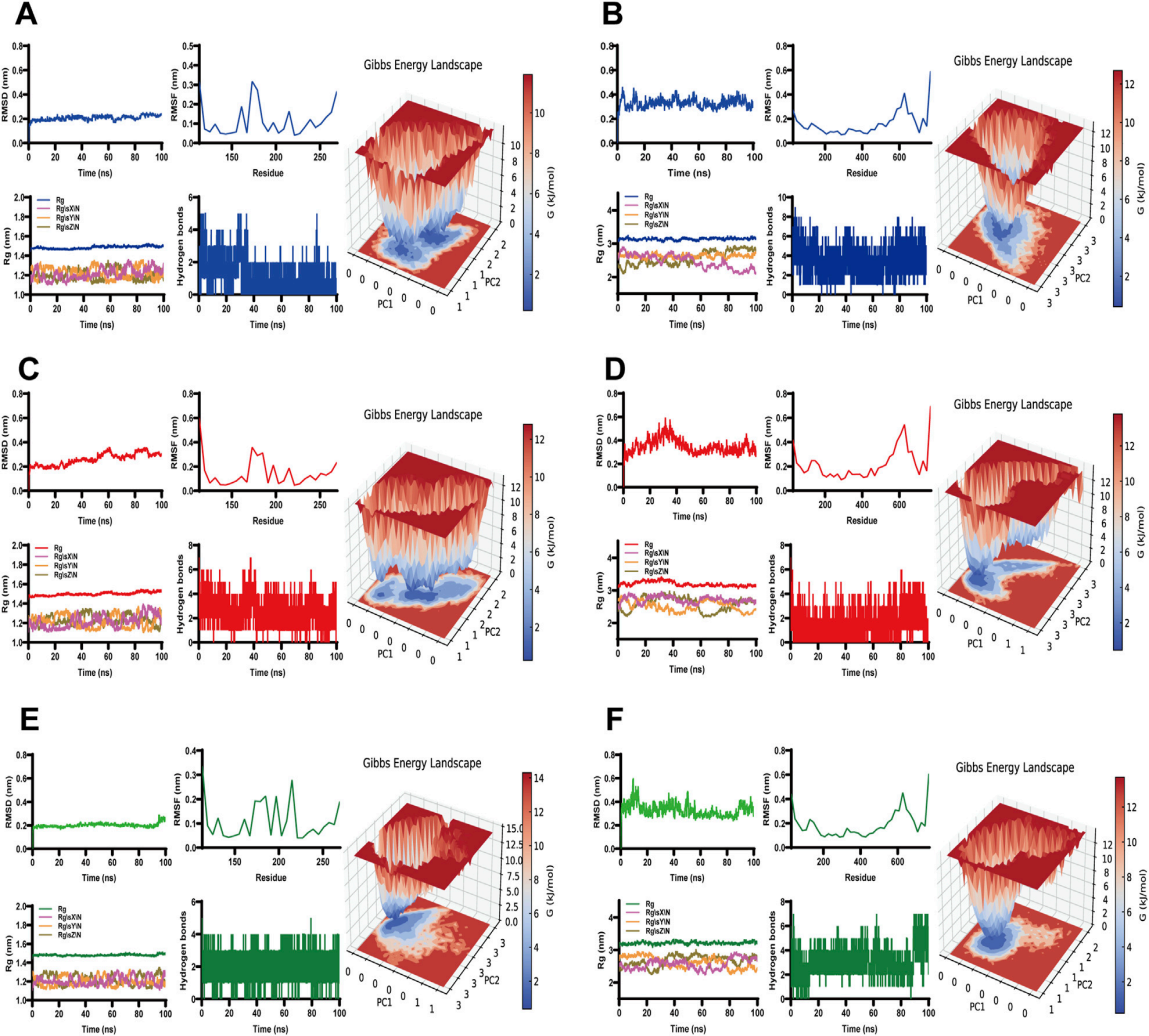
Molecular dynamics simulations. RMSD (top left), RMSF (top middle), gyrate (bottom left), number of H-bonds (bottom middle) and 3D free energy landscape (right) for **(A)** scutellarin and MMP9, **(B)** scutellarin and TLR4, **(C)** salvianolic acid C and MMP9, **(D)** salvianolic acid C and TLR4, **(E)** isosalvianolic acid C and MMP9, **(F)** isosalvianolic acid C and TLR4.

GROMACS was employed to perform molecular dynamics simulations, and to investigate the binding interactions of three small-molecule ligands—scutellarin (labeled in blue), salvianolic acid C (labeled in red), and isosalvianolic acid C (labeled in green)—with MMP9 (labeled in gray) and TLR4 (labeled in cyan) proteins individually. The simulation results demonstrated that these ligand-protein complexes maintained stable binding conformations within the protein binding pockets. Key parameters, including Root Mean Square Deviation (RMSD), Root Mean Square Fluctuation (RMSF), Radius of Gyration (GYRATE), and hydrogen bond analysis, were assessed to evaluate stability and interaction dynamics. RMSD analysis indicated that the protein-ligand complexes reached equilibrium and remained stable throughout the simulation. RMSF results showed moderate constraints on protein flexibility, contributing to changes in overall conformational compactness, which was further supported by GYRATE data reflecting structural alterations. Additionally, hydrogen bond analysis confirmed stable interactions between the ligands and proteins, reinforced by the presence of stable interaction energy minima (Figure 8). These findings provided valuable insights into the binding mechanisms of these compounds, and offered a foundation for further exploration of their biological functions.

## 4 Discussion

In recent years, *A. baumannii* infections have posed a significant challenge in healthcare settings, affecting multiple anatomical sites, including the respiratory tract, bloodstream, skin, soft tissue, urinary tract, and central nervous system (Cavallo et al., 2023; Diao et al., 2024). Notably, *A. baumannii* exhibits remarkable resilience to adverse environmental conditions and a pronounced propensity for resistance across a broad spectrum of antibiotics, establishing itself as a formidable nosocomial pathogen (Shi et al., 2024). The inappropriate use of antibiotics, cross-infections, and the dissemination of resistance-associated genetic elements have facilitated the emergence of multidrug-resistant (MDR), extensively drug-resistant (XDR), and pan-drug-resistant (PDR) *A. baumannii* strains (Thacharodi et al., 2024). This escalating resistance presents substantial challenges in clinical management, contributing to rising incidence and mortality rates, particularly among critically ill patients (Zhang et al., 2024b). Hence, effective control measures and novel therapeutic strategies are urgently needed to mitigate the impact of *A. baumannii* infections on patient outcomes.

Innate immune signaling serves as the host’s first line of defense against *A. baumannii*, orchestrating an immune response by activating downstream pro-inflammatory cytokines (Shadan et al., 2023). Host immune and epithelial cells detect pathogen-associated molecular patterns (PAMPs) through various pattern recognition receptors (PRRs) (Tiku et al., 2022), among which Toll-like receptor 4 (TLR4) plays a central role in cytokine production. LPS, a major component of the outer membrane of Gram-negative bacteria, is a well-characterized PAMP that activates TLR4 signaling, triggering NF-κB activation and the subsequent production of cytokines such as IL-6, IL-12, and TNF-α (Shadan et al., 2023; Tiku et al., 2022). Additionally, *A. baumannii* secretes a biologically active lipid that activates the Toll-like receptor 2 (TLR2) signaling pathway in human and murine macrophages, leading to the production of pro-inflammatory cytokines, including IL-6, IL-8, and TNF-α (Tiku et al., 2022). Beyond NF-κB signaling, this inflammatory response also involves inflammasome activation, contributing to cell death and exacerbating host tissue damage (Tiku et al., 2022). The excessive immune-inflammatory response induced by *A. baumannii* results in a cytokine storm, severe systemic inflammation, and sepsis-induced organ damage, posing a critical threat to patient survival.

In managing *A. baumannii* infections, combination antibiotic therapy remains a key strategy, aiming to expand antimicrobial coverage while awaiting susceptibility test results, suppress resistance, and improve patient prognosis (Rafailidis et al., 2024). However, despite its widespread use, the clinical efficacy of combination therapy remains inadequately supported by robust evidence, particularly for drug-resistant *A. baumannii* infections (Zhang et al., 2024a). As novel antimicrobial approaches, such as antimicrobial peptides and bacteriophages, are currently under rigorous investigation (Rangel et al., 2023), the challenge of *A. baumannii* resistance to conventional therapeutics continues to intensify. Given the pathogen’s formidable colonization ability and intricate resistance mechanisms, exploring adjunctive therapies alongside conventional antibiotics is essential. Such strategies should not only modulate the host’s inflammatory response to *A. baumannii* infection but also enhance immune function, mitigate inflammatory damage, and minimize organ injury. These efforts are critical for improving clinical outcomes in *A. baumannii* infections, which are often associated with high morbidity and mortality.

As an alternative and adjunctive therapy, traditional Chinese medicine (TCM) is increasingly recognized for its efficacy and safety in managing various diseases. In recent years, studies have highlighted the unique advantages of TCM in enhancing immune function, reducing fever, and alleviating symptoms associated with COVID-19 (Kang et al., 2022). Notably, TCM has gained widespread acceptance in treating inflammatory conditions such as sepsis by modulating inflammatory pathways, regulating immune responses, and mitigating oxidative stress (Song et al., 2023). XBJ, a patented TCM formulation developed by Professor Jinda Wang, originates from the Qing dynasty prescription *Xuefu Zhuyu* decoction (Xie et al., 2019). Possessing antibacterial, antioxidative, and anti-endotoxin properties, XBJ has been approved as a State Category II New Drug for sepsis treatment in China, demonstrating both efficacy and safety in clinical practice (Tian et al., 2021). Notably, the *Efficacy of Xuebijing in Patients With Sepsis* (EXIT-SEP) trial, a double-blind, placebo-controlled study by Liu et al., underscored the potential of XBJ in reducing 28-day mortality among sepsis patients (Liu et al., 2023). Furthermore, XBJ has shown promise in attenuating inflammatory responses and improving patient outcomes during the COVID-19 pandemic (Guo et al., 2020). Based on existing clinical evidence, this meta-analysis demonstrates that XBJ significantly outperforms the control group in overall effective rate and bacterial clearance rate while markedly reducing the expression of key inflammatory markers, including WBC, CRP, and PCT.

Initial findings from the meta-analysis confirmed the efficacy of XBJ in managing *A. baumannii* infections. To further elucidate its underlying therapeutic mechanisms, network pharmacology was employed to identify potential bioactive constituents of XBJ and their corresponding target proteins. Previous network pharmacology studies have primarily relied on databases such as TCMSP; however, limitations in database updates and herbal ingredient diversity have often resulted in repetitive findings, such as the frequent identification of quercetin as a core component (Miao et al., 2022). To enhance data accuracy, this study integrated previously published research utilizing mass spectrometry and other analytical methods to detect XBJ ingredients (Huang et al., 2011; Jiang et al., 2013; Sun et al., 2017; Zuo et al., 2017a; Zuo et al., 2017b; Zuo et al., 2019; Yu et al., 2022; Li et al., 2023). Core *A. baumannii* infection targets were then subjected to batch molecular docking with all known XBJ constituents, and the findings were further validated via molecular dynamics simulations to assess result reliability. Given that MMP9 and TLR4 have been reported as key regulators of inflammatory responses in *A. baumannii* infection (Garcia-Patino et al., 2017; Li et al., 2021), molecular docking and dynamics simulations revealed that scutellarin, salvianolic acid C, and isosalvianolic acid C exhibited strong binding affinities for MMP9 and TLR4, respectively. These findings suggest that specific XBJ ingredients exert targeted effects *in vivo*, inhibiting critical inflammatory pathways induced by *A. baumannii*.

This study also identified a significant enhancement in bacterial clearance rates in *A. baumannii* infections following XBJ administration compared to the control group. XBJ consists of extracts from five traditional Chinese medicinal herbs, some of which have demonstrated antibacterial properties. For instance, the paeoniflorin derivative (PRAE-a) from *Paeonia lactiflora Pall.* aqueous extract (PRAE) exhibits potent antibacterial activity by selectively targeting and inhibiting the α-toxin of *Staphylococcus aureus*, thereby suppressing hemolytic activity and disrupting pore formation (Liu et al., 2021). Similarly, extracts of *Angelica sinensis* (Oliv.) *Diels* extract (AE) and *Sophora flavescens* Aiton extract (SE) have shown significant antibacterial effects against *Escherichia coli*, *Staphylococcus aureus*, and *Shigella Castellani* (Han and Guo, 2012). While these findings offer insight into the antibacterial potential of XBJ, further pharmacological investigations are warranted to substantiate its antibacterial and anti-inflammatory properties.

Building on preliminary investigations into the overall effective rate of XBJ in treating *A. baumannii* infections, this study further explored the optimal dosage and specific clinical application details through detailed subgroup analyses (Supplementary Material 3). Subgroup analysis of single doses indicated comparable effect sizes across different dose levels: 50 mL per dose (RR = 1.20, 95% CI: 1.11–1.30) and 100 mL per dose (RR = 1.20, 95% CI: 1.10–1.31). Notably, the 50 mL subgroup had a narrower 95% confidence interval, suggesting greater reliability. Regarding administration frequency, increased dosing frequency appeared to enhance XBJ efficacy, with once-daily administration yielding an RR of 1.11 (95% CI: 0.90–1.37), twice-daily an RR of 1.20 (95% CI: 1.13–1.28), and three-times-daily an RR of 1.27 (95% CI: 1.04–1.56). However, given that only one original study was available for both the once-daily and three-times-daily subgroups, potential bias should be considered. For daily dosage, the subgroup receiving ≤100 mL per day (RR = 1.17, 95% CI: 1.08–1.26) showed a lower effect than those receiving ≤200 mL per day (RR = 1.23, 95% CI: 1.13–1.35). In terms of treatment duration, the 4-week subgroup (RR = 1.19, 95% CI: 1.08–1.31) exhibited a lower effect than the 1-week (RR = 1.20, 95% CI: 1.06–1.37) and 2-week (RR = 1.20, 95% CI: 1.10–1.31) subgroups, with the 2-week subgroup demonstrating the narrowest confidence interval, indicating greater reliability. Regarding total dosage, the subgroup receiving ≤1,400 mL had an RR of 1.16 (95% CI: 1.05–1.28), those receiving ≤2,800 mL had an RR of 1.25 (95% CI: 1.13–1.38), while those exceeding 2,800 mL had an RR of 1.19 (95% CI: 1.08–1.31), suggesting that a total dosage of ≤2,800 mL may offer the greatest efficacy, with no additional benefit observed beyond this threshold. In summary, for *A. baumannii* infections, the optimal XBJ regimen appears to be 50 mL per dose, administered twice daily for 2 weeks, with a total dosage of 2,800 mL. This regimen demonstrated satisfactory efficacy and aligned with the recommended dosage in the XBJ manufacturer’s guidelines. However, further real-world studies are warranted to validate these findings.

Additionally, an extensive evaluation of XBJ’s safety profile in *A. baumannii* infections revealed a minimal occurrence of adverse drug events. A real-world study involving 31,913 participants across 93 hospitals (Zheng et al., 2019) reported an overall adverse drug events incidence of 0.30% with XBJ treatment, predominantly presenting as mild or non-serious events, with skin lesions being the most common. These findings underscore the relatively favorable safety profile of XBJ in hospitalized patients. To mitigate ADR risks, strict adherence to administration guidelines is essential. This real-world evidence highlights the necessity of judicious XBJ use to optimize safety while leveraging its therapeutic benefits in *A. baumannii* infections.

## 5 Study strengths and limitations

This study employs meta-analysis to synthesize clinical evidence on the efficacy of XBJ in treating *A. baumannii* infections while exploring its therapeutic mechanisms through an integrated approach combining network pharmacology, molecular docking, and molecular dynamics simulations. In the network pharmacology analysis, we independently identified the active ingredients of XBJ, avoiding reliance on traditional Chinese medicine databases such as TCMSP to mitigate potential result overlap and instead leveraging mass spectrometry data from reported studies on XBJ. This strategy provided valuable insights into the pharmacological mechanisms underlying its therapeutic effects in *A. baumannii* infections.

However, several limitations should be acknowledged. First, variations in study quality and methodology—including differences in design, small sample sizes, and potential publication biases—posed challenges in fully assessing XBJ’s efficacy. Second, the complex and diverse composition of XBJ, comprising multiple bioactive ingredients, presented analytical challenges that complicate result interpretation. Moreover, the optimal dosage and regimen of XBJ for *A. baumannii* infections in clinical practice remain uncertain. Based on our findings, we cautiously propose a regimen of 50 mL per dose, administered intravenously twice daily for at least 2 weeks, as both safe and effective, though validation through larger-scale studies is required.

When interpreting these results, careful consideration of these limitations is essential. Although no significant asymmetry or publication bias was observed in the funnel plot analysis, all included studies were conducted in China, suggesting a potential for regional publication bias. Furthermore, despite rigorous quality assessments of the included RCTs based on Cochrane guidelines, the possibility of heterogeneity cannot be entirely excluded. Finally, due to the limited number of studies, many outcome indicators were not subjected to sensitivity or subgroup analysis, which may affect the robustness and depth of our conclusions. Further research is needed to validate and refine these findings.

## 6 Conclusion

This study provides a comprehensive analysis of the clinical efficacy, safety, and mechanisms of action of XBJ in the treatment of *A. baumannii* infections. Meta-analysis demonstrated significant improvements in both overall effective rate and bacterial clearance rate with XBJ treatment, alongside notable reductions in inflammatory markers, including WBC count, CRP, and PCT. Furthermore, network pharmacology, molecular docking, and molecular dynamics simulations elucidated the anti-inflammatory effects of XBJ’s active ingredients, revealing the potential targeted binding of scutellarin, salvianolic acid C, and isosalvianolic acid C to MMP9 and TLR4, respectively. Despite these promising findings, methodological differences across studies and the complex composition of XBJ posed challenges in fully elucidating its therapeutic effects. Further clinical and pharmacological research is warranted to validate and refine its efficacy and mechanisms of action against *A. baumannii* infections, thereby strengthening the evidence base for its clinical application.

## Data Availability

The datasets presented in this study can be found in online repositories. The names of the repository/repositories and accession number(s) can be found in the article/Supplementary Material.

## Glossary

*A. baumannii*: *Acinetobacter baumannii*
XBJ: Xuebijing
MDR: multidrug-resistant
XDR: extensively drug-resistant
PDR: pan-drug-resistant
RR: risk ratio
SMD: standardized mean differences
LPS: lipopolysaccharide
TLR4: Toll-like receptor 4
TNF: Tumor Necrosis Factor
NF-κB: Nuclear Factor Kappa B
HIF-1: Hypoxia-Inducible Factor 1
MMP9: Matrix Metalloproteinase 9
WBC: white blood cell
CRP: C-reactive protein
PCT: procalcitonin
RCT: randomized controlled trial
NRSI: Non-randomized Studies - of Interventions
RoB2: revised tool for risk of bias in randomized trials
TSA: Trial sequential analysis
GO: gene ontology
KEGG: Kyoto Encyclopedia of Genes and Genomes
STRING: Search Tool for the Retrieval of Interacting Genes/Proteins
C: control group
T: treatment group
SD: standard deviation
CT: conventional treatment
CRO: ceftriaxone
TIG: tigecycline
CFP-Sulb: cefoperazone sulbactam
CZA: ceftazidime avibactam sodium
TCM: traditional Chinese medicine
