# Supplementary material for: Therapeutic efficacy and mechanisms of Xuebijing in Acinetobacter baumannii infection based on meta-analysis and integrated pharmacology approaches
Source: Front Pharmacol. 2025 May 23;16:1598359. doi: 10.3389/fphar.2025.1598359 (PMC12141340; doi:10.3389/fphar.2025.1598359)
Supplement: Supplementary file 1 [file DataSheet1.docx]

***Supplementary Materials***

**Table of contents**

**[Supplementary Material 1: Search strategies 1](#_Toc450)**

[Table 1. Search strategy for PubMed database. 1](#_Toc10715)

[Table 2. Search strategy for EBSCO database. 1](#_Toc29166)

[Table 3. Search strategy for EMBASE database. 1](#_Toc19279)

[Table 4. Search strategy for Web of Science database. 1](#_Toc4688)

[Table 5. Search strategy for Cochrane database. 2](#_Toc19472)

[Table 6. Search strategy for China National Knowledge Infrastructure/CNKI database. 2](#_Toc6648)

[Table 7. Search strategy for VIP's Chinese Science and Technology Journal Database/VIP. 2](#_Toc2171)

[Table 8. Search strategy for Wanfang Database. 2](#_Toc5935)

[Table 9. Search strategy for China Biology Medicine/CBM. 3](#_Toc29581)

**[Supplementary Material 2: Subgroup analysis of overall effective rate. 4](#_Toc16241)**

[Supplementary Figure 1. Subgroup analysis of overall effective rate based on disease types (Pneumonia vs. Pyelonephritis). 4](#_Toc2431)

[Supplementary Figure 2. Subgroup analysis of overall effective rate based on single dose of XBJ (≤ 50 mL/dose vs. ≤ 100 mL/dose). 4](#_Toc28693)

[Supplementary Figure 3. Subgroup analysis of overall effective rate based on frequency of XBJ (Once daily vs. Twice daily vs. Thrice daily). 5](#_Toc17474)

[Supplementary Figure 4. Subgroup analysis of overall effective rate based on daily dosage of XBJ (≤ 100 mL/day vs. ≤ 200 mL/day). 5](#_Toc27994)

[Supplementary Figure 5. Subgroup analysis of overall effective rate based on treatment duration of XBJ (≤ 1 week vs. ≤ 2 weeks vs. ≤ 4 weeks). 6](#_Toc2279)

[Supplementary Figure 6. Subgroup analysis of overall effective rate based on total dosage of XBJ (≤ 1400 mL vs. ≤ 2800 mL vs. > 2800 mL). 6](#_Toc6833)

**[Supplementary Material 3: Sensitivity and subgroup analyses of CRP. 7](#_Toc32051)**

[Supplementary Figure 1. Sensitivity analysis using baujat plot of CRP. 7](#_Toc32377)

[Supplementary Figure 2. Sensitivity analysis using leave-one-out method of CRP. 7](#_Toc28404)

[Supplementary Figure 3. Subgroup analysis of CRP based on disease types (Pneumonia vs. Pyelonephritis). 8](#_Toc13455)

[Supplementary Figure 4. Subgroup analysis of CRP based on single dose of XBJ (≤ 50 mL/dose vs. ≤ 100 mL/dose). 8](#_Toc716)

[Supplementary Figure 5. Subgroup analysis of CRP based on frequency of XBJ (Once daily vs. Twice daily vs. Thrice daily). 9](#_Toc20057)

[Supplementary Figure 6. Subgroup analysis of CRP based on daily dosage of XBJ (≤ 100 mL/day vs. ≤ 200 mL/day). 9](#_Toc20114)

[Supplementary Figure 7. Subgroup analysis of CRP based on treatment duration of XBJ (≤ 1 week vs. ≤ 2 weeks vs. ≤ 4 weeks). 10](#_Toc21563)

[Supplementary Figure 8. Subgroup analysis of CRP based on total dosage of XBJ (≤ 1400 mL vs. ≤ 2800 mL vs. > 2800 mL). 10](#_Toc25461)

**[Supplementary Material 4: Sensitivity analyses of PCT. 11](#_Toc31634)**

[Supplementary Figure 1. Sensitivity analysis using baujat plot of PCT. 11](#_Toc11914)

[Supplementary Figure 2. Sensitivity analysis using leave-one-out method of PCT. 11](#_Toc32169)

**[Supplementary Material 5: Sensitivity analyses of WBC. 12](#_Toc2663)**

[Supplementary Figure 1. The original forest plot, generated using a random-effects model, reveals significant heterogeneity across the included studies. 12](#_Toc8010)

[Supplementary Figure 2. Sensitivity analysis using baujat plot of WBC. (](#_Toc29433)*[red spot represents outlier study](#_Toc29433)*[) 12](#_Toc29433)

[Supplementary Figure 3. Sensitivity analysis using leave-one-out method of WBC. (](#_Toc13780)*[red line represents outlier study](#_Toc13780)*[) 12](#_Toc13780)

**[Supplementary Material 6: Active ingredients of XBJ (145 ingredients). 13](#_Toc5310)**

**[Supplementary Material 7: Targets of XBJ ingredients predicted from SwissTargetPrediction website (758 targets with probability > 0.1). 15](#_Toc7676)**

**[Supplementary Material 8: Up-regulated genes in](#_Toc31489) *[A. baumannii](#_Toc31489)* [group from DEG analysis of GSE69528 (533 up-regulated genes with |log FC| > 1](#_Toc31489) *[plus](#_Toc31489)* [adj.P.Val < 0.05). 21](#_Toc31489)**

**[Supplementary Material 9: Nodes degrees in PPI network construction (sorted in descending order by degree). 35](#_Toc31142)**

**[Supplementary Material 10: Binding affinities in molecular docking analysis. 36](#_Toc31951)**

**Supplementary Material 1:** Search strategies

Table 1. Search strategy for PubMed database.

| **ID** | **Search** | **Hits** |
| --- | --- | --- |
| #1 | "Acinetobacter baumannii"[Mesh] Sort by: Most Recent | 8091 |
| #2 | acinetobacter baumannii[Title/Abstract] OR A. baumannii[Title/Abstract] OR acinetobacter[Title/Abstract] OR baumannii[Title/Abstract] | 26796 |
| #3 | xuebijing[Title/Abstract] OR XBJ[Title/Abstract] | 335 |
| #4 | #1 OR #2 | 27171 |
| #5 | #3 AND #4 | 2 |
| **Last Run Date: 09/02/2025** | | |

Table 2. Search strategy for EBSCO database.

| **ID** | **Search** | **Hits** |
| --- | --- | --- |
| S1 | SU acinetobacter baumannii | 23804 |
| S2 | TI ( acinetobacter baumannii OR A. baumannii OR acinetobacter OR baumannii ) OR AB ( acinetobacter baumannii OR A. baumannii OR acinetobacter OR baumannii ) | 74673 |
| S3 | SU ( xuebijing OR XBJ ) OR TI ( xuebijing OR XBJ ) OR AB ( xuebijing OR XBJ ) | 858 |
| S4 | S1 OR S2 | 75809 |
| S5 | S3 AND S4 | 2 |
| **Last Run Date: 09/02/2025** | | |

Table 3. Search strategy for EMBASE database.

| **ID** | **Search** | **Hits** |
| --- | --- | --- |
| #1 | 'acinetobacter baumannii'/exp | 27511 |
| #2 | 'acinetobacter baumannii':ab,kw,ti | 17861 |
| #3 | 'a. baumannii':ab,kw,ti OR 'acinetobacter':ab,kw,ti OR 'baumannii':ab,kw,ti | 34816 |
| #4 | 'xuebijing':ab,kw,ti OR 'xbj':ab,kw,ti | 443 |
| #5 | #1 OR #2 OR #3 | 44150 |
| #6 | #4 AND #5 | 2 |
| **Last Run Date: 09/02/2025** | | |

Table 4. Search strategy for Web of Science database.

| **ID** | **Search** | **Hits** |
| --- | --- | --- |
| #1 | TS=(acinetobacter baumannii) | 28810 |
| #2 | ((TI=(acinetobacter baumannii OR A. Baumannii OR acinetobacter OR baumannii)) OR AB=(acinetobacter baumannii OR A. Baumannii OR acinetobacter OR baumannii)) OR TS=(acinetobacter baumannii OR A. Baumannii OR acinetobacter OR baumannii) | 51358 |
| #3 | ((TS=(xuebijing OR XBJ)) OR TI=(xuebijing OR XBJ)) OR AB=(xuebijing OR XBJ) | 813 |
| #4 | #1 OR #2 | 51358 |
| #5 | #3 AND #4 | 3 |
| **Last Run Date: 09/02/2025** | | |

Table 5. Search strategy for Cochrane database.

| **ID** | **Search** | **Hits** |
| --- | --- | --- |
| #1 | MeSH descriptor: [Acinetobacter baumannii] explode all trees | 42 |
| #2 | (acinetobacter baumannii OR A. baumannii OR acinetobacter OR baumannii):ti,ab,kw | 600 |
| #3 | (xuebijing OR XBJ):ti,ab,kw | 116 |
| #4 | #1 OR #2 | 600 |
| #5 | #3 AND #4 | 0 |
| **Last Run Date: 09/02/2025** | | |

Table 6. Search strategy for China National Knowledge Infrastructure/CNKI database.

| **ID** | **Search** | **Hits** |
| --- | --- | --- |
| #1 | (SU%='Acinetobacter baumannii'+'A. baumannii') OR (TKA='Acinetobacter baumannii'+'A. baumannii') OR (KY='Acinetobacter baumannii'+'A. baumannii') OR (TI='Acinetobacter baumannii'+'A. baumannii') OR (FT='Acinetobacter baumannii'+'A. baumannii') | 128011 |
| #2 | (SU%='xuebijing'+'XBJ') OR (TKA='xuebijing'+'XBJ') OR (KY='xuebijing'+'XBJ') OR (TI='xuebijing'+'XBJ') OR (FT='xuebijing'+'XBJ') | 15682 |
| #3 | #1 AND #2 | 553 |
| **Last Run Date: 09/02/2025** | | |

Table 7. Search strategy for VIP's Chinese Science and Technology Journal Database/VIP.

| **ID** | **Search** | **Hits** |
| --- | --- | --- |
| #1 | (M="Acinetobacter baumannii" OR "A. baumannii") OR (K="Acinetobacter baumannii" OR "A. baumannii") OR (T="Acinetobacter baumannii" OR "A. baumannii") OR (R="Acinetobacter baumannii" OR "A. baumannii") OR (U="Acinetobacter baumannii" OR "A. baumannii") | 19765 |
| #2 | (M="xuebijing" OR "XBJ") OR (K="xuebijing" OR "XBJ") OR (T="xuebijing" OR "XBJ") OR (R="xuebijing" OR "XBJ") OR (U="xuebijing" OR "XBJ") | 3863 |
| #3 | #1 AND #2 | 12 |
| **Last Run Date: 09/02/2025** | | |

Table 8. Search strategy for Wanfang Database.

| **ID** | **Search** | **Hits** |
| --- | --- | --- |
| #1 | Subject:("Acinetobacter baumannii" OR "A. baumannii") OR Title OR Keywords:("Acinetobacter baumannii" OR "A. baumannii") OR Abstract:("Acinetobacter baumannii" OR "A. baumannii") OR Full Text:("Acinetobacter baumannii" OR "A. baumannii") | 25201 |
| #2 | Subject:("xuebijing" OR "XBJ") OR Title OR Keywords:("xuebijing" OR "XBJ") OR Abstract:("xuebijing" OR "XBJ") OR Full Text:("xuebijing" OR "XBJ") | 4567 |
| #3 | #1 AND #2 | 16 |
| **Last Run Date: 09/02/2025** | | |

Table 9. Search strategy for China Biology Medicine/CBM.

| **ID** | **Search** | **Hits** |
| --- | --- | --- |
| #1 | (( "Acinetobacter baumannii"[Full Text: Intelligent] OR "A. baumannii"[Full Text: Intelligent]) OR "Acinetobacter baumannii OR A. baumannii"[Chinese Title: Intelligent]) OR "Acinetobacter baumannii OR A. baumannii"[Abstract: Intelligent] | 18327 |
| #2 | "xuebijing OR XBJ"[Full Text: Intelligent] OR "xuebijing OR XBJ"[Chinese Title: Intelligent] OR "xuebijing OR XBJ"[Abstract: Intelligent] | 3650 |
| #3 | #1 AND #2 | 12 |
| **Last Run Date: 09/02/2025** | | |

**Supplementary Material 2:** Subgroup analysis of overall effective rate.


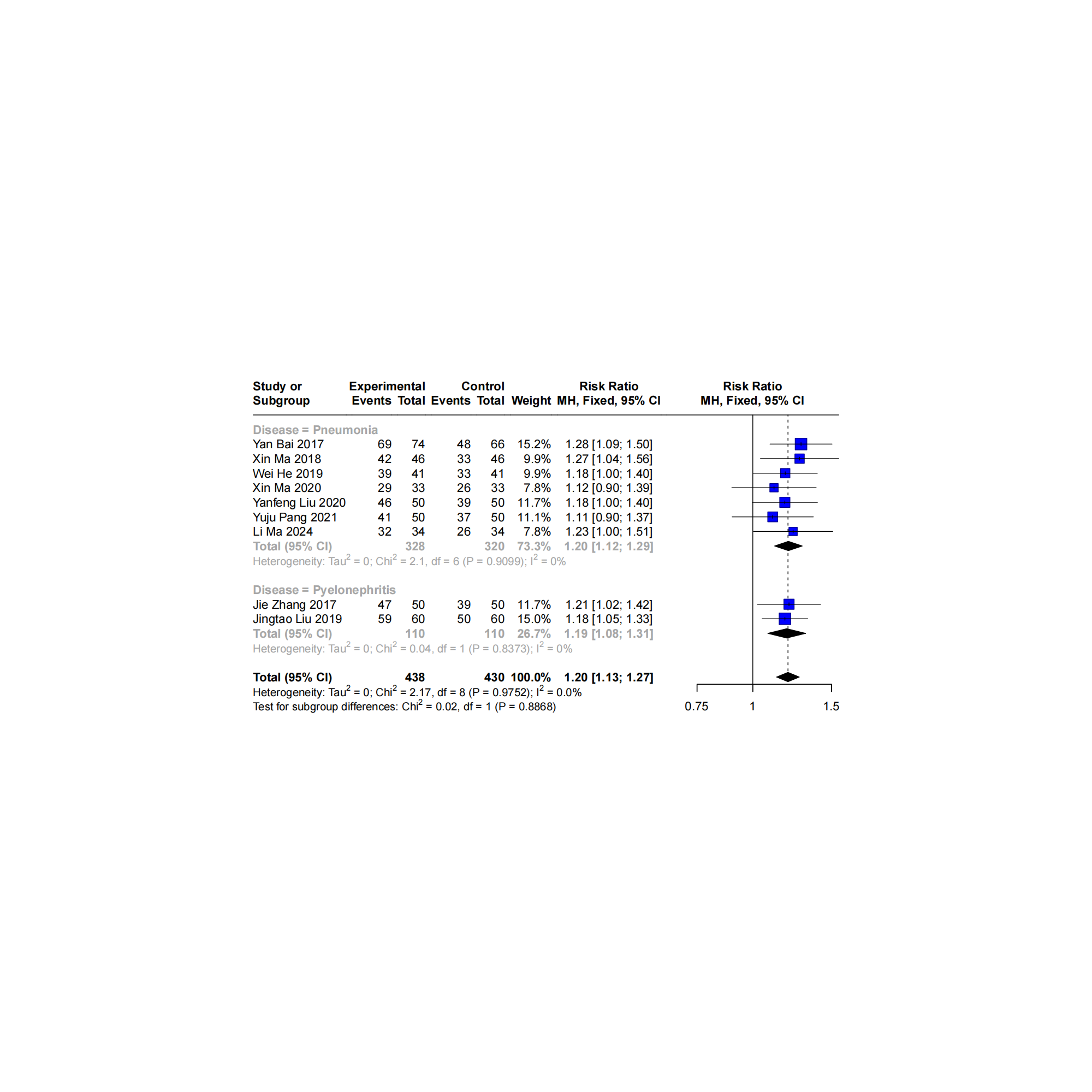


**Supplementary Figure 1.** Subgroup analysis of overall effective rate based on disease types (Pneumonia vs. Pyelonephritis).


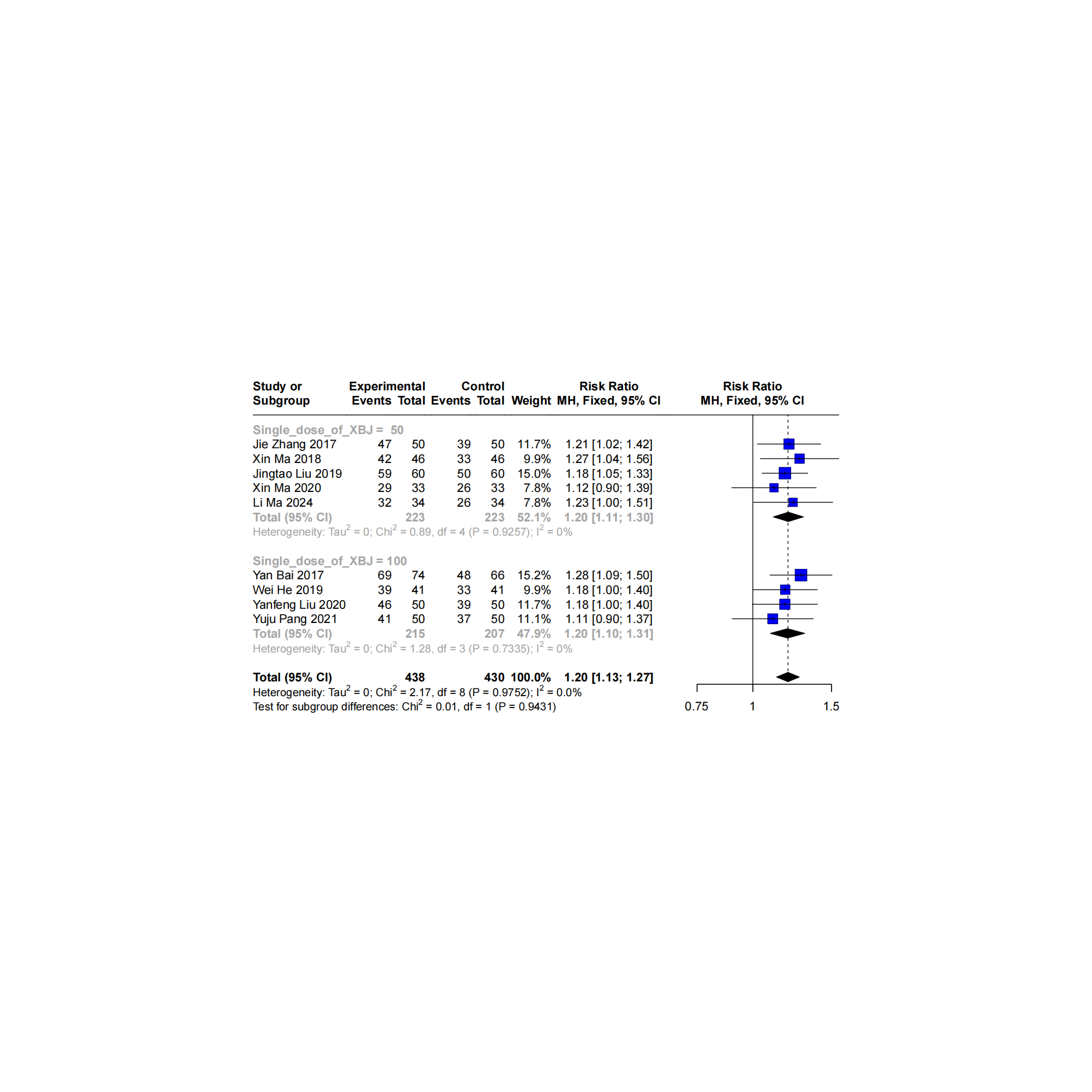


**Supplementary Figure 2.** Subgroup analysis of overall effective rate based on single dose of XBJ (≤ 50 mL/dose vs. ≤ 100 mL/dose).


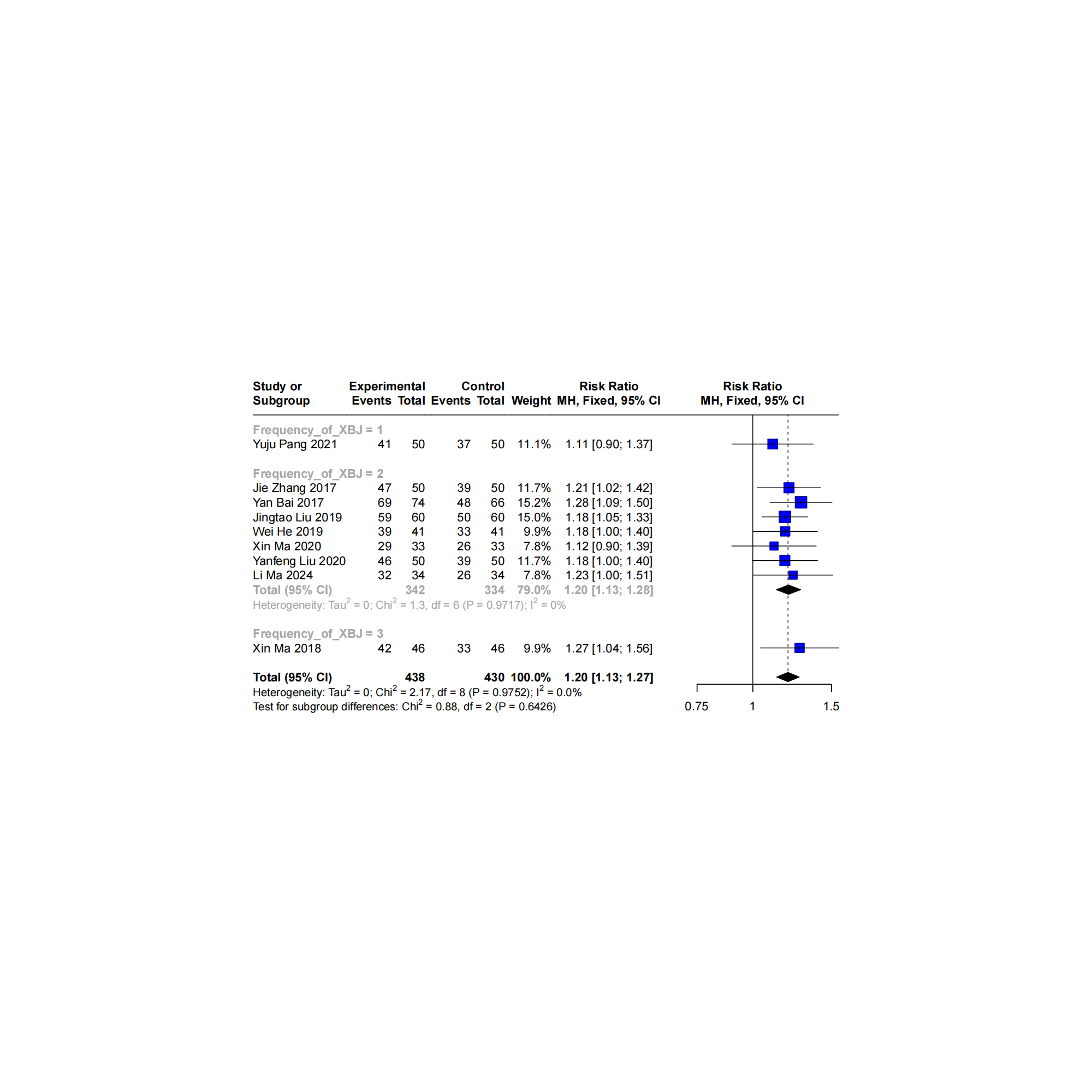


**Supplementary Figure 3.** Subgroup analysis of overall effective rate based on frequency of XBJ (Once daily vs. Twice daily vs. Thrice daily).


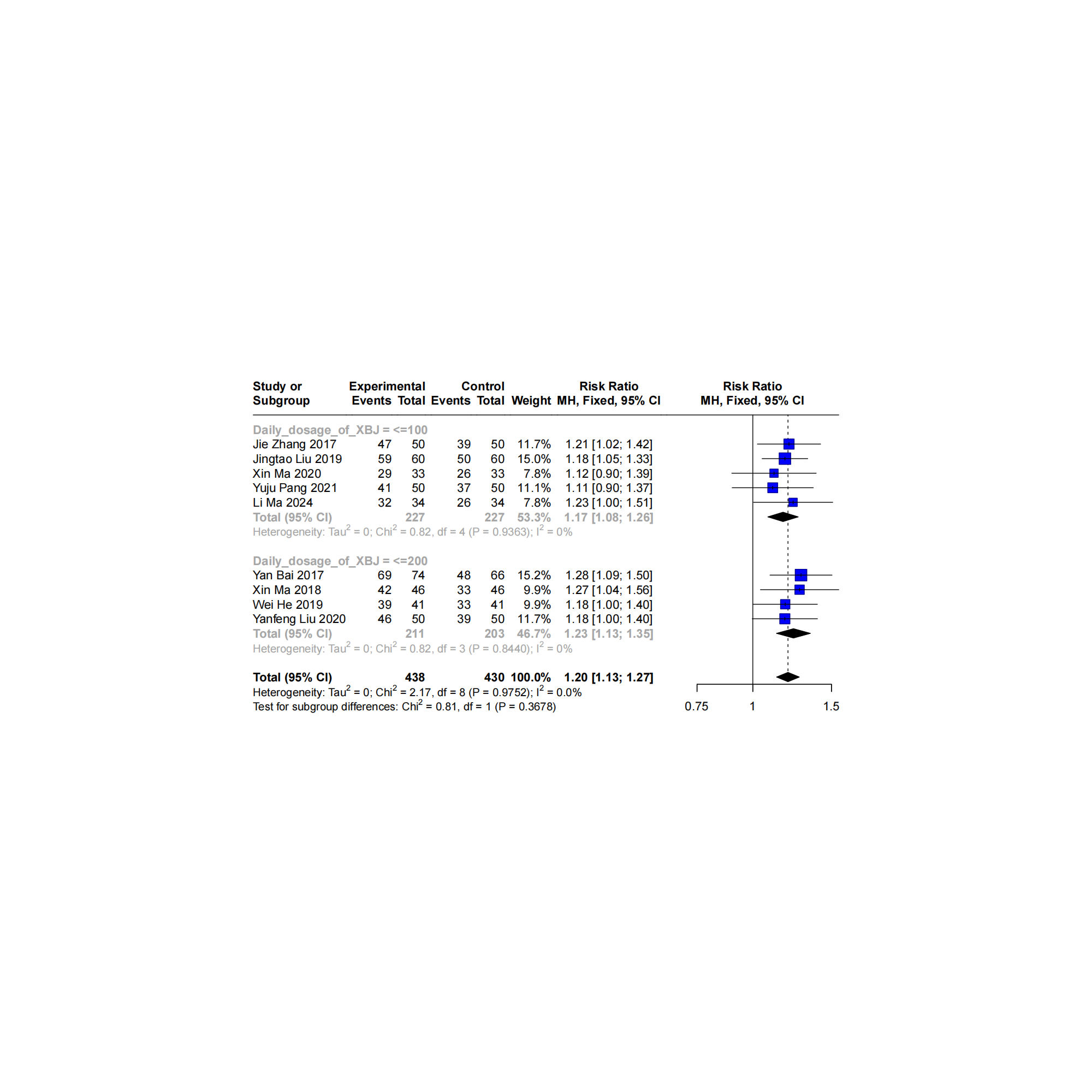


**Supplementary Figure 4.** Subgroup analysis of overall effective rate based on daily dosage of XBJ (≤ 100 mL/day vs. ≤ 200 mL/day).


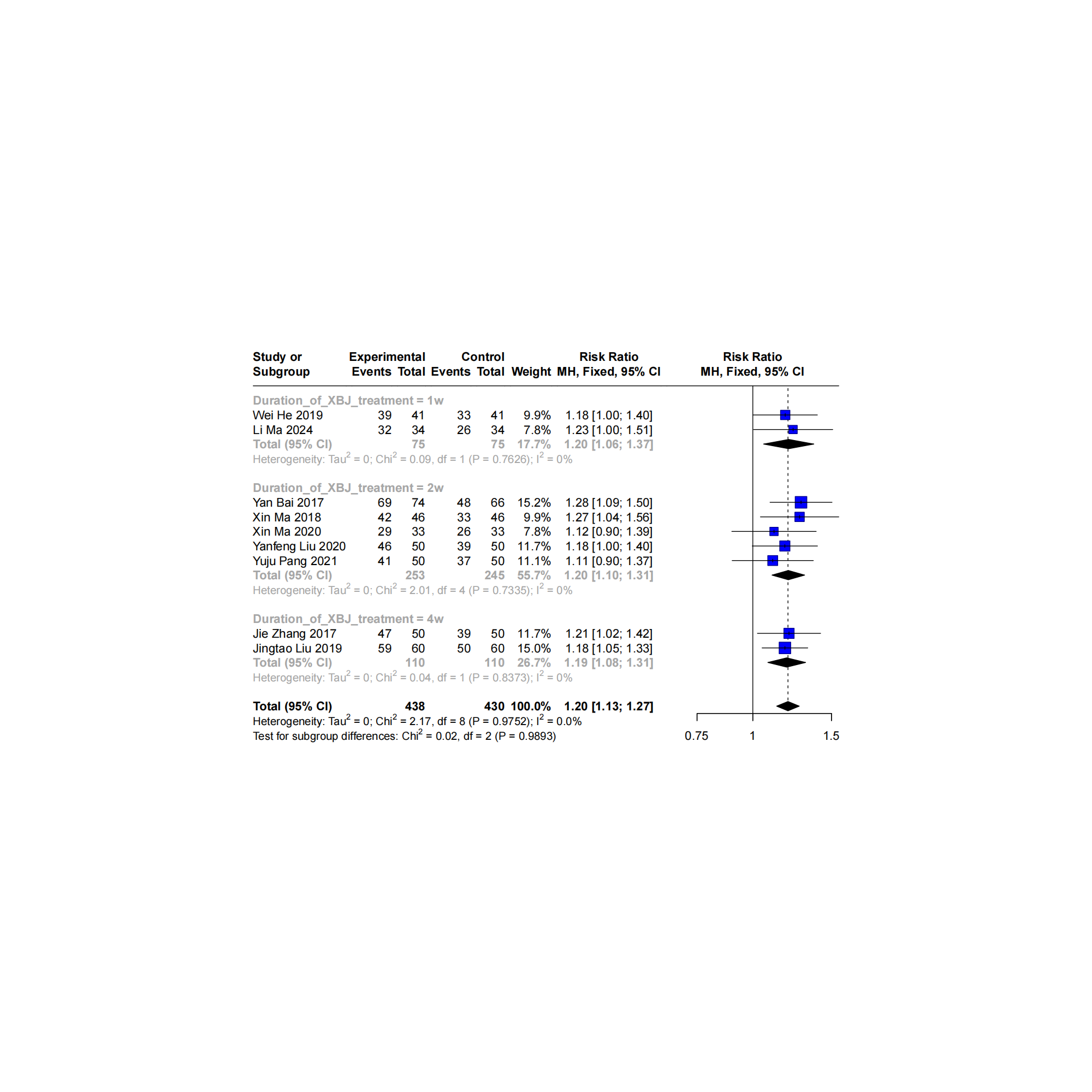


**Supplementary Figure 5.** Subgroup analysis of overall effective rate based on treatment duration of XBJ (≤ 1 week vs. ≤ 2 weeks vs. ≤ 4 weeks).


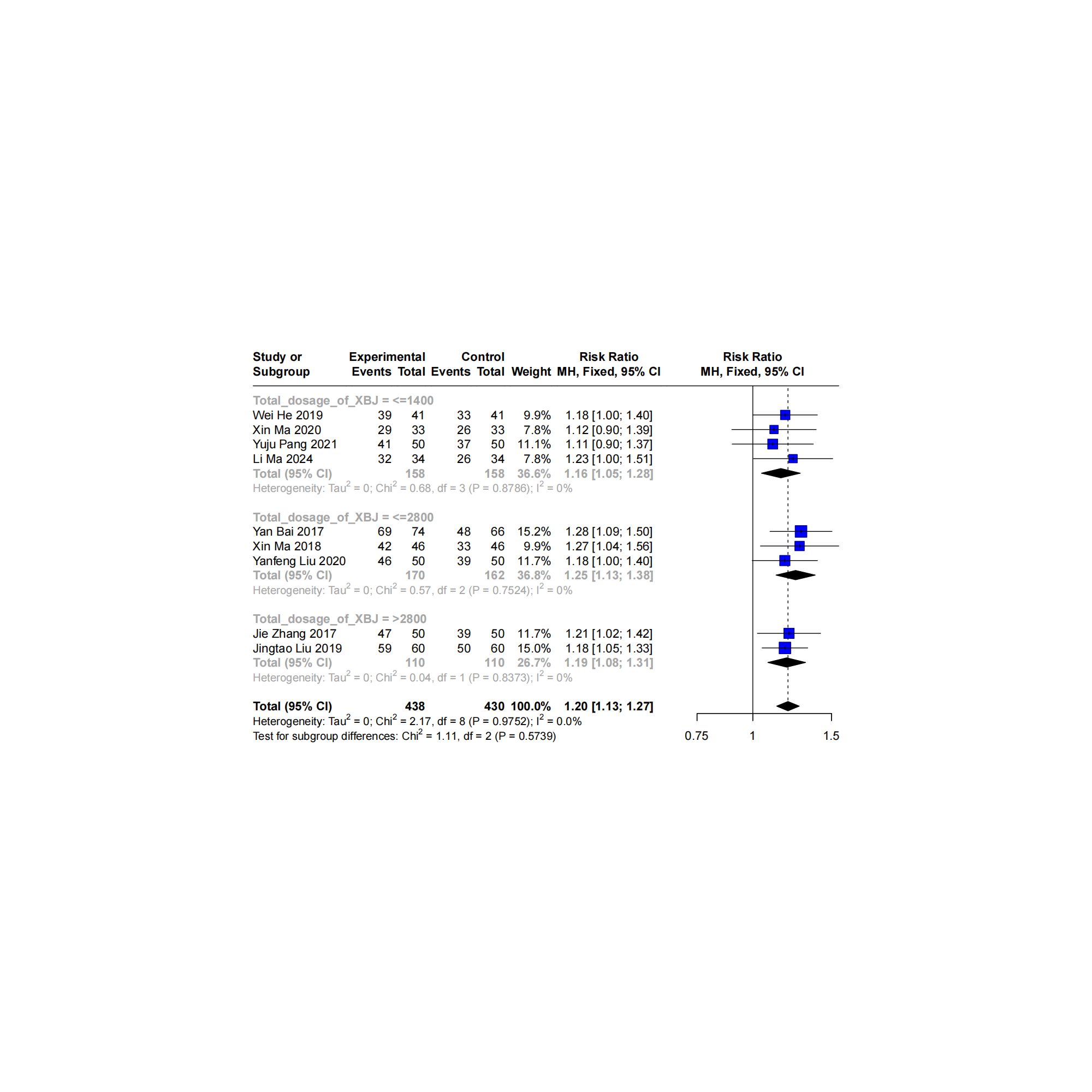


**Supplementary Figure 6.** Subgroup analysis of overall effective rate based on total dosage of XBJ (≤ 1400 mL vs. ≤ 2800 mL vs. > 2800 mL).

**Supplementary Material 3:** Sensitivity and subgroup analyses of CRP.


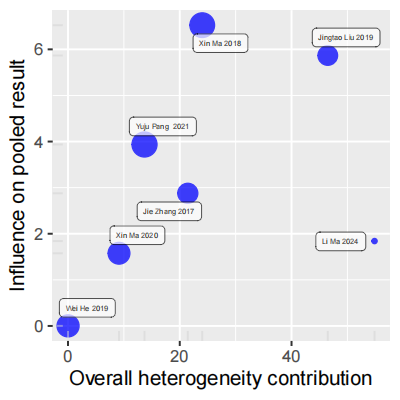


**Supplementary Figure 1.** Sensitivity analysis using baujat plot of CRP.


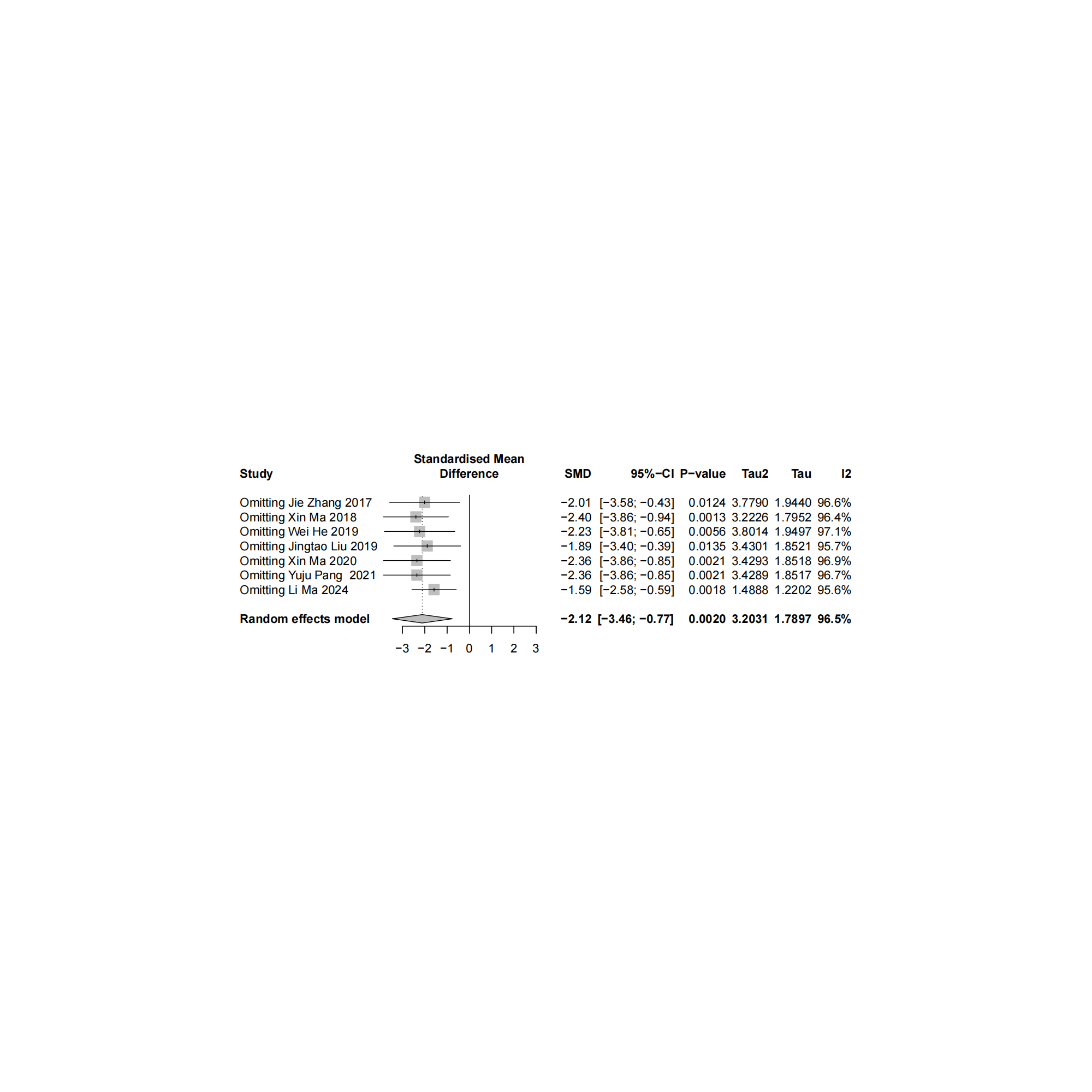


**Supplementary Figure 2.** Sensitivity analysis using leave-one-out method of CRP.


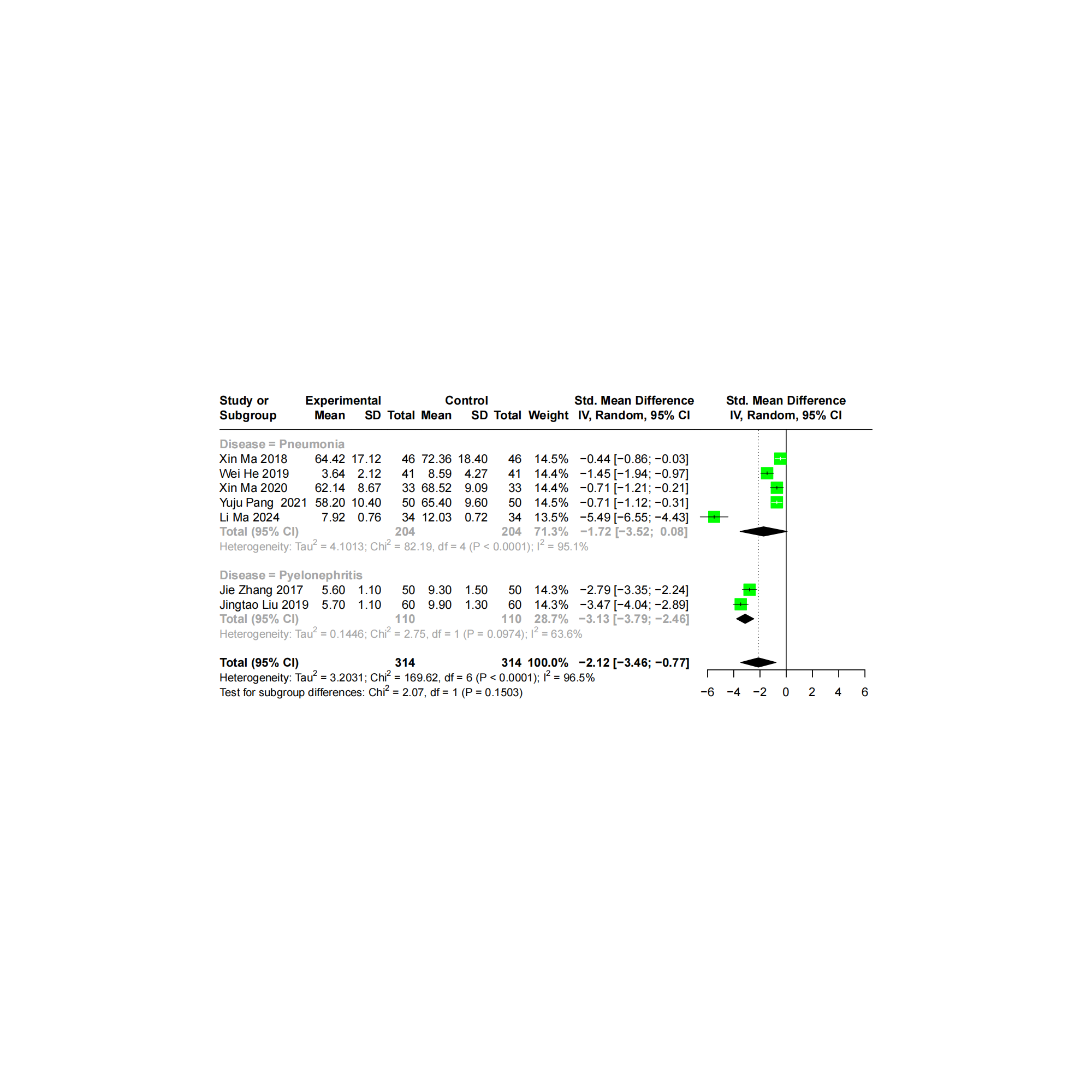


**Supplementary Figure 3.** Subgroup analysis of CRP based on disease types (Pneumonia vs. Pyelonephritis).


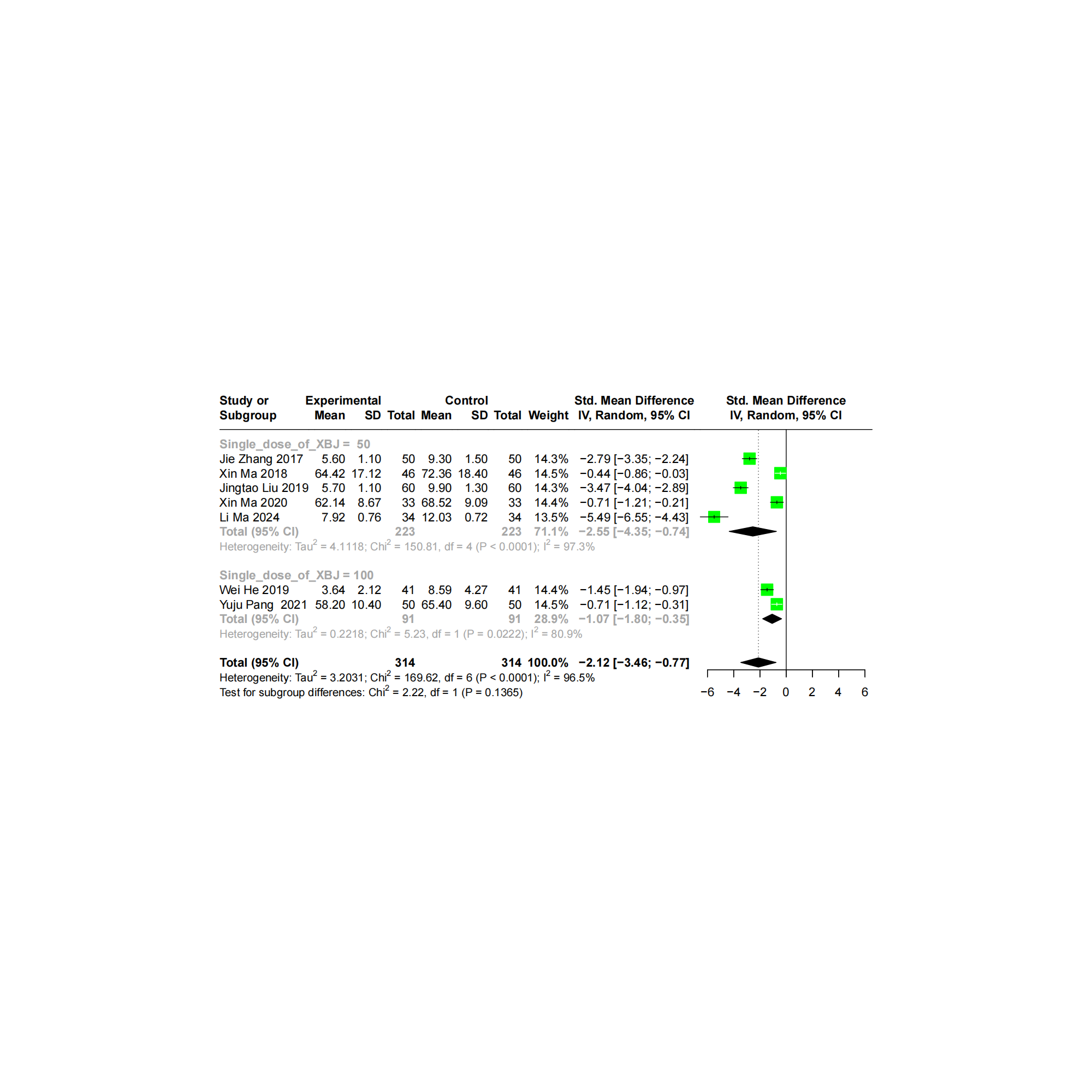


**Supplementary Figure 4.** Subgroup analysis of CRP based on single dose of XBJ (≤ 50 mL/dose vs. ≤ 100 mL/dose).


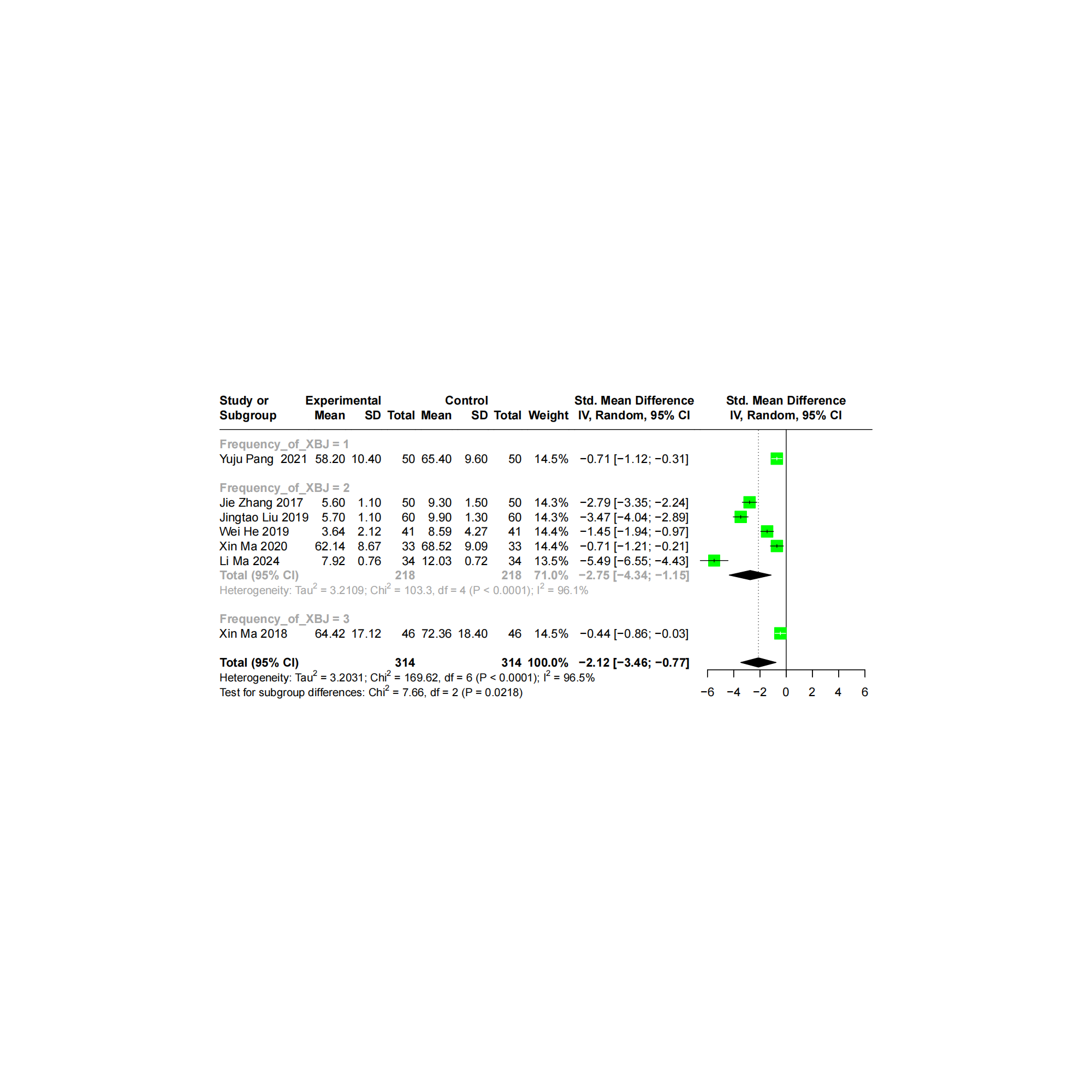


**Supplementary Figure 5.** Subgroup analysis of CRP based on frequency of XBJ (Once daily vs. Twice daily vs. Thrice daily).


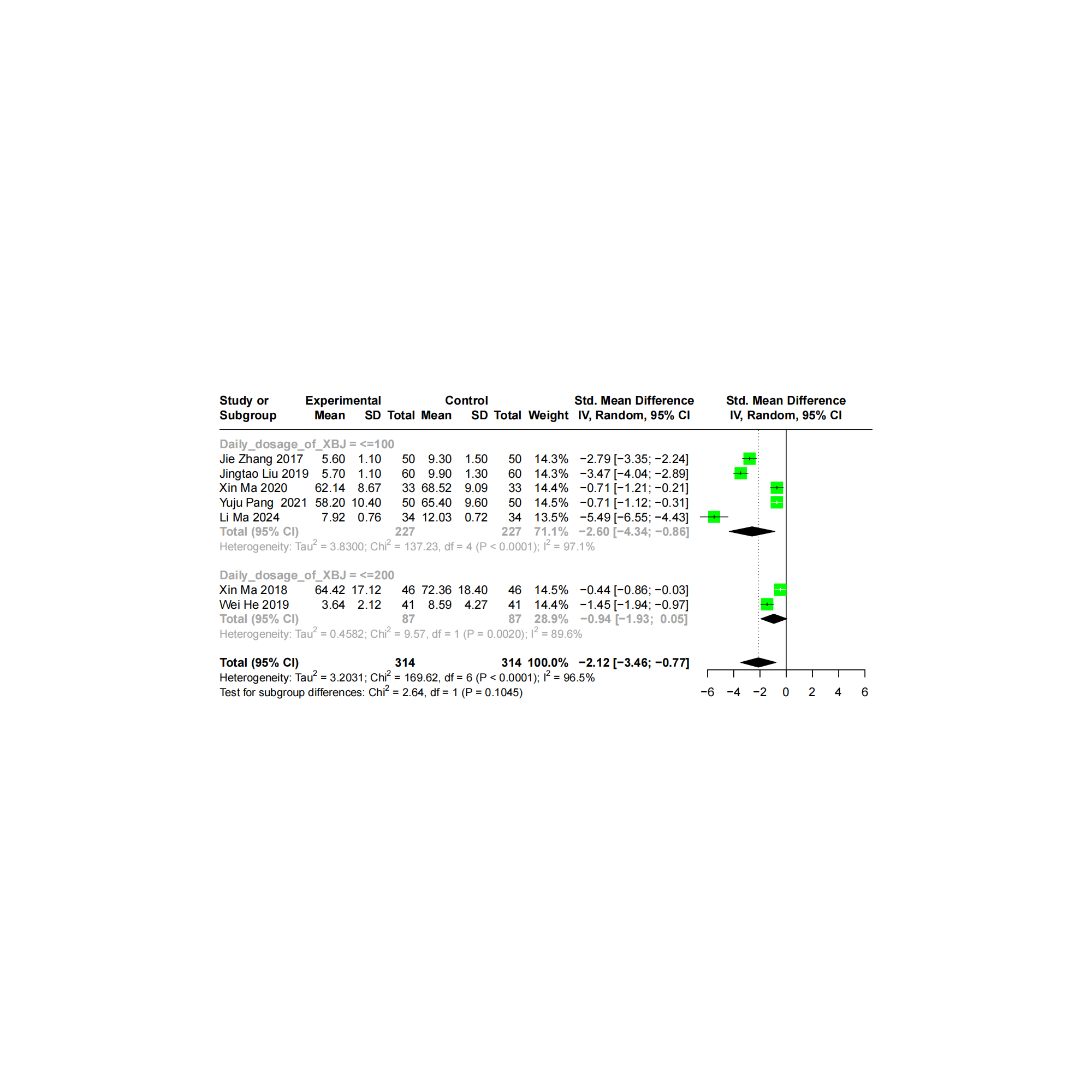


**Supplementary Figure 6.** Subgroup analysis of CRP based on daily dosage of XBJ (≤ 100 mL/day vs. ≤ 200 mL/day).


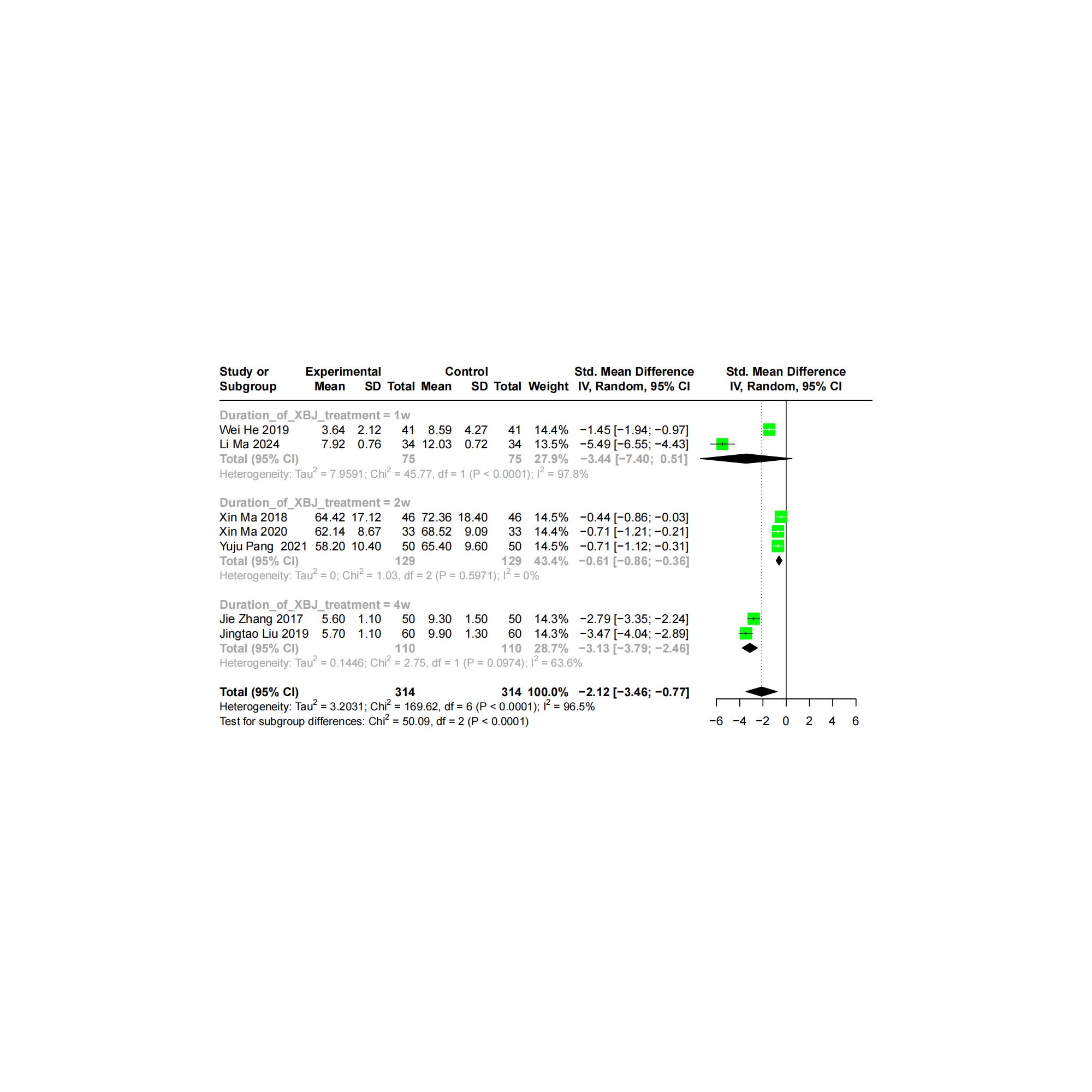


**Supplementary Figure 7.** Subgroup analysis of CRP based on treatment duration of XBJ (≤ 1 week vs. ≤ 2 weeks vs. ≤ 4 weeks).


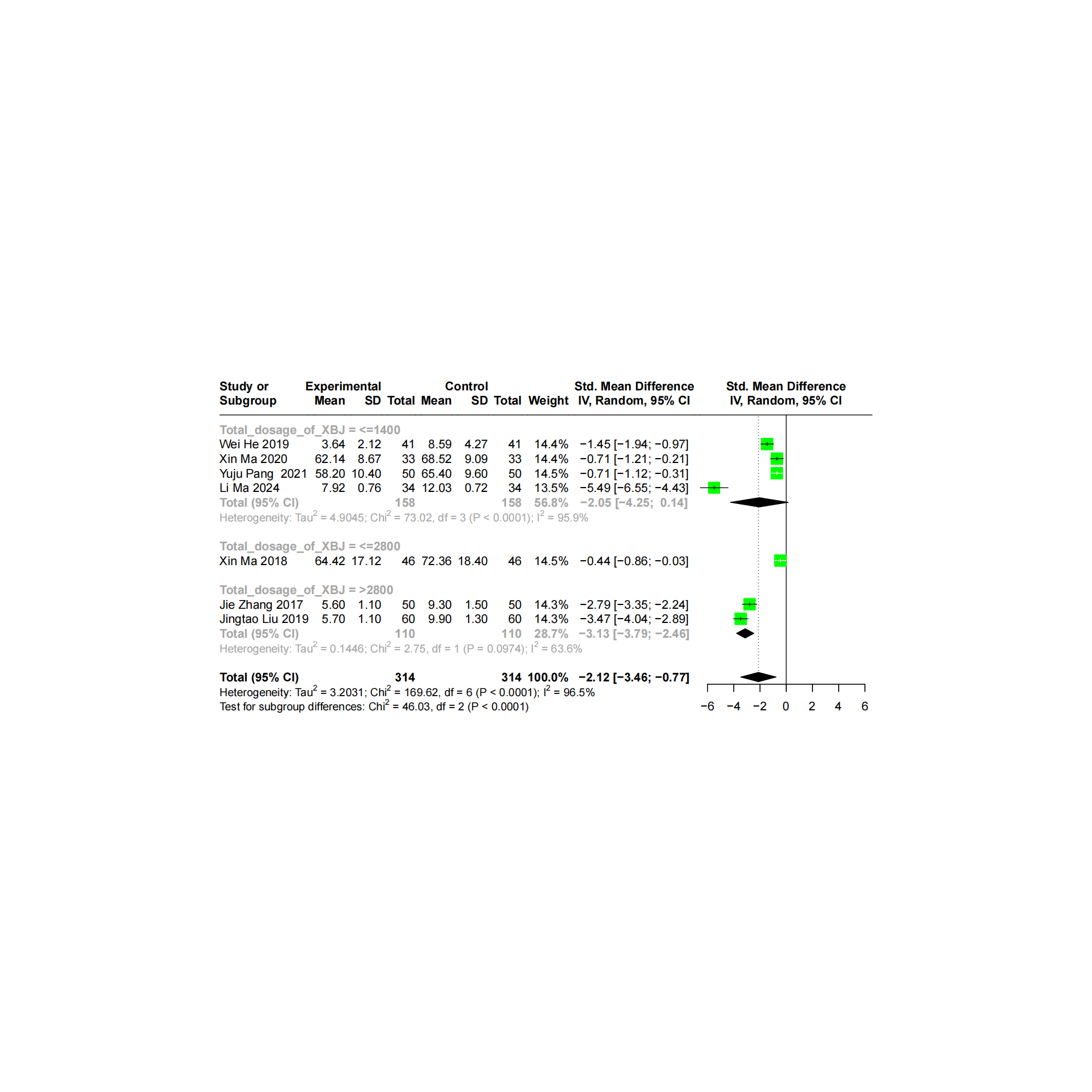


**Supplementary Figure 8.** Subgroup analysis of CRP based on total dosage of XBJ (≤ 1400 mL vs. ≤ 2800 mL vs. > 2800 mL).

**Supplementary Material 4:** Sensitivity analyses of PCT.


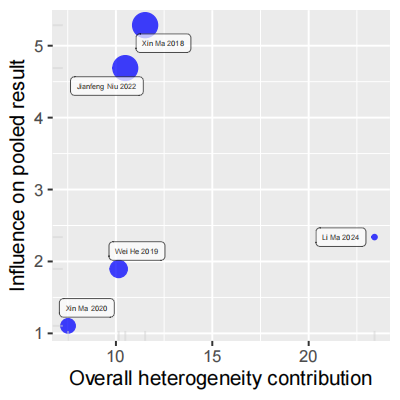


**Supplementary Figure 1.** Sensitivity analysis using baujat plot of PCT.


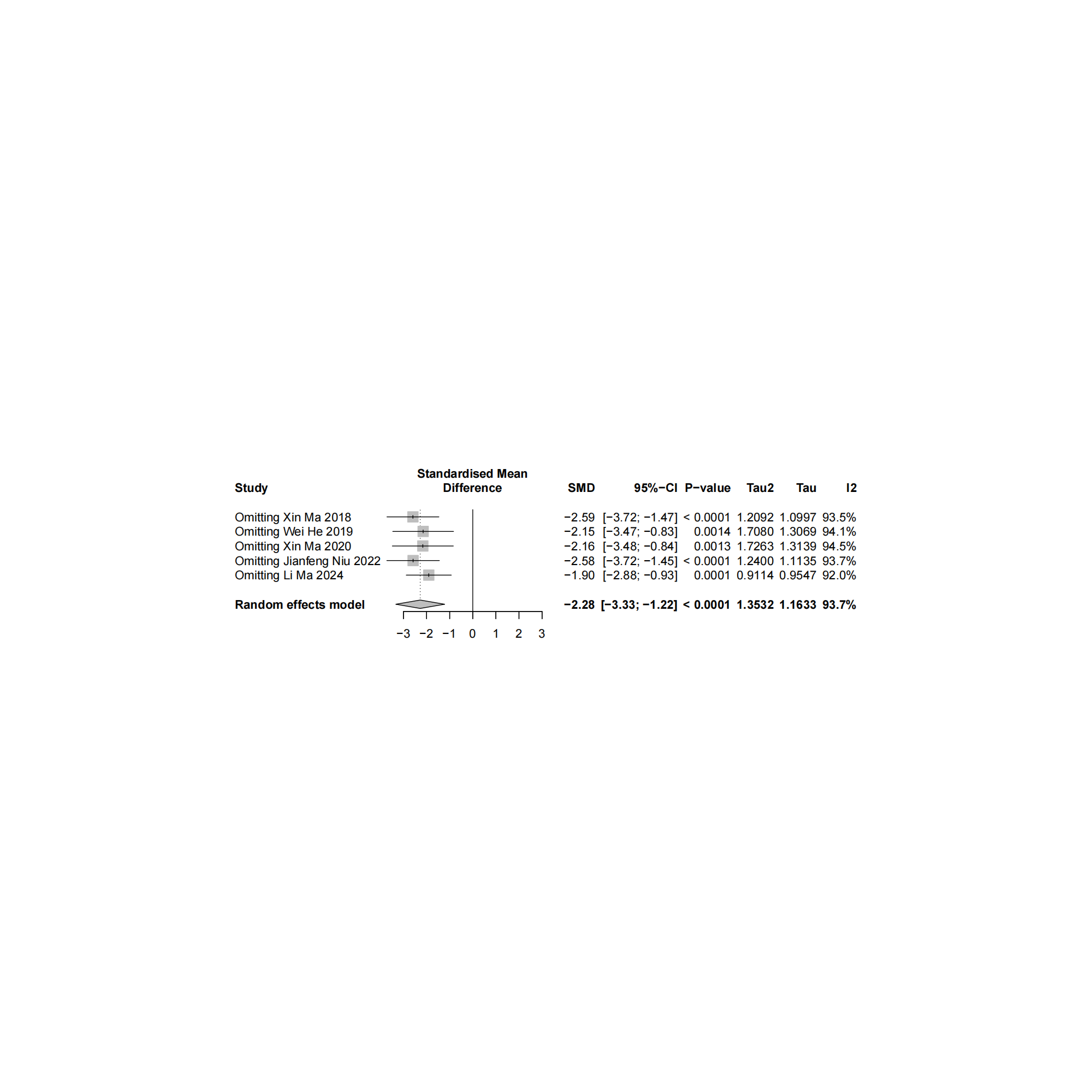


**Supplementary Figure 2.** Sensitivity analysis using leave-one-out method of PCT.

**Supplementary Material 5:** Sensitivity analyses of WBC.


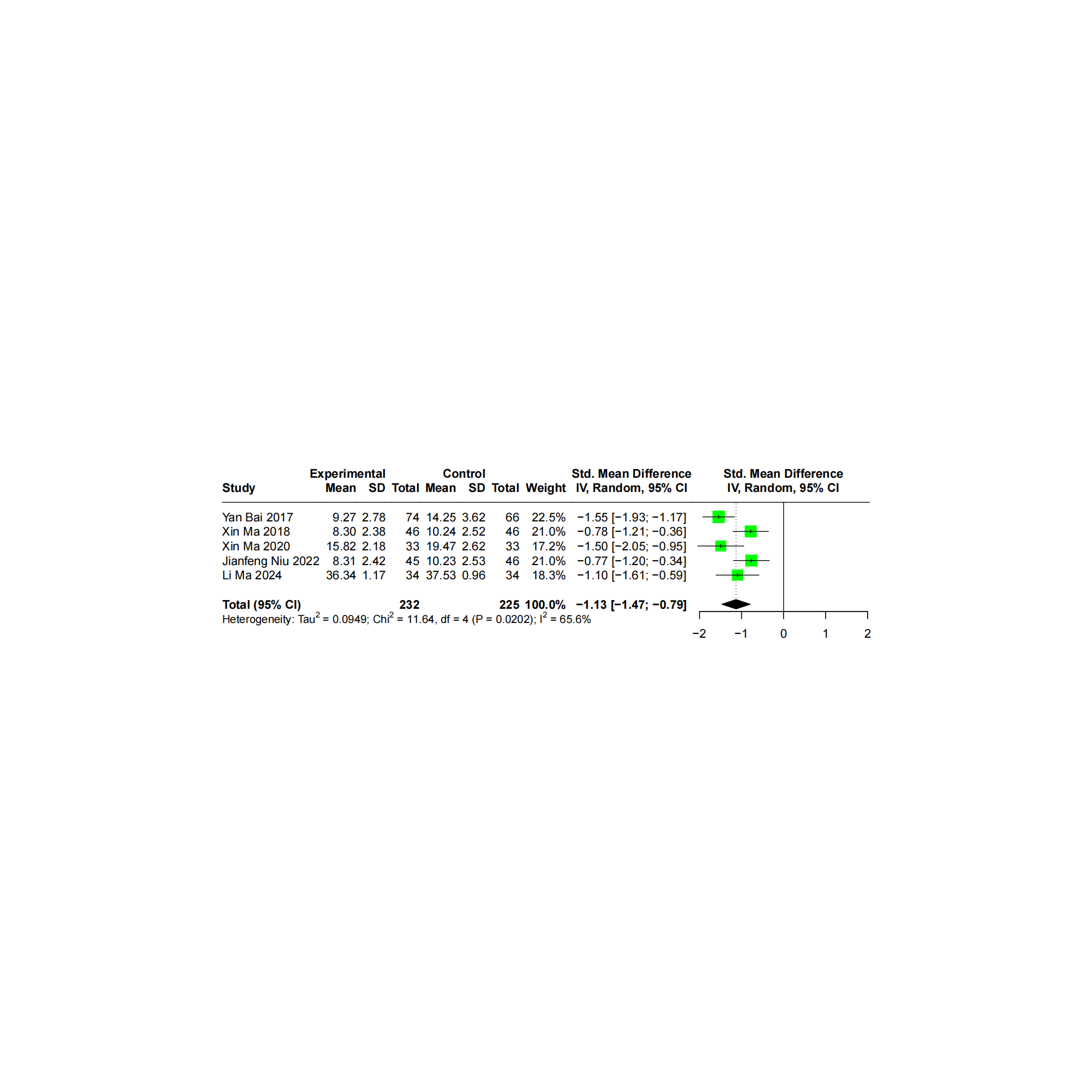


**Supplementary Figure 1.** The original forest plot, generated using a random-effects model, reveals significant heterogeneity across the included studies.


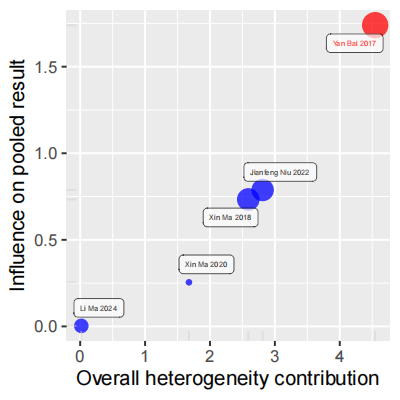


**Supplementary Figure 2.** Sensitivity analysis using baujat plot of WBC. (*red spot represents outlier study*)


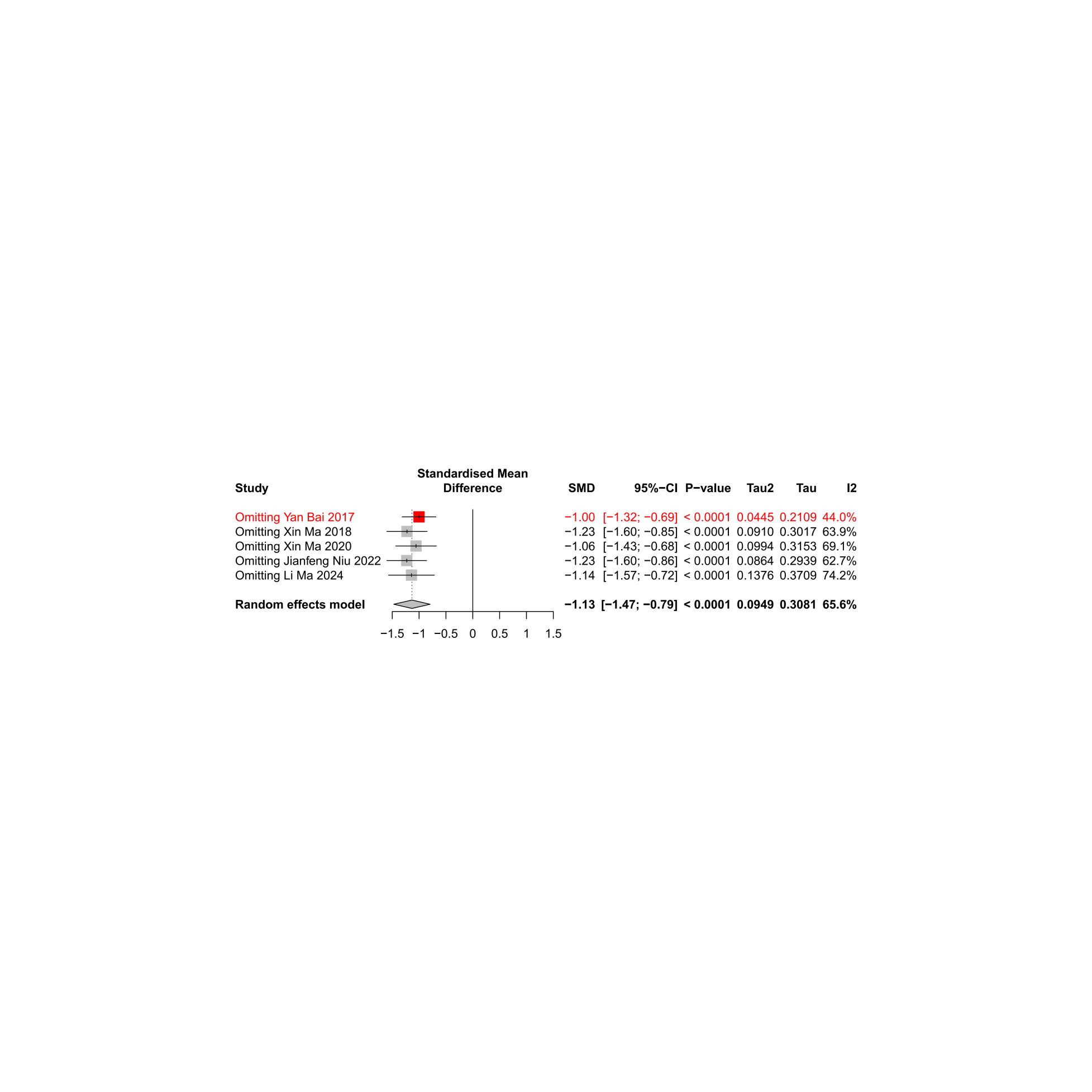


**Supplementary Figure 3.** Sensitivity analysis using leave-one-out method of WBC. (*red line represents outlier study*)

**Supplementary Material 6:** Active ingredients of XBJ (145 ingredients).

| **No.** | **Ingredients name** | **CID number** | **No.** | **Ingredients name** | **CID number** |
| --- | --- | --- | --- | --- | --- |
| 1 | Protocatechuic acid | 72 | 2 | 1,5-Dicaffeoylquinic acid | 5281769 |
| 3 | p-Hy(roxybenzoic acid | 135 | 4 | Isochlorogenic acid B | 5281780 |
| 5 | adenine | 190 | 6 | Rosmarinic acid | 5281792 |
| 7 | Benzoic acid | 243 | 8 | Salvianolic acid A | 5281793 |
| 9 | Gallic acid | 370 | 10 | Kaempferol 3-glucoside | 5282102 |
| 11 | Succinic acid | 1110 | 12 | Z-6,7-Epoxyligustilide | 5317139 |
| 13 | Thymine | 1135 | 14 | Galuteolin | 5317471 |
| 15 | Uracil | 1174 | 16 | Isorhamnetin 3-glucoside | 5318645 |
| 17 | Vanillin | 1183 | 18 | Kaempferol-3-O-rutinoside | 5318767 |
| 19 | Azelaic acid | 2266 | 20 | Ligustilide | 5319022 |
| 21 | 4-Hydroxytoluene;(4-Methylphenol) | 2879 | 22 | N-Coumaroyl serotonin | 5458879 |
| 23 | D (+)-Glucose | 5793 | 24 | 3,7- or 3,8-Dimethyl ellagic acid | 5488919 |
| 25 | Sucrose | 5988 | 26 | Salviaflaside | 6438919 |
| 27 | Uridine | 6029 | 28 | Lithospermic acid | 6441498 |
| 29 | L-Leucine | 6106 | 30 | Hydroxysafflor yellow A | 6443665 |
| 31 | Phenylalanine | 6140 | 32 | geranyl beta-primeveroside | 6443689 |
| 33 | Cytidine | 6175 | 34 | Safflor yellow A | 6448299 |
| 35 | (-)-Quinic acid | 6508 | 36 | Salvianolic acid B | 6451084 |
| 37 | Methyl gallate | 7428 | 38 | Quercetin-O-2glu/gal | 6453392 |
| 39 | p-Anisic acid (4-Methoxybenzoic Acid) | 7478 | 40 | Isochlorogenic acid C | 6474309 |
| 41 | Vanillic acid | 8468 | 42 | Cryptochlorogenic acid | 9798666 |
| 43 | Protocatechuic aldehyde | 8768 | 44 | Tanshinone IIB | 9926694 |
| 45 | Paeonol | 11092 | 46 | Senkyunolide G | 10013283 |
| 47 | Ethyl gallate | 13250 | 48 | Quercetin 3,7-diglucoside | 10121947 |
| 49 | Tetramethylpyrazine | 14296 | 50 | 1'-O-Benzoylsucrose | 10225582 |
| 51 | Prenyl benzoate | 21265 | 52 | Quercetin 3-rha-(1-6)-glucoside | 10283929 |
| 53 | Adenosine | 60961 | 54 | Mudanpioside | 10576690 |
| 55 | 3-Hydroxy-3-n-butylphthalide | 61361 | 56 | Miltiodiol | 11011966 |
| 57 | Pentagalloylglucose | 65238 | 58 | Senkyunolide F | 11241196 |
| 59 | Tetragalloyl glucose | 73178 | 60 | Senkyunolide M | 11265913 |
| 61 | Matrine | 91466 | 62 | Dihydrotanshinone I | 11425923 |
| 63 | Ribonolactone | 111064 | 64 | Salvianolic acid G | 11530200 |
| 65 | Tanshinone I | 114917 | 66 | danshensu | 11600642 |
| 67 | Tanshinaldehyde | 124268 | 68 | Picroside II | 11944602 |
| 69 | 1,2,5,6-tetrahydrotanshinone I | 124416 | 70 | Salvianolic acid C | 13991590 |
| 71 | Tanshinone VI | 149138 | 72 | 6'-O-Galloylsucrose | 14055557 |
| 73 | cryptotanshinone | 160254 | 74 | 1-O-galloylsucrose | 14055559 |
| 75 | Tanshinone II A | 164676 | 76 | 6-Hydroxykaempferol 3,6-diglucoside | 14375137 |
| 77 | Tanshinone V | 184103 | 78 | Desbenzoylpaeoniflorin | 15450040 |
| 79 | Scutellarin | 185617 | 80 | Deoxyneocryptotanshinone | 15690458 |
| 81 | Carthamidin | 188308 | 82 | Citric acid | 19782904 |
| 83 | Hydroxymethylfurfural | 237332 | 84 | Mudanpioside J | 21593828 |
| 85 | Naringenin | 439246 | 86 | 30-Norhederagenin | 21596158 |
| 87 | Salvianic acid A sodium | 439435 | 88 | Mudanpioside C | 21631098 |
| 89 | 4-Glucosyloxybenzoic acid | 440186 | 90 | Mudanpioside D | 21631103 |
| 91 | Eriodictyol | 440735 | 92 | Oxypaeoniflorin | 21631105 |
| 93 | Paeoniflorin | 442534 | 94 | Benzoylpaeoniflorin | 21631106 |
| 95 | Ferulic acid | 445858 | 96 | Benzoyloxypaeoniflorin | 21631107 |
| 97 | Trigalloyl glucose | 452707 | 98 | Mudanpioside F | 21631108 |
| 99 | Galloylpaeoniflorin | 494717 | 100 | Butanedioic acid | 21952380 |
| 101 | p-Coumaric acid | 637542 | 102 | Sodium Danshensu | 23711819 |
| 103 | Butylidenephthalide | 642376 | 104 | Salvianolic acid J | 24177556 |
| 105 | Hexadecasphinganine | 656816 | 106 | Albiflorin | 24868421 |
| 107 | caffeic acid | 689043 | 108 | Safflomin C | 42607658 |
| 109 | caffeic acid | 1549111 | 110 | 6-Hydroxykaempferol 3-glucoside | 44259730 |
| 111 | Ethyl ferulate | 736681 | 112 | 6-Hydroxykaempferol 7-glucoside | 44259740 |
| 113 | Chlorogenic acid | 1794427 | 114 | Isosalvianolic acid C | 44566967 |
| 115 | Galloyloxypaeoniflorin | 3036133 | 116 | Saffloquinoside A | 45276863 |
| 117 | Neocnidilide | 3083857 | 118 | Neocarthamin | 46173973 |
| 119 | Senkyunolide A | 3085257 | 120 | Levistolide A | 70698035 |
| 121 | Quercetin | 5280343 | 122 | Monogalloyl glucose | 71308210 |
| 123 | apigenin | 5280443 | 124 | Salvianolic acid D | 75412558 |
| 125 | Luteolin | 5280445 | 126 | Salvianolic acid E | 86278266 |
| 127 | Coniferyl aldehyde/(ferulaldehyde) | 5280536 | 128 | Mudanpioside E | 86278277 |
| 129 | Neochlorogenic acid | 5280633 | 130 | bis-peg8-acid | 118796285 |
| 131 | Luteoloside | 5280637 | 132 | 6-Hydroxykaempferol 6,7-diglucoside | 131752759 |
| 133 | Quercetin 3-glucoside | 5280804 | 134 | Saffloquinoside E | 134714969 |
| 135 | Rutin | 5280805 | 136 | Guanosine | 135398635 |
| 137 | kaempferol | 5280863 | 138 | Ethyl4-hydroxy-3-methoxycinnamate | 135452442 |
| 139 | Senkyunolide B | 5281559 | 140 | Carthamin | 135565560 |
| 141 | 6-Hydroxykaempferol | 5281638 | 142 | Salvianic acid C | 162823517 |
| 143 | Hyperoside | 5281643 | 144 | 4-Hydroxy-3-n-butylphthalide | 163688252 |
| 145 | 6-Hydroxyapigenin | 5281697 |  |  |  |

**Supplementary Material 7:** Targets of XBJ ingredients predicted from SwissTargetPrediction website (758 targets with probability > 0.1).

| **No.** | **Targets name** | **No.** | **Targets name** | **No.** | **Targets name** |
| --- | --- | --- | --- | --- | --- |
| 1 | ABCB1 | 2 | ABCC1 | 3 | ABCC9 |
| 4 | ABCG2 | 5 | ABL1 | 6 | ACE |
| 7 | ACHE | 8 | ACLY | 9 | ACP1 |
| 10 | ADA | 11 | ADAM10 | 12 | ADAM17 |
| 13 | ADAMTS4 | 14 | ADAMTS5 | 15 | ADK |
| 16 | ADORA1 | 17 | ADORA2A | 18 | ADORA2B |
| 19 | ADORA3 | 20 | ADRA1A | 21 | ADRA1D |
| 22 | ADRA2A | 23 | ADRA2B | 24 | ADRA2C |
| 25 | ADRB2 | 26 | AGTR1 | 27 | AHCY |
| 28 | AHR | 29 | AKR1A1 | 30 | AKR1B1 |
| 31 | AKR1B10 | 32 | AKR1C1 | 33 | AKR1C2 |
| 34 | AKR1C3 | 35 | AKR1C4 | 36 | AKT1 |
| 37 | AKT2 | 38 | ALB | 39 | ALDH2 |
| 40 | ALK | 41 | ALOX12 | 42 | ALOX15 |
| 43 | ALOX5 | 44 | ALOX5AP | 45 | ALPG |
| 46 | ALPL | 47 | AMD1 | 48 | AMPD1 |
| 49 | AMPD2 | 50 | AMPD3 | 51 | AMY1A |
| 52 | AMY2A | 53 | ANPEP | 54 | APEX1 |
| 55 | APP | 56 | AR | 57 | ARG1 |
| 58 | ASNS | 59 | ATIC | 60 | ATP1A1 |
| 61 | AURKA | 62 | AURKB | 63 | AVPR2 |
| 64 | AXL | 65 | BACE1 | 66 | BACE2 |
| 67 | BAZ2A | 68 | BAZ2B | 69 | BCHE |
| 70 | BCL2 | 71 | BCL2A1 | 72 | BCL2L1 |
| 73 | BDKRB1 | 74 | BLK | 75 | BMP1 |
| 76 | BMX | 77 | BRAF | 78 | BRD2 |
| 79 | BRD3 | 80 | BRD4 | 81 | BRS3 |
| 82 | BTK | 83 | C1R | 84 | CA1 |
| 85 | CA12 | 86 | CA13 | 87 | CA14 |
| 88 | CA2 | 89 | CA3 | 90 | CA4 |
| 91 | CA5A | 92 | CA5B | 93 | CA6 |
| 94 | CA7 | 95 | CA9 | 96 | CACNA1C |
| 97 | CACNA2D1 | 98 | CALM1 | 99 | CAMK1 |
| 100 | CAMK2B | 101 | CAMK2D | 102 | CAMK4 |
| 103 | CAPN1 | 104 | CARM1 | 105 | CASP1 |
| 106 | CASP2 | 107 | CASP3 | 108 | CASP6 |
| 109 | CASP7 | 110 | CASP8 | 111 | CASR |
| 112 | CBR1 | 113 | CCNA2 | 114 | CCNA2 |
| 115 | CCNB1 | 116 | CCNB3 | 117 | CCNE1 |
| 118 | CCNE1 | 119 | CCNE1 CCR1 | 120 | CCNE2 |
| 121 | CCR9 | 122 | CD38 | 123 | CD81 |
| 124 | CDA | 125 | CDC25A | 126 | CDC25B |
| 127 | CDC25C | 128 | CDC42 | 129 | CDK1 |
| 130 | CDK2 | 131 | CCNA1 | 132 | CDK5R1 |
| 133 | CDK6 | 134 | CDK9 | 135 | CDK9 |
| 136 | CES1 | 137 | CES2 | 138 | CFD |
| 139 | CFTR | 140 | CHEK1 | 141 | CHEK2 |
| 142 | CHRM1 | 143 | CHRM2 | 144 | CHRM3 |
| 145 | CHRM4 | 146 | CHRM5 | 147 | CHRNA3 |
| 148 | CHRNB2 | 149 | CHRNB4 | 150 | CISD1 |
| 151 | CLK1 | 152 | CMA1 | 153 | CNR1 |
| 154 | CNR2 | 155 | COMT | 156 | COQ8B |
| 157 | CPA3 | 158 | CPB1 | 159 | CPB2 |
| 160 | CREBBP | 161 | CRHR1 | 162 | CSF1R |
| 163 | CSK | 164 | CSNK1A1 | 165 | CSNK1D |
| 166 | CSNK1G1 | 167 | CSNK1G2 | 168 | CSNK2A1 |
| 169 | CTRB1 | 170 | CTSB | 171 | CTSC |
| 172 | CTSG | 173 | CTSH | 174 | CTSK |
| 175 | CTSL | 176 | CTSS | 177 | CTSV |
| 178 | CXCL8 | 179 | CXCR1 | 180 | CXCR2 |
| 181 | CYP11B1 | 182 | CYP11B2 | 183 | CYP17A1 |
| 184 | CYP19A1 | 185 | CYP1A1 | 186 | CYP1A2 |
| 187 | CYP1B1 | 188 | CYP24A1 | 189 | CYP26A1 |
| 190 | CYP27A1 | 191 | CYP2C19 | 192 | CYP2C9 |
| 193 | CYP2D6 | 194 | CYP3A4 | 195 | CYP51A1 |
| 196 | DAO | 197 | DAPK1 | 198 | DAPK3 |
| 199 | DHFR | 200 | DHODH | 201 | DNM1 |
| 202 | DNMT1 | 203 | DNMT3B | 204 | DOT1L |
| 205 | DPP4 | 206 | DRD1 | 207 | DRD2 |
| 208 | DRD3 | 209 | DRD4 | 210 | DSTYK |
| 211 | DUSP1 | 212 | DUSP3 | 213 | DYRK1A |
| 214 | DYRK1B | 215 | DYRK2 | 216 | ECE1 |
| 217 | EDNRA | 218 | EDNRB | 219 | EED SUZ12 |
| 220 | EED SUZ12 | 221 | EGLN1 | 222 | EGLN3 |
| 223 | EHMT1 | 224 | EHMT2 | 225 | EIF4E |
| 226 | EIF4H | 227 | ELANE | 228 | ELAVL1 |
| 229 | ELOVL6 | 230 | ENPEP | 231 | EP300 |
| 232 | EPHA2 | 233 | EPHB4 | 234 | EPHX1 |
| 235 | EPHX2 | 236 | ERBB2 | 237 | ERN1 |
| 238 | ESR1 | 239 | ESR2 | 240 | ESRRA |
| 241 | ESRRB | 242 | EZH1 | 243 | EZH2 |
| 244 | EZH2 | 245 | F10 | 246 | F13A1 |
| 247 | F2 | 248 | F2R | 249 | F3 |
| 250 | F3 F7 | 251 | F7 | 252 | FAAH |
| 253 | FABP1 | 254 | FABP2 | 255 | FABP3 |
| 256 | FABP4 | 257 | FABP5 | 258 | FAP |
| 259 | FASN | 260 | FBP1 | 261 | FDFT1 |
| 262 | FFAR1 | 263 | FGF1 | 264 | FGF2 |
| 265 | FGFR1 | 266 | FGFR3 | 267 | FGR |
| 268 | FHIT | 269 | FKBP1A | 270 | FLT1 |
| 271 | FLT3 | 272 | FLT4 | 273 | FNTA FNTB |
| 274 | FOLH1 | 275 | FTO | 276 | FUCA1 |
| 277 | FUT7 | 278 | FYN | 279 | G6PD |
| 280 | GAA | 281 | GABBR1 | 282 | GABBR1 |
| 283 | GABBR2 | 284 | GABRA1 | 285 | GABRA3 |
| 286 | GABRA1 | 287 | GABRB2 | 288 | GABRB3 |
| 289 | GABRG2 | 290 | GABRG2 | 291 | GAPDH |
| 292 | GART | 293 | GBA | 294 | GCGR |
| 295 | GLO1 | 296 | GLRA1 | 297 | GLRA2 |
| 298 | GPBAR1 | 299 | GPR35 | 300 | GPR55 |
| 301 | GPR88 | 302 | GRIN1 | 303 | GRIN2A |
| 304 | GRK1 | 305 | GRK6 | 306 | GRK7 |
| 307 | GRM1 | 308 | GRM2 | 309 | GRM4 |
| 310 | GRM5 | 311 | GSK3A | 312 | GSK3B |
| 313 | GSR | 314 | GSTP1 | 315 | HAO2 |
| 316 | HCAR2 | 317 | HCRTR1 | 318 | HCRTR2 |
| 319 | HDAC1 | 320 | HDAC10 | 321 | HDAC11 |
| 322 | HDAC2 | 323 | HDAC3 | 324 | HDAC4 |
| 325 | HDAC5 | 326 | HDAC6 | 327 | HDAC7 |
| 328 | HDAC8 | 329 | HDAC9 | 330 | HIPK4 |
| 331 | HK1 | 332 | HK2 | 333 | HMGCR |
| 334 | HMOX1 | 335 | HNF4A | 336 | HPRT1 |
| 337 | HPSE | 338 | HRAS | 339 | HRH1 |
| 340 | HRH2 | 341 | HRH3 | 342 | HRH4 |
| 343 | HSD11B1 | 344 | HSD11B2 | 345 | HSD17B1 |
| 346 | HSD17B14 | 347 | HSD17B2 | 348 | HSD17B3 |
| 349 | HSP90AA1 | 350 | HSPA1A | 351 | HSPA5 |
| 352 | HSPA8 | 353 | HTR1A | 354 | HTR1B |
| 355 | HTR1D | 356 | HTR2A | 357 | HTR2B |
| 358 | HTR2C | 359 | HTR3A | 360 | HTR4 |
| 361 | HTR6 | 362 | HTR7 | 363 | HUNK |
| 364 | IARS | 365 | ICAM1 | 366 | ICK |
| 367 | IDH1 | 368 | IDO1 | 369 | IGF1R |
| 370 | IGFBP3 | 371 | IKBKB | 372 | IL2 |
| 373 | IL6ST | 374 | IMPDH1 | 375 | INMT |
| 376 | INSR | 377 | IRAK4 | 378 | ITGA2B |
| 379 | ITGAL | 380 | ITGAV | 381 | ITGAV |
| 382 | ITGB1 ITGA4 | 383 | ITGB5 | 384 | ITGB7 ITGA4 |
| 385 | ITK | 386 | JAK1 | 387 | JAK2 |
| 388 | JAK3 | 389 | JAK3 JAK1 | 390 | JUN |
| 391 | KCNA5 | 392 | KCNE1 | 393 | KCNH2 |
| 394 | KCNK2 | 395 | KCNMA1 | 396 | KDM1A |
| 397 | KDM2A | 398 | KDM3A | 399 | KDM4A |
| 400 | KDM4C | 401 | KDM4E | 402 | KDM5A |
| 403 | KDM5C | 404 | KDM6B | 405 | KDR |
| 406 | KIF11 | 407 | KIT | 408 | KLK1 |
| 409 | KLK2 | 410 | KMT2A | 411 | LARS |
| 412 | LCK | 413 | LDHA | 414 | LDHB |
| 415 | LGALS3 | 416 | LGALS4 | 417 | LGALS7 |
| 418 | LGALS8 | 419 | LGALS9 | 420 | LIPE |
| 421 | LRRK2 | 422 | LTA4H | 423 | LTB4R |
| 424 | LYN | 425 | MALT1 | 426 | MAOA |
| 427 | MAOB | 428 | MAP2K1 | 429 | MAP2K2 |
| 430 | MAP2K3 | 431 | MAP2K4 | 432 | MAP2K6 |
| 433 | MAP3K11 | 434 | MAP3K12 | 435 | MAP3K13 |
| 436 | MAP3K14 | 437 | MAP3K15 | 438 | MAP3K7 |
| 439 | MAP3K8 | 440 | MAP3K9 | 441 | MAPK1 |
| 442 | MAPK10 | 443 | MAPK14 | 444 | MAPK8 |
| 445 | MAPKAPK2 | 446 | MAPT | 447 | MARS |
| 448 | MAST1 | 449 | MB | 450 | MC1R |
| 451 | MCHR1 | 452 | MCL1 | 453 | MDM2 |
| 454 | MERTK | 455 | MET | 456 | METAP2 |
| 457 | MGLL | 458 | MGMT | 459 | MIF |
| 460 | MKNK1 | 461 | MME | 462 | MMP1 |
| 463 | MMP12 | 464 | MMP13 | 465 | MMP14 |
| 466 | MMP16 | 467 | MMP2 | 468 | MMP3 |
| 469 | MMP7 | 470 | MMP8 | 471 | MMP9 |
| 472 | MPG | 473 | MPI | 474 | MPO |
| 475 | MTAP | 476 | MTNR1A | 477 | MTNR1B |
| 478 | MTOR | 479 | MYLK | 480 | MYLK2 |
| 481 | NAAA | 482 | NADK | 483 | NAE1 |
| 484 | NAMPT | 485 | NAT1 | 486 | NCSTN |
| 487 | NEK2 | 488 | NEK6 | 489 | NEU4 |
| 490 | NFE2L2 | 491 | NMBR | 492 | NMT1 |
| 493 | NMUR2 | 494 | NOS1 | 495 | NOS2 |
| 496 | NOS3 | 497 | NOX4 | 498 | NPBWR1 |
| 499 | NPC1L1 | 500 | NPEPPS | 501 | NPY5R |
| 502 | NQO1 | 503 | NQO2 | 504 | NR1H2 |
| 505 | NR1H3 | 506 | NR1H4 | 507 | NR3C1 |
| 508 | NR3C2 | 509 | NR4A1 | 510 | NRAS |
| 511 | NRP1 | 512 | NTRK1 | 513 | NTRK2 |
| 514 | NUAK1 | 515 | ODC1 | 516 | OGA |
| 517 | OPRD1 | 518 | OPRK1 | 519 | OPRL1 |
| 520 | OPRM1 | 521 | OXSR1 | 522 | OXTR |
| 523 | P2RX3 | 524 | P2RX7 | 525 | P2RY1 |
| 526 | P2RY11 | 527 | PABPC1 | 528 | PAK1 |
| 529 | PARG | 530 | PARP1 | 531 | PARP2 |
| 532 | PDCD4 | 533 | PDE10A | 534 | PDE1B |
| 535 | PDE2A | 536 | PDE4B | 537 | PDE4D |
| 538 | PDE5A | 539 | PDE7A | 540 | PDE9A |
| 541 | PDGFRA | 542 | PDGFRB | 543 | PDPK1 |
| 544 | PEPD | 545 | PER2 | 546 | PFKFB3 |
| 547 | PGF | 548 | PGGT1B | 549 | PGR |
| 550 | PHKG2 | 551 | PHLPP2 | 552 | PIK3CA |
| 553 | PIK3CB | 554 | PIK3CG | 555 | PIK3R1 |
| 556 | PIM1 | 557 | PIM2 | 558 | PIM3 |
| 559 | PIN1 | 560 | PKN1 | 561 | PLA2G10 |
| 562 | PLA2G1B | 563 | PLA2G2A | 564 | PLA2G5 |
| 565 | PLA2G7 | 566 | PLAA | 567 | PLEC |
| 568 | PLG | 569 | PLK1 | 570 | PLK4 |
| 571 | PNMT | 572 | PNP | 573 | POLA1 |
| 574 | POLB | 575 | PPARA | 576 | PPARD |
| 577 | PPARG | 578 | PPIA | 579 | PPM1A |
| 580 | PPM1B | 581 | PPP1CA | 582 | PPP1CC |
| 583 | PPP2CA | 584 | PPP2R5A | 585 | PREP |
| 586 | PRKAA2 | 587 | PRKACA | 588 | PRKCA |
| 589 | PRKCB | 590 | PRKCD | 591 | PRKCE |
| 592 | PRKCG | 593 | PRKCH | 594 | PRKCZ |
| 595 | PRKD3 | 596 | PRKDC | 597 | PRMT1 |
| 598 | PRMT7 | 599 | PRPF4B | 600 | PRSS1 |
| 601 | PSEN2 | 602 | PTAFR | 603 | PTGDR |
| 604 | PTGDR2 | 605 | PTGER1 | 606 | PTGER2 |
| 607 | PTGER3 | 608 | PTGER4 | 609 | PTGES |
| 610 | PTGFR | 611 | PTGIR | 612 | PTGS1 |
| 613 | PTGS2 | 614 | PTK2 | 615 | PTPN1 |
| 616 | PTPN11 | 617 | PTPN2 | 618 | PTPN22 |
| 619 | PTPN6 | 620 | PTPRC | 621 | PTPRF |
| 622 | PTPRS | 623 | PYGB | 624 | PYGL |
| 625 | PYGM | 626 | QTRT1 | 627 | RAC1 |
| 628 | RAD51 | 629 | RAF1 | 630 | RARS |
| 631 | RBBP4 | 632 | RGS4 | 633 | RIPK2 |
| 634 | ROCK2 | 635 | RORA | 636 | RORB |
| 637 | RORC | 638 | RPS6KA1 | 639 | RPS6KA3 |
| 640 | RXRA | 641 | S1PR1 | 642 | S1PR2 |
| 643 | S1PR3 | 644 | S1PR4 | 645 | S1PR5 |
| 646 | SAE1 UBA2 | 647 | SBK1 | 648 | SCD |
| 649 | SCN2A | 650 | SCN5A | 651 | SCN9A |
| 652 | SELE | 653 | SELL | 654 | SELP |
| 655 | SERPINA6 | 656 | SERPINE1 | 657 | SETD2 |
| 658 | SETD7 | 659 | SETDB1 | 660 | SGPL1 |
| 661 | SHBG | 662 | SI | 663 | SIGMAR1 |
| 664 | SIRT2 | 665 | SLC15A1 | 666 | SLC22A12 |
| 667 | SLC22A6 | 668 | SLC28A2 | 669 | SLC28A3 |
| 670 | SLC29A1 | 671 | SLC37A4 | 672 | SLC5A1 |
| 673 | SLC5A11 | 674 | SLC5A2 | 675 | SLC5A4 |
| 676 | SLC6A2 | 677 | SLC6A3 | 678 | SLC6A4 |
| 679 | SLC7A5 | 680 | SMO | 681 | SMS |
| 682 | SMYD2 | 683 | SNCA | 684 | SNRK |
| 685 | SORD | 686 | SPHK1 | 687 | SPHK2 |
| 688 | SQLE | 689 | SRC | 690 | SRD5A2 |
| 691 | SRM | 692 | SSTR1 | 693 | SSTR2 |
| 694 | SSTR3 | 695 | SSTR4 | 696 | SSTR5 |
| 697 | ST6GAL1 | 698 | STAT3 | 699 | STK38 |
| 700 | STK39 | 701 | STS | 702 | SUV39H1 |
| 703 | SYK | 704 | TAAR1 | 705 | TACR1 |
| 706 | TACR3 | 707 | TARS | 708 | TAS2R31 |
| 709 | TBXAS1 | 710 | TDP1 | 711 | TDP2 |
| 712 | TEK | 713 | TERT | 714 | TGFBR1 |
| 715 | TGM2 | 716 | TH | 717 | THRA |
| 718 | THRB | 719 | TK1 | 720 | TLR4 |
| 721 | TLR9 | 722 | TMPRSS15 | 723 | TNF |
| 724 | TNKS | 725 | TNKS2 | 726 | TNNC1 |
| 727 | TNNI3 | 728 | TOP1 | 729 | TOP2A |
| 730 | TPMT | 731 | TRPA1 | 732 | TRPC3 |
| 733 | TRPC6 | 734 | TRPM8 | 735 | TRPV1 |
| 736 | TRPV3 | 737 | TSPO | 738 | TTR |
| 739 | TUBB1 | 740 | TXK | 741 | TYK2 |
| 742 | TYMP | 743 | TYMS | 744 | TYR |
| 745 | TYRO3 | 746 | UGT2B7 | 747 | UPP1 |
| 748 | UQCRB | 749 | VARS | 750 | VCP |
| 751 | VDR | 752 | VEGFA | 753 | WEE1 |
| 754 | WNT3 | 755 | XDH | 756 | YARS |
| 757 | YES1 | 758 | YWHAG |  |  |

**Supplementary Material 8:** Up-regulated genes in *A. baumannii* group from DEG analysis of GSE69528 (533 up-regulated genes with |log FC| > 1 *plus* adj.P.Val < 0.05).

| **No.** | **Gene.names** | **logFC** | **AveExpr** | **t** | **P.Value** | **adj.P.Val** | **B** | **Regulation** |
| --- | --- | --- | --- | --- | --- | --- | --- | --- |
| 1 | HP | -4.346809761 | 4.673913121 | -6.324412288 | 0.00E+00 | 5.80E-05 | 6.42010598 | Up-regulated |
| 2 | CD177 | -3.909780715 | 3.700293971 | -7.053899232 | 0.00E+00 | 1.40E-05 | 8.40431936 | Up-regulated |
| 3 | GPR84 | -3.511334565 | 4.021783181 | -8.30795789 | 0.00E+00 | 3.00E-06 | 11.67445185 | Up-regulated |
| 4 | C19orf59 | -3.505690977 | 8.066617554 | -7.785636488 | 7. 16E-09 | 5.00E-06 | 10.33700088 | Up-regulated |
| 5 | IFI27 | -3.351151721 | 7.241525601 | -2.651051773 | 0.012385644 | 0.048876494 | -3.296647027 | Up-regulated |
| 6 | ZDHHC19 | -3.236806457 | 4.54436217 | -6.724845085 | 0.00E+00 | 2.80E-05 | 7.515433258 | Up-regulated |
| 7 | MMP9 | -3.218284698 | 9.016564975 | -6.315793954 | 0.00E+00 | 5.80E-05 | 6.39638994 | Up-regulated |
| 8 | ANKRD22 | -3.104429027 | 5.286199944 | -7.679348633 | 0.00E+00 | 6.00E-06 | 10.0603798 | Up-regulated |
| 9 | CLEC4D | -2.975566001 | 5.243488522 | -6.679354912 | 0.00E+00 | 2.90E-05 | 7.391704825 | Up-regulated |
| 10 | ABP1 | -2.925909299 | 3.767487313 | -9.640054803 | 0.00E+00 | 1.00E-06 | 14.90970382 | Up-regulated |
| 11 | CLEC5A | -2.893630459 | 3.808529461 | -8.629410682 | 0.00E+00 | 2.00E-06 | 12.47872966 | Up-regulated |
| 12 | PGLYRP1 | -2.860170993 | 7.145799049 | -5.31642946 | 8.00E-06 | 0.000342696 | 3.621935921 | Up-regulated |
| 13 | MMP8 | -2.733363308 | 3.639747947 | -5.252751237 | 1.00E-05 | 0.000390011 | 3.444527767 | Up-regulated |
| 14 | PLSCR1 | -2.731607677 | 5.135809119 | -5.106405543 | 1.50E-05 | 0.000515644 | 3.037135624 | Up-regulated |
| 15 | ANXA3 | -2.633050574 | 7.053476135 | -4.351299855 | 0.00012989 | 0.002140569 | 0.956129204 | Up-regulated |
| 16 | RETN | -2.613953184 | 5.087259144 | -3.042534869 | 0.004665923 | 0.024192005 | -2.406460488 | Up-regulated |
| 17 | CA4 | -2.596331836 | 7.183425035 | -6.59235563 | 0.00E+00 | 3.40E-05 | 7.154546618 | Up-regulated |
| 18 | SERPING1 | -2.541672426 | 4.920033583 | -4.052886251 | 0.000302897 | 0.003807927 | 0.152563681 | Up-regulated |
| 19 | FCGR1B | -2.540199352 | 6.802244523 | -4.764219185 | 4.00E-05 | 0.001016019 | 2.088193335 | Up-regulated |
| 20 | FCGR1A | -2.538278297 | 7.377526242 | -4.734845826 | 4.30E-05 | 0.001066216 | 2.007093128 | Up-regulated |
| 21 | HIST1H4H | -2.444814945 | 6.003418802 | -4.627343787 | 5.90E-05 | 0.001281127 | 1.710921844 | Up-regulated |
| 22 | C1QB | -2.420682931 | 3.713066362 | -7.258552718 | 0.00E+00 | 1.00E-05 | 8.951301873 | Up-regulated |
| 23 | CEACAM1 | -2.411798255 | 6.937550634 | -5.139260896 | 1.30E-05 | 0.000488367 | 3.128544831 | Up-regulated |
| 24 | FCGR1C | -2.406301551 | 6.546684265 | -4.963034947 | 2.20E-05 | 0.000679267 | 2.638754512 | Up-regulated |
| 25 | INSL3 | -2.388468239 | 3.641847868 | -6.225880351 | 1.00E-06 | 6.90E-05 | 6.148651796 | Up-regulated |
| 26 | SOCS3 | -2.385588409 | 4.31070468 | -6.61545145 | 0.00E+00 | 3.30E-05 | 7.217571765 | Up-regulated |
| 27 | SLC2A14 | -2.384158445 | 4.351674313 | -4.988562148 | 2.10E-05 | 0.000643435 | 2.709617586 | Up-regulated |
| 28 | C5orf32 | -2.379241197 | 6.995332682 | -5.328262364 | 8.00E-06 | 0.000334575 | 3.65490837 | Up-regulated |
| 29 | PFKFB3 | -2.373869145 | 7.047741289 | -6.064337967 | 9. 11E-07 | 9.10E-05 | 5.702236595 | Up-regulated |
| 30 | OSM | -2.369291288 | 5.448781132 | -5.979491969 | 1. 16E-06 | 0.000105049 | 5.467170248 | Up-regulated |
| 31 | NLRC4 | -2.349678254 | 5.460043104 | -7.295188671 | 0.00E+00 | 1.00E-05 | 9.04871399 | Up-regulated |
| 32 | BPI | -2.339940716 | 4.569396324 | -3.34359299 | 0.002123533 | 0.013862163 | -1.677577996 | Up-regulated |
| 33 | VNN1 | -2.335091322 | 4.578905203 | -4.09958986 | 0.000265524 | 0.003483943 | 0.277299514 | Up-regulated |
| 34 | CBS | -2.333946557 | 4.044301917 | -5.446139418 | 5.00E-06 | 0.000278155 | 3.983421106 | Up-regulated |
| 35 | HPGD | -2.314871936 | 4.961048749 | -4.937438334 | 2.40E-05 | 0.00071291 | 2.567733349 | Up-regulated |
| 36 | CASP5 | -2.313490918 | 5.102750293 | -4.070862536 | 0.000287938 | 0.003689741 | 0.200525191 | Up-regulated |
| 37 | ARG1 | -2.313109755 | 5.686179775 | -3.776343927 | 0.000655557 | 0.00636352 | -0.576621926 | Up-regulated |
| 38 | LOC728744 | -2.302895457 | 4.721148572 | -4.935564453 | 2.40E-05 | 0.000715271 | 2.56253544 | Up-regulated |
| 39 | SIGLEC5 | -2.295731097 | 4.903666179 | -8.251753325 | 0.00E+00 | 3.00E-06 | 11.53232992 | Up-regulated |
| 40 | KRTAP19-6 | -2.290629087 | 3.623968647 | -5.214591064 | 1.10E-05 | 0.000424638 | 3.33824675 | Up-regulated |
| 41 | HNRNPA3P1 | -2.272505132 | 3.84752421 | -6.314889815 | 0.00E+00 | 5.80E-05 | 6.393901606 | Up-regulated |
| 42 | S100A12 | -2.248839434 | 8.811453868 | -4.409430033 | 0.000109996 | 0.00189096 | 1.114285218 | Up-regulated |
| 43 | F5 | -2.224395198 | 6.944863604 | -7.386666021 | 2. 14E-08 | 9.00E-06 | 9.291252765 | Up-regulated |
| 44 | SLPI | -2.217801135 | 5.254089515 | -3.559318217 | 0.001188709 | 0.009524703 | -1.135687601 | Up-regulated |
| 45 | GYG1 | -2.212332099 | 9.036402196 | -7.344496817 | 0.00E+00 | 9.00E-06 | 9.179571083 | Up-regulated |
| 46 | TCN1 | -2.17866545 | 6.268415559 | -3.892719208 | 0.000474505 | 0.005132182 | -0.271849033 | Up-regulated |
| 47 | CACNA1E | -2.173323238 | 3.718216877 | -6.648347058 | 0.00E+00 | 3.10E-05 | 7.307256701 | Up-regulated |
| 48 | LIN7A | -2.169151614 | 4.530893304 | -5.50190443 | 5.00E-06 | 0.000252217 | 4.138832399 | Up-regulated |
| 49 | AIM2 | -2.154630636 | 7.889405377 | -6.029257114 | 1.00E-06 | 9.50E-05 | 5.605090736 | Up-regulated |
| 50 | NAIP | -2.151524223 | 4.56866127 | -4.607238213 | 6.20E-05 | 0.001326464 | 1.655652296 | Up-regulated |
| 51 | TDRD9 | -2.130780777 | 4.482535861 | -4.727197026 | 4.40E-05 | 0.001084125 | 1.985986399 | Up-regulated |
| 52 | NTN3 | -2.108550693 | 3.736488667 | -5.983135441 | 1. 15E-06 | 0.000104916 | 5.477272111 | Up-regulated |
| 53 | LOC391045 | -2.098993023 | 4.412534539 | -3.96800945 | 0.000384451 | 0.004463591 | -0.07301883 | Up-regulated |
| 54 | RGL4 | -2.089146043 | 8.120271357 | -8.106224069 | 0.00E+00 | 4.00E-06 | 11.16229943 | Up-regulated |
| 55 | IFITM3 | -2.057752653 | 11.38425321 | -4.215877479 | 0.000191028 | 0.002734906 | 0.589612606 | Up-regulated |
| 56 | LMNB1 | -2.053506187 | 6.40628144 | -4.92051986 | 2.50E-05 | 0.000734667 | 2.520810888 | Up-regulated |
| 57 | MCTP2 | -2.052584967 | 5.427648829 | -3.102639309 | 0.003996571 | 0.021546862 | -2.263751156 | Up-regulated |
| 58 | RAB20 | -2.047907056 | 4.020701911 | -5.511715409 | 5.00E-06 | 0.000248662 | 4.166172541 | Up-regulated |
| 59 | SIPA1L2 | -2.044872977 | 6.859767929 | -4.488368594 | 8.80E-05 | 0.001661747 | 1.32977018 | Up-regulated |
| 60 | TLR5 | -2.034409861 | 7.719394392 | -5.473211865 | 5.00E-06 | 0.000265991 | 4.05887117 | Up-regulated |
| 61 | LILRA5 | -2.026217987 | 7.476236264 | -6.976737932 | 0.00E+00 | 1.70E-05 | 8.196886158 | Up-regulated |
| 62 | KREMEN1 | -2.008377106 | 4.01159169 | -4.618840216 | 6.00E-05 | 0.00130048 | 1.687540844 | Up-regulated |
| 63 | CST7 | -1.999148065 | 9.334762173 | -7.940375593 | 0.00E+00 | 4. 10E-06 | 10.73707074 | Up-regulated |
| 64 | DYSF | -1.997770141 | 9.511741275 | -6.133337411 | 1.00E-06 | 8.00E-05 | 5.893107769 | Up-regulated |
| 65 | CAMP | -1.995394765 | 8.541760366 | -3.359730687 | 0.002034217 | 0.013473182 | -1.637567657 | Up-regulated |
| 66 | SLC1A3 | -1.981566061 | 3.513692553 | -4.025266531 | 0.000327376 | 0.00402493 | 0.078996669 | Up-regulated |
| 67 | BMX | -1.972996962 | 3.961730319 | -6.446105909 | 0.00E+00 | 4.70E-05 | 6.754388292 | Up-regulated |
| 68 | ALPL | -1.962529039 | 10.71706135 | -5.037140804 | 1.80E-05 | 0.000591818 | 2.844558692 | Up-regulated |
| 69 | IL18R1 | -1.924746335 | 7.664861301 | -4.866220216 | 3.00E-05 | 0.000834003 | 2.370329831 | Up-regulated |
| 70 | LOC728519 | -1.914454713 | 6.461960768 | -3.917259905 | 0.000443096 | 0.004891243 | -0.207177711 | Up-regulated |
| 71 | EXOSC4 | -1.913387303 | 4.87564466 | -5.626313279 | 3.00E-06 | 0.000206856 | 4.485434371 | Up-regulated |
| 72 | ZNF438 | -1.913192759 | 6.323911439 | -7.961042963 | 0.00E+00 | 4. 10E-06 | 10.79026383 | Up-regulated |
| 73 | BST1 | -1.902814438 | 5.684649118 | -5.438755596 | 6.00E-06 | 0.000278155 | 3.962842252 | Up-regulated |
| 74 | ADM | -1.900498834 | 9.821415348 | -4.950920672 | 2.30E-05 | 0.00069445 | 2.605137417 | Up-regulated |
| 75 | TMEM176A | -1.898672816 | 5.073851685 | -3.549152233 | 0.001221988 | 0.00965814 | -1.161550847 | Up-regulated |
| 76 | LRG1 | -1.896968352 | 8.277398306 | -5.062989195 | 1.70E-05 | 0.000562306 | 2.916402785 | Up-regulated |
| 77 | BCL2A1 | -1.851311087 | 3.952069217 | -6.04329089 | 1.00E-06 | 9.30E-05 | 5.643961044 | Up-regulated |
| 78 | SYN2 | -1.84449949 | 3.657740069 | -7.007381989 | 6. 18E-08 | 1.60E-05 | 8.27934331 | Up-regulated |
| 79 | OPLAH | -1.843652298 | 4.103502667 | -7.336960094 | 0.00E+00 | 9.00E-06 | 9.159588306 | Up-regulated |
| 80 | LOC440731 | -1.828349114 | 5.489971428 | -5.619026031 | 3.00E-06 | 0.000206856 | 4.465138803 | Up-regulated |
| 81 | LOC654055 | -1.818221511 | 4.189192533 | -3.304255685 | 0.002357296 | 0.014898194 | -1.774729182 | Up-regulated |
| 82 | BAMBI | -1.817055014 | 4.187240236 | -3.811750409 | 0.000594328 | 0.006021885 | -0.484240117 | Up-regulated |
| 83 | MANSC1 | -1.816669604 | 5.475786008 | -4.736433963 | 4.30E-05 | 0.001066216 | 2.011476179 | Up-regulated |
| 84 | IRAK3 | -1.810621786 | 6.89747153 | -5.080573904 | 1.60E-05 | 0.00053991 | 2.965294003 | Up-regulated |
| 85 | TMEM176B | -1.807713925 | 4.177799299 | -4.585675803 | 6.60E-05 | 0.001396533 | 1.596423947 | Up-regulated |
| 86 | PFKFB2 | -1.805092238 | 3.782997282 | -5.333246875 | 8.00E-06 | 0.00033079 | 3.668798165 | Up-regulated |
| 87 | SLC26A8 | -1.800097669 | 4.208854575 | -7.199941057 | 0.00E+00 | 1. 14E-05 | 8.795133985 | Up-regulated |
| 88 | ABCA1 | -1.795160393 | 8.444897682 | -5.441596583 | 6.00E-06 | 0.000278155 | 3.970760149 | Up-regulated |
| 89 | LOC648984 | -1.786749776 | 6.688054195 | -4.318383119 | 0.000142687 | 0.002257096 | 0.866783963 | Up-regulated |
| 90 | SULT1B1 | -1.784954847 | 5.160552504 | -5.254125115 | 1.00E-05 | 0.000389591 | 3.448354729 | Up-regulated |
| 91 | IGFBP2 | -1.783202495 | 3.554542042 | -6.662998389 | 0.00E+00 | 3.00E-05 | 7.347169763 | Up-regulated |
| 92 | HIST1H3D | -1.776693238 | 4.317319872 | -6.229523854 | 1.00E-06 | 6.90E-05 | 6.158701487 | Up-regulated |
| 93 | METTL7B | -1.775116964 | 3.537535285 | -6.730314917 | 0.00E+00 | 2.80E-05 | 7.530297502 | Up-regulated |
| 94 | LILRA6 | -1.773701128 | 5.82053416 | -6.818618305 | 0.00E+00 | 2.40E-05 | 7.769862616 | Up-regulated |
| 95 | MRVI1 | -1.772996682 | 5.577904074 | -3.899527709 | 0.000465579 | 0.005071177 | -0.253920354 | Up-regulated |
| 96 | LOC652750 | -1.766936326 | 4.015843677 | -4.629306854 | 5.90E-05 | 0.00127789 | 1.71632042 | Up-regulated |
| 97 | LOC100170939 | -1.755874035 | 7.120535817 | -4.548803808 | 7.40E-05 | 0.001497193 | 1.495257289 | Up-regulated |
| 98 | DSC2 | -1.744383949 | 5.878366948 | -4.897239788 | 2.70E-05 | 0.000777834 | 2.456272328 | Up-regulated |
| 99 | TRPM2 | -1.743655257 | 3.528463786 | -7.472069528 | 0.00E+00 | 7.00E-06 | 9.516778558 | Up-regulated |
| 100 | GPR141 | -1.743103356 | 3.830094019 | -4.902059204 | 2.70E-05 | 0.000768633 | 2.469630356 | Up-regulated |
| 101 | MAPK14 | -1.738417343 | 4.245406786 | -4.979794763 | 2. 12E-05 | 0.000657085 | 2.685275723 | Up-regulated |
| 102 | SAMSN1 | -1.730792838 | 5.354751698 | -5.422091357 | 6.00E-06 | 0.000283856 | 3.916398442 | Up-regulated |
| 103 | LIMK2 | -1.726582578 | 5.337769015 | -4.970345016 | 2. 18E-05 | 0.000669404 | 2.659043712 | Up-regulated |
| 104 | ANKRD33 | -1.725022707 | 5.019353412 | -5.415194493 | 6.00E-06 | 0.000286081 | 3.89717663 | Up-regulated |
| 105 | GPR109A | -1.724100368 | 6.172252405 | -3.996666668 | 0.000354763 | 0.004231057 | 0.002980514 | Up-regulated |
| 106 | MGAM | -1.721056394 | 6.744417648 | -3.800445282 | 0.000613246 | 0.006111721 | -0.513770701 | Up-regulated |
| 107 | LOC100130904 | -1.720363722 | 3.519709196 | -6.302148721 | 0.00E+00 | 6.00E-05 | 6.358829846 | Up-regulated |
| 108 | IL1RN | -1.717504704 | 7.783845538 | -4.044956225 | 0.000309735 | 0.003855654 | 0.131426065 | Up-regulated |
| 109 | LOC642684 | -1.694363978 | 5.065036835 | -3.507507354 | 0.001367961 | 0.010401507 | -1.267175169 | Up-regulated |
| 110 | MYL9 | -1.691836734 | 4.736297565 | -3.157635837 | 0.003464949 | 0.019586775 | -2.131895122 | Up-regulated |
| 111 | OLR1 | -1.690425197 | 3.79538127 | -3.657349984 | 0.00090969 | 0.007970846 | -0.884749124 | Up-regulated |
| 112 | CR1 | -1.685699677 | 4.838559882 | -4.543430749 | 7.50E-05 | 0.001508687 | 1.480527523 | Up-regulated |
| 113 | SMARCD3 | -1.68002279 | 4.729787792 | -6.715567559 | 0.00E+00 | 2.80E-05 | 7.490215147 | Up-regulated |
| 114 | BATF | -1.677061427 | 6.269534623 | -6.219763565 | 1.00E-06 | 6.90E-05 | 6.131778191 | Up-regulated |
| 115 | ROPN1L | -1.672532966 | 6.767166814 | -5.428415595 | 6.00E-06 | 0.000281617 | 3.934024334 | Up-regulated |
| 116 | LOC650140 | -1.67234608 | 3.604644065 | -4.119061114 | 0.000251315 | 0.00335295 | 0.32942484 | Up-regulated |
| 117 | LOC653117 | -1.671263186 | 5.03433789 | -3.132719781 | 0.003696873 | 0.020399062 | -2.191780374 | Up-regulated |
| 118 | TLR2 | -1.665358121 | 5.130138795 | -4.242615605 | 0.000177052 | 0.002606604 | 0.661750833 | Up-regulated |
| 119 | NFIL3 | -1.661152369 | 8.153958537 | -5.967977658 | 1.00E-06 | 0.000106147 | 5.435241479 | Up-regulated |
| 120 | CCRL2 | -1.658271499 | 3.803566961 | -6.121010855 | 1.00E-06 | 8. 11E-05 | 5.859029582 | Up-regulated |
| 121 | LCN2 | -1.652162083 | 8.388357148 | -3.637795649 | 0.000959723 | 0.008264643 | -0.935021004 | Up-regulated |
| 122 | CDA | -1.650656119 | 7.596245104 | -4.273860339 | 0.000161991 | 0.002462899 | 0.746193666 | Up-regulated |
| 123 | NECAB2 | -1.639864289 | 3.849179036 | -4.812971004 | 3.40E-05 | 0.000924182 | 2.222947452 | Up-regulated |
| 124 | SLC37A3 | -1.639633456 | 4.675605712 | -5.370721087 | 7.00E-06 | 0.000306202 | 3.77323007 | Up-regulated |
| 125 | GPR27 | -1.638427383 | 4.018710165 | -3.998692551 | 0.000352751 | 0.004225137 | 0.008359648 | Up-regulated |
| 126 | KCNJ15 | -1.636268537 | 5.686623817 | -4.85312779 | 3.10E-05 | 0.000855563 | 2.334074793 | Up-regulated |
| 127 | LOC642103 | -1.631488061 | 7.769281001 | -3.47028445 | 0.001512571 | 0.01114602 | -1.361133126 | Up-regulated |
| 128 | SV2A | -1.629992235 | 4.211378917 | -5.879324776 | 2.00E-06 | 0.00012316 | 5.189204533 | Up-regulated |
| 129 | KLHDC8B | -1.623004152 | 5.693737777 | -4.442911632 | 1.00E-04 | 0.001784011 | 1.205585818 | Up-regulated |
| 130 | MOSC1 | -1.619564246 | 7.401125234 | -5.420192265 | 6.00E-06 | 0.000284212 | 3.911105602 | Up-regulated |
| 131 | TBC1D8 | -1.617643895 | 5.493581895 | -4.490930952 | 8.70E-05 | 0.001659048 | 1.336777874 | Up-regulated |
| 132 | HIST1H2BD | -1.617154641 | 7.029779213 | -3.895817401 | 0.000470423 | 0.005096064 | -0.263691925 | Up-regulated |
| 133 | HK3 | -1.614417281 | 8.182349748 | -4.103801769 | 0.000262385 | 0.003457634 | 0.288568999 | Up-regulated |
| 134 | KIF1B | -1.612280408 | 6.017676193 | -6.136392803 | 1.00E-06 | 8.00E-05 | 5.901553342 | Up-regulated |
| 135 | HIP1 | -1.607051825 | 5.79030198 | -3.298782567 | 0.002391713 | 0.015033932 | -1.788203042 | Up-regulated |
| 136 | LOC653610 | -1.606765209 | 4.743164952 | -4.247014321 | 0.000174851 | 0.002595333 | 0.673629507 | Up-regulated |
| 137 | LOC728093 | -1.60675194 | 3.55694991 | -6.495137348 | 0.00E+00 | 4.40E-05 | 6.888744306 | Up-regulated |
| 138 | FBXO6 | -1.59807505 | 6.285820469 | -6.271572815 | 1.00E-06 | 6.30E-05 | 6.274618036 | Up-regulated |
| 139 | FCAR | -1.596056721 | 6.264727742 | -4.303722074 | 0.000148779 | 0.002315039 | 0.827041185 | Up-regulated |
| 140 | TBC1D24 | -1.595546199 | 4.953512985 | -4.482367257 | 8.90E-05 | 0.001681384 | 1.31336046 | Up-regulated |
| 141 | GPR109B | -1.591610616 | 5.478277366 | -4.370157284 | 0.000123076 | 0.002069866 | 1.00738368 | Up-regulated |
| 142 | CARD17 | -1.589695822 | 3.698562947 | -8.953928108 | 0.00E+00 | 1. 12E-06 | 13.27562682 | Up-regulated |
| 143 | MCTP1 | -1.585212324 | 6.409503226 | -6.066005163 | 1.00E-06 | 9.10E-05 | 5.706851718 | Up-regulated |
| 144 | LOC100134379 | -1.584661077 | 3.641890616 | -3.338925718 | 0.002150059 | 0.013984977 | -1.689132907 | Up-regulated |
| 145 | PROK2 | -1.580509468 | 10.96398138 | -3.97798688 | 0.000373849 | 0.004381896 | -0.046578052 | Up-regulated |
| 146 | ECE1 | -1.578618969 | 4.686995522 | -6.049601375 | 1.00E-06 | 9.30E-05 | 5.661436172 | Up-regulated |
| 147 | IL1B | -1.578493483 | 8.555249863 | -4.227959849 | 0.000184583 | 0.002680479 | 0.622195775 | Up-regulated |
| 148 | OSTalpha | -1.574698683 | 3.48126766 | -5.196780148 | 1. 13E-05 | 0.000437855 | 3.28865189 | Up-regulated |
| 149 | ST3GAL4 | -1.574106724 | 4.804341847 | -4.750699123 | 4. 12E-05 | 0.001040304 | 2.050855538 | Up-regulated |
| 150 | DOK3 | -1.571588096 | 4.046628806 | -5.29026887 | 9.00E-06 | 0.000361283 | 3.549045143 | Up-regulated |
| 151 | IL18RAP | -1.569365658 | 9.771932809 | -3.805522357 | 0.000604678 | 0.006078174 | -0.500512524 | Up-regulated |
| 152 | OASL | -1.567427995 | 6.449118237 | -3.113547365 | 0.003885316 | 0.021192908 | -2.237694172 | Up-regulated |
| 153 | CDC20 | -1.565635852 | 4.813271505 | -4.170174636 | 0.00021747 | 0.003041096 | 0.46658527 | Up-regulated |
| 154 | CYP1B1 | -1.565451725 | 7.249033821 | -3.556173295 | 0.00119891 | 0.009557002 | -1.143691865 | Up-regulated |
| 155 | CD300C | -1.564709636 | 5.428382747 | -4.130470778 | 0.000243338 | 0.003299094 | 0.360001372 | Up-regulated |
| 156 | SAMD14 | -1.563889393 | 4.557766841 | -2.875549481 | 0.007126709 | 0.032966348 | -2.794865405 | Up-regulated |
| 157 | LOC648710 | -1.558117782 | 3.687829079 | -6.137208134 | 1.00E-06 | 8.00E-05 | 5.903806946 | Up-regulated |
| 158 | ETS2 | -1.556718213 | 7.533133038 | -4.981481542 | 2. 11E-05 | 0.000655332 | 2.689958613 | Up-regulated |
| 159 | TPST1 | -1.554010276 | 4.937750173 | -3.737267379 | 0.000730264 | 0.006856123 | -0.678215482 | Up-regulated |
| 160 | RGL1 | -1.550538112 | 4.766044006 | -7.155293926 | 0.00E+00 | 1.20E-05 | 8.675909923 | Up-regulated |
| 161 | MAP1A | -1.545388168 | 3.724155 | -4.650968091 | 5.50E-05 | 0.001233373 | 1.775915476 | Up-regulated |
| 162 | SLC2A11 | -1.539794044 | 5.742222922 | -2.704166974 | 0.010887504 | 0.04471959 | -3.18017202 | Up-regulated |
| 163 | IL1R2 | -1.539178115 | 6.718570323 | -3.701062146 | 0.000806827 | 0.007309152 | -0.771992661 | Up-regulated |
| 164 | XKR3 | -1.537728014 | 3.554266479 | -5.543944053 | 4. 11E-06 | 0.000233186 | 4.25597727 | Up-regulated |
| 165 | CCR1 | -1.537350603 | 7.871753281 | -4.055287638 | 0.000300856 | 0.003798188 | 0.15896705 | Up-regulated |
| 166 | FKBP9L | -1.532988179 | 3.916063751 | -5.288838291 | 9.00E-06 | 0.0003617 | 3.545059407 | Up-regulated |
| 167 | SIRPB1 | -1.531698498 | 5.213444682 | -4.721530856 | 4.50E-05 | 0.001090331 | 1.970353898 | Up-regulated |
| 168 | UGCG | -1.52652017 | 7.582485397 | -5.267689215 | 9. 19E-06 | 0.000378955 | 3.486139539 | Up-regulated |
| 169 | C19orf35 | -1.526363164 | 3.684937567 | -4.661641355 | 5.30E-05 | 0.001205682 | 1.805296709 | Up-regulated |
| 170 | QPCT | -1.512962708 | 9.545469585 | -3.54951786 | 0.001220776 | 0.009654031 | -1.160621192 | Up-regulated |
| 171 | SLC22A4 | -1.511863573 | 6.996501768 | -3.941521958 | 0.000414043 | 0.004691772 | -0.143109453 | Up-regulated |
| 172 | PHACTR1 | -1.509571811 | 4.071646627 | -4.282061276 | 0.000158252 | 0.002422947 | 0.76838318 | Up-regulated |
| 173 | FCER1G | -1.509391173 | 10.54741978 | -5.481332994 | 5.00E-06 | 0.000262651 | 4.081503875 | Up-regulated |
| 174 | TGFA | -1.508585271 | 5.423116464 | -3.85156805 | 0.000532116 | 0.005590631 | -0.379985763 | Up-regulated |
| 175 | GPR97 | -1.503150274 | 5.804176836 | -4.861588419 | 3.00E-05 | 0.000841593 | 2.357502338 | Up-regulated |
| 176 | LOC653867 | -1.500853461 | 3.72706095 | -6.274682452 | 0.00E+00 | 6.30E-05 | 6.283185561 | Up-regulated |
| 177 | HSPBL2 | -1.498336413 | 4.406392137 | -2.868394357 | 0.007255577 | 0.033363211 | -2.811230461 | Up-regulated |
| 178 | FUT7 | -1.49673487 | 4.521879703 | -3.938921265 | 0.000417066 | 0.004694378 | -0.149983204 | Up-regulated |
| 179 | RBPMS2 | -1.493926718 | 5.705187894 | -2.730736467 | 0.010203051 | 0.042679551 | -3.121373182 | Up-regulated |
| 180 | BEND7 | -1.493400059 | 4.173232899 | -4.412794974 | 0.000108942 | 0.001879783 | 1.123454385 | Up-regulated |
| 181 | JUNB | -1.487358663 | 6.748207538 | -5.495006905 | 5.00E-06 | 0.000256328 | 4.119610689 | Up-regulated |
| 182 | LOC400793 | -1.485325467 | 3.498582331 | -4.500477261 | 8.50E-05 | 0.001633724 | 1.362892631 | Up-regulated |
| 183 | DDAH2 | -1.485216962 | 5.818133068 | -5.365181878 | 7.00E-06 | 0.000308462 | 3.757792967 | Up-regulated |
| 184 | TNFAIP6 | -1.484812269 | 9.172559551 | -3.080099172 | 0.00423613 | 0.02251625 | -2.317442649 | Up-regulated |
| 185 | SPATS2L | -1.483875461 | 5.146863763 | -3.183511644 | 0.003238798 | 0.018651519 | -2.069447608 | Up-regulated |
| 186 | TECPR2 | -1.479561142 | 4.05351521 | -3.670761545 | 0.000876848 | 0.007766842 | -0.850208738 | Up-regulated |
| 187 | EPAS1 | -1.478197056 | 3.839134202 | -3.115320159 | 0.003867515 | 0.021112332 | -2.23345485 | Up-regulated |
| 188 | CKAP4 | -1.476524927 | 9.278847333 | -5.618963688 | 3.00E-06 | 0.000206856 | 4.464965169 | Up-regulated |
| 189 | BTNL8 | -1.474705515 | 5.541260638 | -2.789888957 | 0.00882075 | 0.038323121 | -2.989216063 | Up-regulated |
| 190 | CXCR1 | -1.472255401 | 9.062294251 | -5.221648075 | 1.00E-05 | 0.00041959 | 3.357899154 | Up-regulated |
| 191 | PECR | -1.470504922 | 5.030677021 | -5.009044589 | 2.00E-05 | 0.000622405 | 2.766499977 | Up-regulated |
| 192 | C20orf3 | -1.469781339 | 8.671380945 | -6.460938028 | 0.00E+00 | 4.60E-05 | 6.795051907 | Up-regulated |
| 193 | PPP1R3D | -1.468865928 | 6.159441999 | -6.039094566 | 1.00E-06 | 9.40E-05 | 5.632339301 | Up-regulated |
| 194 | CSTA | -1.467361276 | 4.457126982 | -3.747002456 | 0.000710913 | 0.006760947 | -0.652942012 | Up-regulated |
| 195 | FLOT1 | -1.466321108 | 8.029490264 | -5.042025176 | 1.80E-05 | 0.00058765 | 2.858132354 | Up-regulated |
| 196 | NTSR1 | -1.465672933 | 3.499004828 | -6.467616063 | 0.00E+00 | 4.60E-05 | 6.81335455 | Up-regulated |
| 197 | LOC100132317 | -1.460711113 | 3.636427381 | -5.114682442 | 1.40E-05 | 0.00050981 | 3.060159966 | Up-regulated |
| 198 | GM2A | -1.460342226 | 4.918251035 | -4.410504219 | 0.000109659 | 0.001889814 | 1.117212117 | Up-regulated |
| 199 | TMEM45B | -1.456969079 | 5.10906691 | -3.94133727 | 0.000414257 | 0.004691772 | -0.143597639 | Up-regulated |
| 200 | PLAUR | -1.456703775 | 6.727110573 | -6.377393378 | 0.00E+00 | 5.40E-05 | 6.565779668 | Up-regulated |
| 201 | GADD45A | -1.452842073 | 6.130146444 | -3.513984302 | 0.001344201 | 0.010291515 | -1.250782237 | Up-regulated |
| 202 | PPP1R3B | -1.445834067 | 4.135080183 | -2.915555631 | 0.006444939 | 0.0306883 | -2.702933641 | Up-regulated |
| 203 | METTL9 | -1.442610974 | 4.603177496 | -4.976490014 | 2. 14E-05 | 0.000661946 | 2.676101389 | Up-regulated |
| 204 | LOC100131360 | -1.438192361 | 5.440671702 | -5.44882843 | 5.00E-06 | 0.000277208 | 3.990915409 | Up-regulated |
| 205 | PSG3 | -1.43782892 | 5.854704745 | -3.72217495 | 0.000761281 | 0.007041767 | -0.717348894 | Up-regulated |
| 206 | LRRN1 | -1.436638547 | 3.808175055 | -4.931900367 | 2.40E-05 | 0.000716772 | 2.552372299 | Up-regulated |
| 207 | MARCO | -1.434057637 | 3.913974511 | -4.726539389 | 4.40E-05 | 0.001084125 | 1.984171891 | Up-regulated |
| 208 | DHRS13 | -1.430000414 | 5.85301313 | -4.059405604 | 0.000297386 | 0.003779542 | 0.169950347 | Up-regulated |
| 209 | LOC648997 | -1.428403295 | 3.774555733 | -4.293437876 | 0.000153205 | 0.002368047 | 0.799182377 | Up-regulated |
| 210 | C16orf7 | -1.423155578 | 7.107105054 | -5.520132841 | 4.00E-06 | 0.000245898 | 4.189628629 | Up-regulated |
| 211 | CARD16 | -1.419858627 | 4.873271937 | -7.607826339 | 1. 16E-08 | 6.00E-06 | 9.873420376 | Up-regulated |
| 212 | TRIB1 | -1.419589466 | 8.79331394 | -4.446144341 | 9.90E-05 | 0.001776655 | 1.214408741 | Up-regulated |
| 213 | POR | -1.410699443 | 5.509213137 | -4.944175667 | 2.40E-05 | 0.000702147 | 2.586423484 | Up-regulated |
| 214 | SQRDL | -1.41034167 | 6.545768305 | -8.00659293 | 0.00E+00 | 4.00E-06 | 10.90729609 | Up-regulated |
| 215 | C16orf57 | -1.410071832 | 8.635335898 | -7.466632813 | 0.00E+00 | 7.00E-06 | 9.502448311 | Up-regulated |
| 216 | ARHGEF11 | -1.408441806 | 4.514628335 | -5.756071294 | 2.00E-06 | 0.000159733 | 4.846613216 | Up-regulated |
| 217 | SH3GLB1 | -1.406780014 | 9.403656708 | -6.077733757 | 1.00E-06 | 8.90E-05 | 5.739314366 | Up-regulated |
| 218 | TGM3 | -1.406045246 | 5.61619864 | -3.362429631 | 0.002019636 | 0.013414844 | -1.630867447 | Up-regulated |
| 219 | ACSL1 | -1.403772103 | 9.887623734 | -3.440150398 | 0.001640334 | 0.011798941 | -1.436877451 | Up-regulated |
| 220 | PFTK1 | -1.401746457 | 4.978119721 | -3.978228185 | 0.000373596 | 0.004381896 | -0.045938318 | Up-regulated |
| 221 | LOC100130828 | -1.40100539 | 4.875905719 | -3.781308579 | 0.000646615 | 0.006326394 | -0.563686944 | Up-regulated |
| 222 | DRAM1 | -1.394177413 | 6.532864903 | -5.676352434 | 3.00E-06 | 0.000183322 | 4.624767354 | Up-regulated |
| 223 | FAM108C1 | -1.392419064 | 5.693090639 | -4.782449275 | 3.80E-05 | 0.000984707 | 2.138561571 | Up-regulated |
| 224 | FBN2 | -1.389503488 | 5.332850775 | -3.406831198 | 0.001793698 | 0.012423117 | -1.520286606 | Up-regulated |
| 225 | UPP1 | -1.388431677 | 8.994258755 | -4.818931458 | 3.40E-05 | 0.000919758 | 2.239434831 | Up-regulated |
| 226 | LILRB4 | -1.38566025 | 4.288313123 | -4.598788544 | 6.40E-05 | 0.001352952 | 1.63243671 | Up-regulated |
| 227 | TP53I11 | -1.380826754 | 3.71385305 | -7.697594807 | 9. 10E-09 | 6.00E-06 | 10.10797056 | Up-regulated |
| 228 | IFITM1 | -1.37889886 | 11.60230065 | -7.053476887 | 0.00E+00 | 1.40E-05 | 8.403185718 | Up-regulated |
| 229 | LOC100129827 | -1.376702767 | 3.558858261 | -5.691754715 | 3.00E-06 | 0.000178547 | 4.667643012 | Up-regulated |
| 230 | FOSL2 | -1.372543108 | 4.747957519 | -5.366327159 | 7.00E-06 | 0.000308462 | 3.760984711 | Up-regulated |
| 231 | CECR6 | -1.370363077 | 5.775302432 | -3.148553511 | 0.003547836 | 0.019905004 | -2.153752477 | Up-regulated |
| 232 | TMEM165 | -1.36710248 | 5.698265926 | -6.693859421 | 0.00E+00 | 2.90E-05 | 7.431176567 | Up-regulated |
| 233 | SCN9A | -1.365046818 | 3.510654419 | -7.616172245 | 1. 14E-08 | 6.00E-06 | 9.895270146 | Up-regulated |
| 234 | SERPINA1 | -1.363829708 | 7.433725559 | -5.467188232 | 5. 14E-06 | 0.000268204 | 4.042083741 | Up-regulated |
| 235 | SBNO2 | -1.363453586 | 6.861026976 | -4.600247563 | 6.40E-05 | 0.001349329 | 1.636444861 | Up-regulated |
| 236 | LOC651738 | -1.363405088 | 7.782683072 | -2.943646957 | 0.006003452 | 0.029153649 | -2.637951053 | Up-regulated |
| 237 | CES1 | -1.36174165 | 4.56250118 | -5.198990541 | 1. 12E-05 | 0.000436896 | 3.294806363 | Up-regulated |
| 238 | DNAJC25-GNG10 | -1.361227603 | 4.306036835 | -3.005580355 | 0.005128842 | 0.025843219 | -2.493458999 | Up-regulated |
| 239 | TLE3 | -1.360727813 | 5.810048408 | -4.544109574 | 7.50E-05 | 0.001508687 | 1.482388284 | Up-regulated |
| 240 | PGS1 | -1.358678927 | 8.82101987 | -5.188416419 | 1. 16E-05 | 0.000444331 | 3.265365561 | Up-regulated |
| 241 | LOC400499 | -1.356758969 | 4.480099211 | -3.591875706 | 0.001087925 | 0.008985697 | -1.052653448 | Up-regulated |
| 242 | CDCA5 | -1.356758307 | 4.155124752 | -4.368784667 | 0.000123559 | 0.0020755 | 1.003651217 | Up-regulated |
| 243 | TNFSF13B | -1.356487486 | 10.69273187 | -4.636032017 | 5.70E-05 | 0.001262814 | 1.734817991 | Up-regulated |
| 244 | EXT1 | -1.353250642 | 5.816147776 | -5.38629149 | 7.00E-06 | 0.000299277 | 3.816623733 | Up-regulated |
| 245 | C11orf82 | -1.347928708 | 5.224526485 | -5.257224922 | 9.00E-06 | 0.000388366 | 3.456989416 | Up-regulated |
| 246 | SIGLEC9 | -1.346841533 | 5.976584611 | -4.671461788 | 5. 18E-05 | 0.001191334 | 1.83233977 | Up-regulated |
| 247 | PHTF1 | -1.342224733 | 6.751147895 | -5.284143329 | 9.00E-06 | 0.000364484 | 3.531978981 | Up-regulated |
| 248 | ZAK | -1.340855813 | 4.790311523 | -4.462781673 | 9.40E-05 | 0.001725152 | 1.259837377 | Up-regulated |
| 249 | GPER | -1.340694368 | 3.554836553 | -5.841894155 | 2.00E-06 | 0.000133472 | 5.08522308 | Up-regulated |
| 250 | DDX60L | -1.34063144 | 7.662536151 | -3.51126135 | 0.001354141 | 0.010339229 | -1.257675504 | Up-regulated |
| 251 | IL4R | -1.340265838 | 6.565569781 | -3.953683077 | 0.00040019 | 0.004566926 | -0.110947643 | Up-regulated |
| 252 | HIST2H2AC | -1.33984302 | 8.837813742 | -4.710420503 | 4.60E-05 | 0.00111423 | 1.939709333 | Up-regulated |
| 253 | SORT1 | -1.33706994 | 6.942951672 | -4.550100619 | 7.40E-05 | 0.001495145 | 1.498812864 | Up-regulated |
| 254 | DGAT2 | -1.335658907 | 4.722643641 | -3.313012953 | 0.002303212 | 0.014684367 | -1.753148282 | Up-regulated |
| 255 | NBN | -1.33429379 | 5.899868294 | -7.681546281 | 0.00E+00 | 6.00E-06 | 10.06611411 | Up-regulated |
| 256 | NSUN7 | -1.330620709 | 5.079866586 | -3.402807804 | 0.001813125 | 0.01250796 | -1.530333876 | Up-regulated |
| 257 | MYBPC3 | -1.330105005 | 6.385406122 | -3.928152923 | 0.000429813 | 0.004804638 | -0.178428735 | Up-regulated |
| 258 | BCL2L11 | -1.32841555 | 3.860022145 | -4.276669248 | 0.000160701 | 0.002451298 | 0.753792636 | Up-regulated |
| 259 | GLT1D1 | -1.324165564 | 5.403961435 | -4.111179962 | 0.000256974 | 0.00340888 | 0.308318226 | Up-regulated |
| 260 | APOB48R | -1.323523561 | 6.614659679 | -4.643337501 | 5.60E-05 | 0.001254757 | 1.754916759 | Up-regulated |
| 261 | OSCAR | -1.323147827 | 7.957371293 | -5.28542377 | 9.00E-06 | 0.000364216 | 3.535546327 | Up-regulated |
| 262 | SOS2 | -1.323069242 | 5.127472013 | -4.627691603 | 5.90E-05 | 0.001281127 | 1.711878337 | Up-regulated |
| 263 | LTB4R | -1.319106266 | 6.751249832 | -5.13937889 | 1.30E-05 | 0.000488367 | 3.128873173 | Up-regulated |
| 264 | THBD | -1.317290668 | 3.858938615 | -3.234137637 | 0.002836414 | 0.016874672 | -1.946531932 | Up-regulated |
| 265 | WDFY3 | -1.314223476 | 4.952194216 | -4.323448265 | 0.000140639 | 0.002257096 | 0.880521869 | Up-regulated |
| 266 | CSF2RB | -1.312427245 | 4.749355481 | -2.764338524 | 0.009394844 | 0.040166487 | -3.046509903 | Up-regulated |
| 267 | PTPRJ | -1.308035247 | 4.831808552 | -3.892330127 | 0.00047502 | 0.005132182 | -0.272873275 | Up-regulated |
| 268 | SELL | -1.307450648 | 10.53997255 | -5.458940872 | 5.00E-06 | 0.00027101 | 4.019098689 | Up-regulated |
| 269 | HSPA1A | -1.305891343 | 9.174091413 | -3.638606342 | 0.000957597 | 0.008259513 | -0.932938916 | Up-regulated |
| 270 | AXUD1 | -1.303401452 | 8.653999519 | -5.548363874 | 4.00E-06 | 0.00023115 | 4.268292154 | Up-regulated |
| 271 | PADI4 | -1.302506404 | 10.36369 | -3.723839435 | 0.000757798 | 0.007034105 | -0.71303593 | Up-regulated |
| 272 | SLC24A4 | -1.30007907 | 5.698850687 | -4.319876861 | 0.00014208 | 0.002257096 | 0.870834958 | Up-regulated |
| 273 | FOXC1 | -1.299456675 | 3.915330966 | -4.439769846 | 0.00010084 | 0.001797846 | 1.197012329 | Up-regulated |
| 274 | GRAMD1A | -1.297378894 | 8.235465924 | -4.802134514 | 3.60E-05 | 0.000939007 | 2.192978972 | Up-regulated |
| 275 | VNN2 | -1.297174967 | 10.48521896 | -3.401405774 | 0.001819942 | 0.012542576 | -1.533833784 | Up-regulated |
| 276 | JMJD6 | -1.294903406 | 4.686743965 | -4.122002125 | 0.000249235 | 0.003344397 | 0.337304123 | Up-regulated |
| 277 | TREML2 | -1.292916613 | 6.235497793 | -4.363479524 | 0.000125447 | 0.002101639 | 0.989227793 | Up-regulated |
| 278 | CLEC4E | -1.292899494 | 5.246814844 | -3.794343239 | 0.0006237 | 0.00616274 | -0.529697105 | Up-regulated |
| 279 | SNX20 | -1.292226251 | 4.291379049 | -4.734980113 | 4.30E-05 | 0.001066216 | 2.007463736 | Up-regulated |
| 280 | INSC | -1.288040261 | 3.531059712 | -5.304537059 | 8.00E-06 | 0.000349759 | 3.588799267 | Up-regulated |
| 281 | FFAR2 | -1.28772207 | 10.42736149 | -4.110356816 | 0.000257572 | 0.003413568 | 0.306114407 | Up-regulated |
| 282 | PLXNC1 | -1.28720467 | 7.278244124 | -4.318048133 | 0.000142823 | 0.002257096 | 0.865875532 | Up-regulated |
| 283 | PIK3AP1 | -1.286464494 | 7.289211598 | -4.254867015 | 0.000170989 | 0.002557858 | 0.694843324 | Up-regulated |
| 284 | SLC2A3 | -1.285632399 | 11.95559917 | -6.754610131 | 0.00E+00 | 2.70E-05 | 7.596285152 | Up-regulated |
| 285 | LOC646434 | -1.285509676 | 5.279240542 | -4.00293819 | 0.000348572 | 0.00418947 | 0.01963543 | Up-regulated |
| 286 | LOC255809 | -1.285370499 | 6.257432657 | -4.849289357 | 3. 10E-05 | 0.000863399 | 2.323447728 | Up-regulated |
| 287 | LOC729891 | -1.285181467 | 3.551046674 | -4.453433707 | 9.70E-05 | 0.001751178 | 1.234308234 | Up-regulated |
| 288 | UBTD1 | -1.283899627 | 5.166936706 | -3.659305077 | 0.000904829 | 0.007946416 | -0.879717009 | Up-regulated |
| 289 | LY96 | -1.283298311 | 10.15002687 | -4.100033613 | 0.000265192 | 0.003483943 | 0.278486673 | Up-regulated |
| 290 | BCL3 | -1.283235417 | 8.522286923 | -4.753054131 | 4.10E-05 | 0.001036863 | 2.057358204 | Up-regulated |
| 291 | LOC400759 | -1.282595251 | 4.673788353 | -3.352101911 | 0.00207598 | 0.013671274 | -1.656492879 | Up-regulated |
| 292 | MARCKS | -1.281599284 | 10.53095037 | -5.606825923 | 3.00E-06 | 0.000208665 | 4.431158285 | Up-regulated |
| 293 | AGPAT9 | -1.278274229 | 8.837674707 | -5.703550002 | 3.00E-06 | 0.000174316 | 4.70047372 | Up-regulated |
| 294 | DKFZp761E198 | -1.276845257 | 5.030885214 | -4.224954437 | 0.000186166 | 0.002692769 | 0.614088672 | Up-regulated |
| 295 | BCL6 | -1.2748628 | 7.398819994 | -4.894795352 | 2.70E-05 | 0.000781747 | 2.449497602 | Up-regulated |
| 296 | WBP5 | -1.271613828 | 4.471871023 | -5.870256852 | 2.00E-06 | 0.000125474 | 5.164019199 | Up-regulated |
| 297 | UBXN2B | -1.268791284 | 7.701797015 | -4.13464924 | 0.000240479 | 0.003280592 | 0.371205072 | Up-regulated |
| 298 | SRPK1 | -1.268021678 | 8.186002323 | -5.763883245 | 2. 17E-06 | 0.000158683 | 4.868342776 | Up-regulated |
| 299 | ZMYND15 | -1.266403581 | 4.756027874 | -2.876341284 | 0.007112581 | 0.032911915 | -2.793052963 | Up-regulated |
| 300 | HIST2H2AA4 | -1.266227361 | 9.496008186 | -4.574983509 | 6.80E-05 | 0.001425122 | 1.567072107 | Up-regulated |
| 301 | MIR223 | -1.264027368 | 6.815433435 | -3.334017917 | 0.002178295 | 0.014093243 | -1.70127518 | Up-regulated |
| 302 | GADD45B | -1.262369099 | 7.173059146 | -7.804593565 | 0.00E+00 | 5.00E-06 | 10.38618338 | Up-regulated |
| 303 | CETP | -1.260482681 | 3.771544593 | -4.818591191 | 3.40E-05 | 0.000919758 | 2.238493539 | Up-regulated |
| 304 | IDI1 | -1.257151708 | 6.726801741 | -5.713025034 | 3.00E-06 | 0.000173979 | 4.726843612 | Up-regulated |
| 305 | ACSL4 | -1.256504181 | 6.087980093 | -5.160970225 | 1.30E-05 | 0.000473162 | 3.188962648 | Up-regulated |
| 306 | TIMM10 | -1.255065429 | 7.603264497 | -3.316258507 | 0.002283472 | 0.014610446 | -1.745143267 | Up-regulated |
| 307 | LOC100134703 | -1.253339637 | 5.391903992 | -3.492179111 | 0.001425818 | 0.010731714 | -1.305918933 | Up-regulated |
| 308 | TP53I3 | -1.252627961 | 3.962149046 | -3.305416227 | 0.002350059 | 0.014879358 | -1.771870767 | Up-regulated |
| 309 | G0S2 | -1.25223901 | 3.454965336 | -5.127390586 | 1.40E-05 | 0.000499453 | 3.095515492 | Up-regulated |
| 310 | RRAGD | -1.251626114 | 8.364108492 | -5.621277921 | 3.00E-06 | 0.000206856 | 4.471410605 | Up-regulated |
| 311 | CXCL1 | -1.250747734 | 4.765029109 | -3.146994322 | 0.003562254 | 0.019962841 | -2.157501551 | Up-regulated |
| 312 | FLJ22662 | -1.250607116 | 10.19263039 | -4.957480389 | 2.30E-05 | 0.000685801 | 2.623339643 | Up-regulated |
| 313 | BRI3P1 | -1.250389129 | 8.109148252 | -4.727034438 | 4.40E-05 | 0.001084125 | 1.985537794 | Up-regulated |
| 314 | PADI2 | -1.249306037 | 4.425727485 | -3.914200184 | 0.000446898 | 0.004915098 | -0.21524824 | Up-regulated |
| 315 | STK3 | -1.246125073 | 6.149606949 | -4.260331723 | 0.000168351 | 0.002523811 | 0.709611871 | Up-regulated |
| 316 | CD36 | -1.245610793 | 7.81672082 | -4.16407958 | 0.000221258 | 0.003074429 | 0.450205138 | Up-regulated |
| 317 | TMEM88 | -1.245182459 | 4.05930961 | -3.028262525 | 0.00483981 | 0.024816709 | -2.440128718 | Up-regulated |
| 318 | HIST1H2BE | -1.240652738 | 4.830757225 | -3.410946385 | 0.001774035 | 0.012335955 | -1.51000458 | Up-regulated |
| 319 | NEDD4 | -1.240180248 | 3.680511998 | -4.002547225 | 0.000348954 | 0.00419046 | 0.01859693 | Up-regulated |
| 320 | IFNAR1 | -1.240112518 | 8.764482698 | -6.222241213 | 1.00E-06 | 6.90E-05 | 6.138613263 | Up-regulated |
| 321 | IRF7 | -1.239404129 | 8.293520652 | -3.661376008 | 0.000899708 | 0.00791902 | -0.874385597 | Up-regulated |
| 322 | B4GALT5 | -1.239334809 | 9.811796504 | -5.085295095 | 1.60E-05 | 0.000537434 | 2.97842249 | Up-regulated |
| 323 | CXCL16 | -1.239071126 | 8.256165292 | -4.414490755 | 0.000108414 | 0.001875319 | 1.128075809 | Up-regulated |
| 324 | FBXL5 | -1.238969183 | 7.643112532 | -4.962125539 | 2.20E-05 | 0.000679575 | 2.636230634 | Up-regulated |
| 325 | KLHL2 | -1.238203214 | 7.108946177 | -3.473756819 | 0.001498481 | 0.011088457 | -1.35238651 | Up-regulated |
| 326 | LOC100133740 | -1.23777343 | 3.944586904 | -4.614993811 | 6.10E-05 | 0.001303118 | 1.676967341 | Up-regulated |
| 327 | LOC644237 | -1.236425807 | 6.499925574 | -6.851012067 | 0.00E+00 | 2.20E-05 | 7.857553993 | Up-regulated |
| 328 | TMCO3 | -1.236088489 | 6.556241935 | -6.053678675 | 1.00E-06 | 9.20E-05 | 5.672725968 | Up-regulated |
| 329 | MPP7 | -1.233826014 | 3.632505948 | -4.842957952 | 3. 15E-05 | 0.000863831 | 2.305920819 | Up-regulated |
| 330 | TLR4 | -1.233524858 | 7.23254155 | -3.38875639 | 0.001882573 | 0.012827531 | -1.565381099 | Up-regulated |
| 331 | TIFA | -1.231806272 | 4.046672216 | -5.032658019 | 1.80E-05 | 0.000596751 | 2.832101954 | Up-regulated |
| 332 | RHD | -1.231790021 | 3.798851667 | -3.030428579 | 0.00481303 | 0.024715753 | -2.435024529 | Up-regulated |
| 333 | CTSD | -1.231373592 | 6.859228637 | -5.680936453 | 3.00E-06 | 0.000181752 | 4.637528601 | Up-regulated |
| 334 | SERPINB8 | -1.229236143 | 4.689791979 | -5.293901504 | 9.00E-06 | 0.000358747 | 3.559166152 | Up-regulated |
| 335 | ASGR2 | -1.228982157 | 5.342925738 | -3.45989567 | 0.001555495 | 0.011348355 | -1.38727885 | Up-regulated |
| 336 | SIRPD | -1.227263025 | 4.612311397 | -3.289161038 | 0.002453387 | 0.015263332 | -1.811863744 | Up-regulated |
| 337 | LOC100128269 | -1.22724418 | 5.903233913 | -3.824093238 | 0.000574321 | 0.005886787 | -0.4519635 | Up-regulated |
| 338 | MERTK | -1.22710053 | 3.890513736 | -4.740404064 | 4.20E-05 | 0.001062187 | 2.022434062 | Up-regulated |
| 339 | LOC642956 | -1.226468213 | 4.060082803 | -5.379738867 | 7.00E-06 | 0.000302175 | 3.798361878 | Up-regulated |
| 340 | TSHZ3 | -1.226245561 | 5.81501591 | -4.808317055 | 3.50E-05 | 0.000928973 | 2.210075828 | Up-regulated |
| 341 | GK | -1.224870614 | 7.382829938 | -3.195207002 | 0.003141267 | 0.018248145 | -2.041138234 | Up-regulated |
| 342 | KIAA0040 | -1.221470846 | 6.056453465 | -5.31611741 | 8.00E-06 | 0.000342696 | 3.621066412 | Up-regulated |
| 343 | PGD | -1.221354224 | 10.03833045 | -3.971323224 | 0.000380897 | 0.00444019 | -0.064239462 | Up-regulated |
| 344 | LOC729010 | -1.219838874 | 4.650280506 | -3.146108931 | 0.003570467 | 0.019974924 | -2.159630053 | Up-regulated |
| 345 | FABP5 | -1.217531482 | 5.009794515 | -4.548105545 | 7.40E-05 | 0.001497193 | 1.49334288 | Up-regulated |
| 346 | STAB1 | -1.217186466 | 4.523141666 | -3.124069252 | 0.003780795 | 0.020752695 | -2.212514603 | Up-regulated |
| 347 | HIST2H2AA3 | -1.213503216 | 9.062728695 | -4.054576912 | 0.000301458 | 0.003798188 | 0.157071763 | Up-regulated |
| 348 | FAM20A | -1.213311395 | 3.439345327 | -7.124025065 | 0.00E+00 | 1.30E-05 | 8.592276652 | Up-regulated |
| 349 | PPAP2C | -1.212332054 | 3.935249404 | -6.198029713 | 6. 19E-07 | 7.30E-05 | 6.071803826 | Up-regulated |
| 350 | AQP9 | -1.210665923 | 11.74429732 | -5.314797421 | 8.00E-06 | 0.000342696 | 3.617388354 | Up-regulated |
| 351 | UBE2C | -1.209364488 | 4.38220277 | -4.745328377 | 4. 18E-05 | 0.001054672 | 2.036027483 | Up-regulated |
| 352 | RTP4 | -1.20835982 | 5.384553918 | -2.853939092 | 0.007522633 | 0.034218776 | -2.844220159 | Up-regulated |
| 353 | FAM160B1 | -1.20787966 | 6.644865602 | -5.953730353 | 1.00E-06 | 0.000108887 | 5.395725364 | Up-regulated |
| 354 | LOC651612 | -1.207861964 | 5.823230412 | -2.966502826 | 0.005665456 | 0.027811473 | -2.584821929 | Up-regulated |
| 355 | SERPINB1 | -1.205389187 | 9.674734334 | -5.255364922 | 1.00E-05 | 0.000389328 | 3.451808259 | Up-regulated |
| 356 | LOC441009 | -1.20454077 | 5.812918455 | -4.562152668 | 7. 10E-05 | 0.001452675 | 1.531865851 | Up-regulated |
| 357 | DIRC2 | -1.203652457 | 6.906647668 | -4.821711256 | 3.40E-05 | 0.000915054 | 2.247124984 | Up-regulated |
| 358 | LOC440459 | -1.203641488 | 3.857958269 | -5.671433789 | 3.00E-06 | 0.000184229 | 4.611073993 | Up-regulated |
| 359 | MXD3 | -1.203586258 | 5.171035442 | -4.130971897 | 0.000242994 | 0.00329875 | 0.361344855 | Up-regulated |
| 360 | ATP9A | -1.202882497 | 7.319714996 | -2.65115881 | 0.012382442 | 0.048876494 | -3.296413762 | Up-regulated |
| 361 | ENTPD1 | -1.201029204 | 6.685565037 | -5.569353494 | 4.00E-06 | 0.000220168 | 4.3267718 | Up-regulated |
| 362 | TBKBP1 | -1.20083754 | 3.539516804 | -9.08930635 | 0.00E+00 | 1.00E-06 | 13.60353211 | Up-regulated |
| 363 | EGLN1 | -1.200729306 | 6.086938724 | -3.146974225 | 0.003562441 | 0.019962841 | -2.157549869 | Up-regulated |
| 364 | SLA | -1.200017704 | 8.923517606 | -6.397506598 | 0.00E+00 | 5. 18E-05 | 6.621026363 | Up-regulated |
| 365 | LOC728323 | -1.197480917 | 4.057908171 | -2.854895502 | 0.007504682 | 0.034148264 | -2.842040439 | Up-regulated |
| 366 | KIAA1881 | -1.19709354 | 5.713138782 | -3.241031267 | 0.002785466 | 0.016667369 | -1.929720627 | Up-regulated |
| 367 | C19orf38 | -1.194476108 | 4.559145726 | -4.666945579 | 5.30E-05 | 0.001202411 | 1.819902121 | Up-regulated |
| 368 | LOC644132 | -1.192199273 | 5.822097813 | -4.621470345 | 6.00E-05 | 0.001296779 | 1.694771745 | Up-regulated |
| 369 | GNG5 | -1.188818603 | 6.274326085 | -3.830101323 | 0.000564822 | 0.005811624 | -0.436239034 | Up-regulated |
| 370 | RAB32 | -1.18856785 | 8.359273722 | -4.657370852 | 5.40E-05 | 0.001216679 | 1.793539621 | Up-regulated |
| 371 | GPR42 | -1.188277969 | 3.47120089 | -6.163460542 | 1.00E-06 | 7.70E-05 | 5.976347809 | Up-regulated |
| 372 | ERI1 | -1.186762321 | 6.268836012 | -4.330437421 | 0.000137862 | 0.002225294 | 0.899484371 | Up-regulated |
| 373 | FAM20C | -1.185455729 | 4.050900243 | -3.576361621 | 0.001134874 | 0.009215147 | -1.092259007 | Up-regulated |
| 374 | CASP4 | -1.184513607 | 7.508446303 | -3.469752886 | 0.00151474 | 0.011150414 | -1.362471758 | Up-regulated |
| 375 | CEACAM4 | -1.183372513 | 4.400913923 | -3.888187366 | 0.000480538 | 0.005165509 | -0.28377682 | Up-regulated |
| 376 | WDR51A | -1.1831444 | 4.399457375 | -3.548627506 | 0.00122373 | 0.009659642 | -1.16288497 | Up-regulated |
| 377 | HIST1H2BC | -1.182063938 | 6.120683336 | -3.846000545 | 0.000540417 | 0.005641623 | -0.394585802 | Up-regulated |
| 378 | ZNF319 | -1.181776661 | 7.762188534 | -6.624974692 | 0.00E+00 | 3.20E-05 | 7.243545476 | Up-regulated |
| 379 | DTX3L | -1.179597728 | 4.888737075 | -2.964323067 | 0.005696898 | 0.027937445 | -2.589898716 | Up-regulated |
| 380 | FKBP5 | -1.179225822 | 9.369274621 | -4.713496847 | 4.60E-05 | 0.001108199 | 1.948193434 | Up-regulated |
| 381 | HELB | -1.176849137 | 4.262615709 | -3.657157717 | 0.00091017 | 0.007970846 | -0.885243935 | Up-regulated |
| 382 | C7orf53 | -1.176706995 | 5.424184902 | -2.911601768 | 0.00650948 | 0.030900639 | -2.712051648 | Up-regulated |
| 383 | MYOF | -1.171566526 | 4.976462432 | -3.475228333 | 0.001492548 | 0.011064034 | -1.348678737 | Up-regulated |
| 384 | IL1R1 | -1.167368519 | 3.652292893 | -4.080880246 | 0.000279919 | 0.003611075 | 0.227279913 | Up-regulated |
| 385 | BASP1 | -1.167106202 | 12.1975612 | -5.578153772 | 4.00E-06 | 0.000217801 | 4.35128862 | Up-regulated |
| 386 | EIF4E3 | -1.166678457 | 8.308682125 | -3.934811749 | 0.000421887 | 0.004739679 | -0.160841842 | Up-regulated |
| 387 | ECM1 | -1.165391061 | 4.018998206 | -4.280863423 | 0.000158792 | 0.002424846 | 0.765141463 | Up-regulated |
| 388 | CD163 | -1.164029772 | 6.435095021 | -4.057449132 | 0.00029903 | 0.003792187 | 0.164731702 | Up-regulated |
| 389 | HIST1H2AM | -1.162358342 | 3.808375419 | -4.427643018 | 0.000104406 | 0.001842567 | 1.16393186 | Up-regulated |
| 390 | RABGEF1 | -1.161167611 | 4.602972401 | -4.678191915 | 5.10E-05 | 0.00117618 | 1.850878061 | Up-regulated |
| 391 | AGTRAP | -1.159799128 | 6.563336931 | -5.520879826 | 4.00E-06 | 0.000245898 | 4.191710152 | Up-regulated |
| 392 | TRIM25 | -1.159264068 | 8.83177837 | -4.240363584 | 0.000178189 | 0.002615122 | 0.655670487 | Up-regulated |
| 393 | ADAP1 | -1.158784945 | 4.526919877 | -3.289493907 | 0.002451229 | 0.015256709 | -1.811045723 | Up-regulated |
| 394 | LOC730278 | -1.157231757 | 10.11252807 | -5.011995874 | 1.90E-05 | 0.000619553 | 2.774697727 | Up-regulated |
| 395 | C1QC | -1.156230582 | 3.485973343 | -8.690522174 | 0.00E+00 | 2.00E-06 | 12.62996229 | Up-regulated |
| 396 | LOC649071 | -1.15475883 | 3.530404103 | -4.699389463 | 4.80E-05 | 0.001132695 | 1.909294177 | Up-regulated |
| 397 | LOC653907 | -1.153925212 | 5.746491899 | -2.842676607 | 0.00773707 | 0.034898167 | -2.869856068 | Up-regulated |
| 398 | C4orf18 | -1.153125105 | 5.638392472 | -2.917536223 | 0.006412836 | 0.030566755 | -2.698363557 | Up-regulated |
| 399 | TOP2A | -1.151933867 | 3.871107273 | -3.703127502 | 0.000802257 | 0.007281948 | -0.766652338 | Up-regulated |
| 400 | GBA | -1.151276065 | 7.090393851 | -5.910433086 | 1.00E-06 | 0.00011534 | 5.275578803 | Up-regulated |
| 401 | LOC100132287 | -1.151214223 | 6.002494695 | -2.865664012 | 0.007305327 | 0.03347261 | -2.817469035 | Up-regulated |
| 402 | S100A11 | -1.148170783 | 9.590632688 | -5.711066113 | 3.00E-06 | 0.000173979 | 4.721391953 | Up-regulated |
| 403 | FGD4 | -1.147726665 | 3.432998417 | -3.737836085 | 0.00072912 | 0.006855626 | -0.676739722 | Up-regulated |
| 404 | STOM | -1.146649171 | 8.76564955 | -3.744879043 | 0.000715091 | 0.00677296 | -0.658456732 | Up-regulated |
| 405 | GPR160 | -1.143908168 | 5.570292077 | -3.27558896 | 0.002542981 | 0.015646178 | -1.845183091 | Up-regulated |
| 406 | SP100 | -1.142866079 | 6.71008394 | -4.117823478 | 0.000252195 | 0.003361481 | 0.326109554 | Up-regulated |
| 407 | SLC22A15 | -1.142773107 | 5.703349156 | -3.009778375 | 0.005074145 | 0.025678305 | -2.483605077 | Up-regulated |
| 408 | SESN2 | -1.142050872 | 3.74433028 | -4.649609011 | 5.50E-05 | 0.001236227 | 1.772174995 | Up-regulated |
| 409 | HIST2H2AB | -1.139988401 | 3.489895615 | -5.611738779 | 3.00E-06 | 0.000207712 | 4.444842213 | Up-regulated |
| 410 | LOC642334 | -1.139963723 | 4.999530662 | -2.982130631 | 0.005444781 | 0.027072448 | -2.548363325 | Up-regulated |
| 411 | ZDHHC3 | -1.138444371 | 5.441437528 | -5.089688149 | 1.50E-05 | 0.000531931 | 2.990639264 | Up-regulated |
| 412 | PARP9 | -1.13690298 | 8.501516238 | -3.523314544 | 0.001310673 | 0.010118162 | -1.227145159 | Up-regulated |
| 413 | C3AR1 | -1.136822883 | 5.373490056 | -2.971336512 | 0.005596314 | 0.027574252 | -2.573556601 | Up-regulated |
| 414 | LOC100134728 | -1.134311918 | 6.56820488 | -3.407275668 | 0.001791564 | 0.012423117 | -1.519176342 | Up-regulated |
| 415 | PDSS1 | -1.133668716 | 4.827446951 | -3.392018331 | 0.001866227 | 0.012754378 | -1.557250982 | Up-regulated |
| 416 | KDM6B | -1.131274106 | 6.052903436 | -4.191418574 | 0.00020476 | 0.002895292 | 0.523727531 | Up-regulated |
| 417 | PROK1 | -1.130508267 | 3.929012467 | -4.641740855 | 5.60E-05 | 0.001255781 | 1.750523638 | Up-regulated |
| 418 | SOD2 | -1.127793275 | 8.26979962 | -4.04014472 | 0.000313958 | 0.003897778 | 0.118606955 | Up-regulated |
| 419 | PYGL | -1.122997221 | 10.0828366 | -4.056860488 | 0.000299526 | 0.003792905 | 0.163161712 | Up-regulated |
| 420 | ITGAM | -1.120088428 | 9.213764936 | -3.660852526 | 0.000901 | 0.007925388 | -0.875733361 | Up-regulated |
| 421 | OAS1 | -1.119544965 | 6.178714546 | -2.681471528 | 0.01150575 | 0.046501294 | -3.230116322 | Up-regulated |
| 422 | LOC442132 | -1.118520397 | 3.626007671 | -4.51513118 | 8. 12E-05 | 0.001595984 | 1.403000705 | Up-regulated |
| 423 | CNIH4 | -1.118149922 | 8.501059178 | -5.399349027 | 6.00E-06 | 0.000296067 | 3.853014945 | Up-regulated |
| 424 | HSPA1B | -1.10727986 | 7.962423552 | -4.932778576 | 2.40E-05 | 0.000716772 | 2.554808135 | Up-regulated |
| 425 | MSL3 | -1.106141301 | 6.658759471 | -7.695769272 | 9. 15E-09 | 6.00E-06 | 10.10321102 | Up-regulated |
| 426 | MSRA | -1.104603257 | 6.585745767 | -4.78019807 | 3.80E-05 | 0.000988397 | 2.132340276 | Up-regulated |
| 427 | LOC554208 | -1.10436438 | 4.005657968 | -4.479477634 | 9.00E-05 | 0.001693098 | 1.305460797 | Up-regulated |
| 428 | HAUS4 | -1.103527785 | 7.252513769 | -4.099114251 | 0.000265881 | 0.003483943 | 0.27602717 | Up-regulated |
| 429 | LOC651524 | -1.102826516 | 3.8618279 | -4.141072974 | 0.000236149 | 0.003231 | 0.388435158 | Up-regulated |
| 430 | ENTPD7 | -1.101852393 | 3.454073698 | -3.601140456 | 0.001060791 | 0.008845427 | -1.028968458 | Up-regulated |
| 431 | TRPM6 | -1.101244674 | 5.926015878 | -2.65683561 | 0.012213683 | 0.048417165 | -3.284033884 | Up-regulated |
| 432 | FABP5L2 | -1.100379384 | 4.81685668 | -4.815824673 | 3.40E-05 | 0.000923553 | 2.230840745 | Up-regulated |
| 433 | CCDC17 | -1.100140387 | 3.813075425 | -3.377852806 | 0.001938218 | 0.013063609 | -1.592531509 | Up-regulated |
| 434 | KCNJ2 | -1.099388773 | 8.592826958 | -3.353672987 | 0.002067313 | 0.013647004 | -1.652597031 | Up-regulated |
| 435 | HIST2H2BE | -1.098968824 | 9.037181983 | -3.509458609 | 0.001360761 | 0.010378406 | -1.262237966 | Up-regulated |
| 436 | LOC728440 | -1.097626202 | 6.095042695 | -4.024884539 | 0.000327727 | 0.004025705 | 0.077980271 | Up-regulated |
| 437 | LOC100132119 | -1.09676418 | 6.150505079 | -3.066853915 | 0.004383198 | 0.023107368 | -2.348896898 | Up-regulated |
| 438 | IL8RB | -1.096214455 | 11.05609117 | -4.017090854 | 0.000334986 | 0.004082495 | 0.057249405 | Up-regulated |
| 439 | EPHB4 | -1.095495909 | 3.4940291 | -4.518459148 | 8.10E-05 | 0.001586582 | 1.41211292 | Up-regulated |
| 440 | GRN | -1.093708903 | 8.645478693 | -4.846767154 | 3. 12E-05 | 0.000863831 | 2.316465312 | Up-regulated |
| 441 | LOC728417 | -1.092495795 | 6.84400317 | -3.382836976 | 0.001912589 | 0.012956906 | -1.580125655 | Up-regulated |
| 442 | MT1X | -1.090769942 | 7.686590463 | -5.985262036 | 1. 14E-06 | 0.000104916 | 5.483167989 | Up-regulated |
| 443 | LDLR | -1.089279229 | 7.27848157 | -4.378771267 | 0.000120081 | 0.002031765 | 1.03081308 | Up-regulated |
| 444 | ICAM1 | -1.087580449 | 4.45558876 | -3.106308053 | 0.003958818 | 0.021417867 | -2.254992647 | Up-regulated |
| 445 | RIT1 | -1.087546063 | 6.301259259 | -4.843544177 | 3. 15E-05 | 0.000863831 | 2.307543521 | Up-regulated |
| 446 | LOC729915 | -1.086691455 | 3.456288918 | -5.909923784 | 1.00E-06 | 0.00011534 | 5.274165029 | Up-regulated |
| 447 | PAG1 | -1.082844747 | 6.771803524 | -5.174037556 | 1.20E-05 | 0.00045825 | 3.225336078 | Up-regulated |
| 448 | JAK2 | -1.081529382 | 6.586145368 | -4.397866553 | 0.000113698 | 0.001942617 | 1.082787299 | Up-regulated |
| 449 | CYB5D1 | -1.080900331 | 4.695092197 | -3.234680255 | 0.002832372 | 0.016861198 | -1.945209302 | Up-regulated |
| 450 | MR1 | -1.079738434 | 6.197371563 | -4.92861402 | 2.50E-05 | 0.000720637 | 2.543257552 | Up-regulated |
| 451 | ATF3 | -1.07640793 | 4.090748836 | -2.928101438 | 0.006244115 | 0.02994916 | -2.673955353 | Up-regulated |
| 452 | DHCR7 | -1.076312775 | 4.174691543 | -3.586867268 | 0.001102872 | 0.009055506 | -1.065447017 | Up-regulated |
| 453 | SHKBP1 | -1.075551386 | 6.628865475 | -3.803407056 | 0.000608234 | 0.006087473 | -0.506037146 | Up-regulated |
| 454 | MTF1 | -1.074673799 | 8.489834602 | -5.429998171 | 6.00E-06 | 0.000281617 | 3.938435028 | Up-regulated |
| 455 | ACOX2 | -1.073857707 | 3.425849809 | -3.72970812 | 0.000745643 | 0.006967807 | -0.697823403 | Up-regulated |
| 456 | ACOT7 | -1.073514607 | 5.702640783 | -4.81600536 | 3.40E-05 | 0.000923553 | 2.231340546 | Up-regulated |
| 457 | DBN1 | -1.07335275 | 5.132741093 | -3.432306262 | 0.001675261 | 0.011934842 | -1.456546504 | Up-regulated |
| 458 | BRI3 | -1.072598039 | 9.801759234 | -6.407107097 | 0.00E+00 | 5. 11E-05 | 6.647385879 | Up-regulated |
| 459 | SLCO4C1 | -1.069523944 | 5.29663288 | -3.063575339 | 0.004420343 | 0.02322971 | -2.356671598 | Up-regulated |
| 460 | TMEM140 | -1.069229391 | 9.830293831 | -5.152256566 | 1.30E-05 | 0.000479065 | 3.164710542 | Up-regulated |
| 461 | LOC647195 | -1.066128919 | 5.361528055 | -4.568370857 | 7.00E-05 | 0.001443873 | 1.548925575 | Up-regulated |
| 462 | FLJ27255 | -1.064839452 | 3.461489761 | -5.209981329 | 1.10E-05 | 0.000427937 | 3.325410137 | Up-regulated |
| 463 | IGF1R | -1.063711476 | 3.524620104 | -4.466420213 | 9.30E-05 | 0.001719417 | 1.269777123 | Up-regulated |
| 464 | LMTK2 | -1.063613587 | 3.953310195 | -4.66292442 | 5.30E-05 | 0.00120546 | 1.808829443 | Up-regulated |
| 465 | HRB | -1.063556196 | 4.722367203 | -5.402749512 | 6.00E-06 | 0.000294151 | 3.862492137 | Up-regulated |
| 466 | ACVR1B | -1.062338838 | 6.577162331 | -4.171757429 | 0.000216497 | 0.003033573 | 0.47084 | Up-regulated |
| 467 | DNAJC5 | -1.060708378 | 5.351608967 | -4.314373624 | 0.000144328 | 0.002268769 | 0.855911937 | Up-regulated |
| 468 | SH2B2 | -1.059406823 | 4.924713818 | -4.604602742 | 6.30E-05 | 0.001334536 | 1.648410511 | Up-regulated |
| 469 | TNFRSF10D | -1.057523834 | 4.013184753 | -2.803374369 | 0.00853114 | 0.03739112 | -2.958849713 | Up-regulated |
| 470 | NUSAP1 | -1.056786436 | 4.554986976 | -6.660110958 | 0.00E+00 | 3.00E-05 | 7.339305394 | Up-regulated |
| 471 | CUX1 | -1.055220051 | 4.453698276 | -4.137208459 | 0.000238745 | 0.003260119 | 0.378068656 | Up-regulated |
| 472 | PSTPIP2 | -1.05492918 | 7.546895354 | -3.326882089 | 0.002219984 | 0.01428289 | -1.718914842 | Up-regulated |
| 473 | FPR1 | -1.053225554 | 11.8000279 | -5.414662722 | 6.00E-06 | 0.000286081 | 3.895694566 | Up-regulated |
| 474 | RPH3A | -1.053067482 | 3.577191298 | -3.008473514 | 0.005091087 | 0.025713861 | -2.486668752 | Up-regulated |
| 475 | C10orf119 | -1.05244415 | 4.923261051 | -4.167426665 | 0.00021917 | 0.003055672 | 0.459199443 | Up-regulated |
| 476 | MIIP | -1.052084138 | 5.329146045 | -2.975052952 | 0.005543697 | 0.027417604 | -2.564888203 | Up-regulated |
| 477 | H2AFJ | -1.052009842 | 9.146818987 | -4.308393421 | 0.00014681 | 0.002296305 | 0.839700656 | Up-regulated |
| 478 | REPS2 | -1.05099968 | 5.21326209 | -5.002935665 | 2.00E-05 | 0.000630567 | 2.749532588 | Up-regulated |
| 479 | HOMER3 | -1.045128751 | 3.84387099 | -3.79013728 | 0.000631007 | 0.006221706 | -0.54066937 | Up-regulated |
| 480 | COL18A1 | -1.04298464 | 5.967006275 | -3.88062246 | 0.000490777 | 0.005241904 | -0.303677202 | Up-regulated |
| 481 | IFITM2 | -1.041531702 | 13.03006864 | -5.196320918 | 1. 13E-05 | 0.000437855 | 3.287373254 | Up-regulated |
| 482 | FAM129B | -1.041028445 | 5.466735762 | -4.88709019 | 2.80E-05 | 0.000796114 | 2.428145261 | Up-regulated |
| 483 | MYD88 | -1.039823553 | 8.98001383 | -5.673749772 | 3.00E-06 | 0.00018385 | 4.617521691 | Up-regulated |
| 484 | OKL38 | -1.039815759 | 4.800251025 | -3.49000219 | 0.001434224 | 0.010767878 | -1.311415428 | Up-regulated |
| 485 | POTEE | -1.039695512 | 3.946468097 | -3.529902183 | 0.001287488 | 0.009994523 | -1.2104402 | Up-regulated |
| 486 | TMEM11 | -1.039695074 | 4.510110007 | -4.011868446 | 0.000339938 | 0.004125867 | 0.043364902 | Up-regulated |
| 487 | LOC642255 | -1.037147333 | 4.576818795 | -4.034266215 | 0.000319194 | 0.003952221 | 0.102951304 | Up-regulated |
| 488 | TCN2 | -1.036456941 | 3.918336488 | -3.960829648 | 0.000392261 | 0.004515829 | -0.092032695 | Up-regulated |
| 489 | LOC731954 | -1.036433249 | 6.235867748 | -3.381257897 | 0.001920673 | 0.012991112 | -1.584056969 | Up-regulated |
| 490 | MSRB2 | -1.036152423 | 8.775785337 | -4.160382768 | 0.000223587 | 0.003092513 | 0.440273326 | Up-regulated |
| 491 | MAOA | -1.034128336 | 3.457580614 | -3.384783605 | 0.001902667 | 0.012902232 | -1.575278131 | Up-regulated |
| 492 | LIPN | -1.033505762 | 3.576912793 | -3.594605427 | 0.001079862 | 0.00895262 | -1.045677585 | Up-regulated |
| 493 | KCND1 | -1.032639579 | 4.019769935 | -2.993896304 | 0.00528403 | 0.026490451 | -2.520845115 | Up-regulated |
| 494 | LOC641996 | -1.032510858 | 4.765494954 | -3.427518043 | 0.001696931 | 0.01201555 | -1.468543089 | Up-regulated |
| 495 | LOC642267 | -1.031284796 | 5.729485652 | -2.642378275 | 0.012647732 | 0.049657751 | -3.315529374 | Up-regulated |
| 496 | RALB | -1.030389054 | 9.895244736 | -4.300903443 | 0.000149979 | 0.002328518 | 0.819404214 | Up-regulated |
| 497 | TMEM180 | -1.028061407 | 4.082191312 | -3.531318741 | 0.001282555 | 0.009961772 | -1.20684637 | Up-regulated |
| 498 | LOC730234 | -1.026851891 | 6.140521523 | -2.929203617 | 0.006226756 | 0.029908333 | -2.671406182 | Up-regulated |
| 499 | CDK5RAP2 | -1.026769539 | 5.616340716 | -3.882879957 | 0.000487699 | 0.005229458 | -0.297739958 | Up-regulated |
| 500 | MOBK1B | -1.024947205 | 6.108491542 | -3.479064318 | 0.001477188 | 0.010972274 | -1.339010009 | Up-regulated |
| 501 | EGR1 | -1.024582058 | 5.696462953 | -4.999387963 | 2.00E-05 | 0.000630567 | 2.739679752 | Up-regulated |
| 502 | MAP4K4 | -1.024499993 | 6.36442827 | -3.304584476 | 0.002355243 | 0.01489708 | -1.77391942 | Up-regulated |
| 503 | PPARG | -1.024202741 | 3.421044489 | -4.544635212 | 7.50E-05 | 0.001508687 | 1.483829171 | Up-regulated |
| 504 | FLOT2 | -1.020774691 | 11.38837202 | -4.845208037 | 3. 13E-05 | 0.000863831 | 2.312149301 | Up-regulated |
| 505 | RAB24 | -1.020528917 | 8.171613689 | -4.308147445 | 0.000146913 | 0.002296305 | 0.839033974 | Up-regulated |
| 506 | ATG7 | -1.020310263 | 6.962296777 | -5.948426926 | 1.00E-06 | 0.000108935 | 5.381013396 | Up-regulated |
| 507 | LOC285550 | -1.020244891 | 5.229976428 | -5.428862474 | 6.00E-06 | 0.000281617 | 3.9352698 | Up-regulated |
| 508 | FCGR2A | -1.01857157 | 9.159930876 | -3.093321504 | 0.004093997 | 0.021944834 | -2.285971477 | Up-regulated |
| 509 | OSBPL1A | -1.017191938 | 5.127355189 | -4.551195401 | 7.30E-05 | 0.001492623 | 1.501814663 | Up-regulated |
| 510 | ZNF250 | -1.016296839 | 4.57720771 | -4.187323892 | 0.000207151 | 0.002917277 | 0.512707556 | Up-regulated |
| 511 | LOC441124 | -1.016105219 | 6.271190019 | -3.118986164 | 0.00383095 | 0.020961974 | -2.224684273 | Up-regulated |
| 512 | KL | -1.014033798 | 3.420060398 | -3.72183373 | 0.000761997 | 0.007041767 | -0.718232964 | Up-regulated |
| 513 | LOC732371 | -1.01262182 | 4.489859778 | -3.957410169 | 0.000396035 | 0.004544466 | -0.10108445 | Up-regulated |
| 514 | PLSCR4 | -1.012004066 | 4.34311847 | -2.656878291 | 0.012212422 | 0.048417165 | -3.283940744 | Up-regulated |
| 515 | PLP2 | -1.010980855 | 6.2349214 | -5.589372708 | 4.00E-06 | 0.000214651 | 4.382541901 | Up-regulated |
| 516 | MYO10 | -1.009761745 | 3.506716172 | -5.116600396 | 1.40E-05 | 0.000508352 | 3.065495588 | Up-regulated |
| 517 | CBARA1 | -1.009700968 | 5.956515573 | -4.880488736 | 2.80E-05 | 0.000806515 | 2.409854448 | Up-regulated |
| 518 | SECTM1 | -1.008638931 | 3.886784115 | -4.476698236 | 9.10E-05 | 0.001700088 | 1.297863428 | Up-regulated |
| 519 | NOD2 | -1.006418892 | 8.523709652 | -3.038701502 | 0.004712036 | 0.024331559 | -2.415511714 | Up-regulated |
| 520 | CHSY1 | -1.005772459 | 9.440921777 | -4.692709688 | 4.90E-05 | 0.001145491 | 1.890881713 | Up-regulated |
| 521 | TOMM40L | -1.005191522 | 5.826461819 | -4.321757246 | 0.00014132 | 0.002257096 | 0.875934992 | Up-regulated |
| 522 | RALGAPA2 | -1.004798851 | 4.244817168 | -3.276545911 | 0.002536562 | 0.015627376 | -1.842835955 | Up-regulated |
| 523 | KCNE1L | -1.002871116 | 3.511329694 | -3.820563364 | 0.000579975 | 0.005916492 | -0.461197907 | Up-regulated |
| 524 | TXN | -1.002706164 | 10.44887001 | -7.541732931 | 0.00E+00 | 7.00E-06 | 9.700076144 | Up-regulated |
| 525 | TRIP6 | -1.00266771 | 5.526138627 | -5.011734725 | 1.90E-05 | 0.000619553 | 2.773972322 | Up-regulated |
| 526 | RAB31 | -1.002300278 | 10.77818647 | -5.9855294 | 1. 14E-06 | 0.000104916 | 5.483909228 | Up-regulated |
| 527 | EXOC6 | -1.002278992 | 7.400148447 | -5.898984203 | 1.00E-06 | 0.000117682 | 5.243795035 | Up-regulated |
| 528 | B3GNT8 | -1.002264382 | 5.833023008 | -3.822229935 | 0.000577299 | 0.005900488 | -0.456838409 | Up-regulated |
| 529 | RHOG | -1.001966362 | 11.12132989 | -5.304931987 | 8.00E-06 | 0.000349759 | 3.589899657 | Up-regulated |
| 530 | SEPX1 | -1.001833572 | 11.80573924 | -5.756376002 | 2.00E-06 | 0.000159733 | 4.847460823 | Up-regulated |
| 531 | RBMS1 | -1.001531103 | 8.317568495 | -4.096652853 | 0.000267735 | 0.003501653 | 0.26944315 | Up-regulated |
| 532 | MAZ | -1.001262583 | 4.297610489 | -4.952347021 | 2.30E-05 | 0.00069433 | 2.609095123 | Up-regulated |
| 533 | PSENEN | -1.000651904 | 4.224491886 | -4.26166827 | 0.000167712 | 0.002519713 | 0.713224653 | Up-regulated |

**Supplementary Material 9:** Nodes degrees in PPI network construction (sorted in descending order by degree).

| **Nodes name** | **Degree** | **Average**  **Shortest**  **Path**  **Length** | **Betweenness**  **Centrality** | **Closeness**  **Centrality** | **Neighborhood**  **Connectivity** | **Radiality** | **Stress** | **Topological**  **Coefficient** |
| --- | --- | --- | --- | --- | --- | --- | --- | --- |
| **MMP9** | **31** | **1.088235** | **0.076853** | **0.918919** | **15.967742** | **0.997154** | **532** | **0.469639** |
| **TLR4** | **31** | **1.088235** | **0.099995** | **0.918919** | **15.483871** | **0.997154** | **574** | **0.455408** |
| PPARG | 29 | 1.147059 | 0.061774 | 0.871795 | 16.448276 | 0.995256 | 426 | 0.483773 |
| MAPK14 | 27 | 1.205882 | 0.048698 | 0.829268 | 16.851852 | 0.993359 | 356 | 0.495643 |
| ICAM1 | 27 | 1.205882 | 0.043775 | 0.829268 | 17.037037 | 0.993359 | 346 | 0.501089 |
| IGF1R | 23 | 1.323529 | 0.031151 | 0.755556 | 17.652174 | 0.989564 | 230 | 0.519182 |
| ARG1 | 23 | 1.323529 | 0.034291 | 0.755556 | 17.565217 | 0.989564 | 236 | 0.516624 |
| JAK2 | 21 | 1.382353 | 0.017023 | 0.723404 | 19.000000 | 0.987666 | 152 | 0.558824 |
| KDM6B | 18 | 1.470588 | 0.009456 | 0.680000 | 19.444444 | 0.984820 | 94 | 0.571895 |
| MMP8 | 17 | 1.500000 | 0.007177 | 0.666667 | 20.294118 | 0.983871 | 74 | 0.596886 |
| CXCR1 | 17 | 1.500000 | 0.012957 | 0.666667 | 18.941176 | 0.983871 | 98 | 0.557093 |
| HSPA1A | 17 | 1.500000 | 0.007068 | 0.666667 | 19.941176 | 0.983871 | 76 | 0.586505 |
| BCL2A1 | 17 | 1.500000 | 0.028309 | 0.666667 | 19.647059 | 0.983871 | 236 | 0.577855 |
| MAOA | 17 | 1.500000 | 0.015488 | 0.666667 | 19.294118 | 0.983871 | 108 | 0.567474 |
| CCR1 | 16 | 1.529412 | 0.009023 | 0.653846 | 19.062500 | 0.982922 | 72 | 0.560662 |
| PFKFB3 | 16 | 1.529412 | 0.024761 | 0.653846 | 19.937500 | 0.982922 | 204 | 0.586397 |
| ALPL | 15 | 1.588235 | 0.012140 | 0.629630 | 18.800000 | 0.981025 | 76 | 0.569697 |
| SELL | 15 | 1.558824 | 0.005810 | 0.641509 | 20.066667 | 0.981973 | 50 | 0.590196 |
| CYP1B1 | 14 | 1.617647 | 0.005303 | 0.618182 | 20.428571 | 0.980076 | 44 | 0.619048 |
| TOP2A | 13 | 1.617647 | 0.006898 | 0.618182 | 19.307692 | 0.980076 | 58 | 0.567873 |
| CA4 | 12 | 1.676471 | 0.006205 | 0.596491 | 19.500000 | 0.978178 | 50 | 0.590909 |
| MERTK | 12 | 1.676471 | 0.003028 | 0.596491 | 21.083333 | 0.978178 | 28 | 0.638889 |
| FABP5 | 12 | 1.647059 | 0.003735 | 0.607143 | 20.500000 | 0.979127 | 42 | 0.602941 |
| EGLN1 | 12 | 1.647059 | 0.001500 | 0.607143 | 22.416667 | 0.979127 | 16 | 0.659314 |
| EPHB4 | 11 | 1.705882 | 0.002385 | 0.586207 | 21.090909 | 0.977230 | 20 | 0.639118 |
| PYGL | 11 | 1.676471 | 0.004976 | 0.596491 | 19.545455 | 0.978178 | 32 | 0.574866 |
| LTB4R | 10 | 1.735294 | 0.000000 | 0.576271 | 23.300000 | 0.976281 | 0 | 0.706061 |
| BMX | 10 | 1.735294 | 0.002341 | 0.576271 | 20.600000 | 0.976281 | 22 | 0.624242 |
| CES1 | 9 | 1.735294 | 0.003181 | 0.576271 | 19.444444 | 0.976281 | 34 | 0.571895 |
| CDA | 9 | 1.735294 | 0.014020 | 0.576271 | 18.333333 | 0.976281 | 114 | 0.552189 |
| ECE1 | 8 | 1.794118 | 0.000000 | 0.557377 | 24.250000 | 0.974383 | 0 | 0.734848 |
| FUT7 | 6 | 1.852941 | 0.000000 | 0.539683 | 22.833333 | 0.972486 | 0 | 0.691919 |
| GBA | 5 | 1.882353 | 0.000777 | 0.531250 | 20.600000 | 0.971537 | 8 | 0.624242 |
| SCN9A | 4 | 1.911765 | 0.000000 | 0.523077 | 26.000000 | 0.970588 | 0 | 0.787879 |
| UPP1 | 3 | 2.235294 | 0.000611 | 0.447368 | 14.000000 | 0.960152 | 4 | 0.621212 |

**Supplementary Material 10:** Binding affinities in molecular docking analysis.

| **PubChem CID** | **Ingredients name** | **Affinity with MMP9 (kcal/mol)** | **Affinity with TLR4 (kcal/mol)** |
| --- | --- | --- | --- |
| 185617 | Scutellarin | -9.4 | -9.9 |
| 44566967 | isosalvianolic acid c | -10.1 | -8.8 |
| 13991590 | salvianolic acid c | -9.1 | -9.4 |
